# Tapping into technology and the biodiversity informatics revolution: updated terrestrial mammal list of Angola, with new records from the Okavango Basin

**DOI:** 10.3897/zookeys.779.25964

**Published:** 2018-08-02

**Authors:** Peter J. Taylor, Götz Neef, Mark Keith, Sina Weier, Ara Monadjem, Daniel M. Parker

**Affiliations:** 1 South African Research Chair on Biodiversity Value & Change and Core Team Member of the Centre for Invasion Biology, University of Venda, Thohoyandou 0950, South Africa University of VendaThohoyandouSouth Africa; 2 School of Life Sciences, University of KwaZulu-Natal Private Bag X54001, Durban 4000, South Africa University of KwaZulu-NatalDurbanSouth Africa; 3 National Geographic Okavango Wilderness Project, Wild Bird Trust, South Africa National Geographic Okavango Wilderness ProjectJohannesburgSouth Africa; 4 Eugène Marais Chair of Wildlife Management, Mammal Research Institute, University of Pretoria, Private Bag x20, Hatfield, Pretoria, 0028, South Africa University of PretoriaPretoriaSouth Africa; 5 Department of Biological Sciences, University of Swaziland, Private Bag 4, Kwaluseni, Swaziland University of SwazilandKwaluseniSwaziland; 6 Mammal Research Institute, Department of Zoology & Entomology, University of Pretoria, Private Bag x20, Hatfield, Pretoria, 0028, South Africa University of PretoriaPretoriaSouth Africa; 7 School of Biology and Environmental Sciences, University of Mpumalanga, Private Bag X11283, Nelspruit, 1200, South Africa University of MpumalangaNelspruitSouth Africa; 8 Wildlife and Reserve Management Research Group, Department of Zoology and Entomology, Rhodes University, Grahamstown, 6140, South Africa Rhodes UniversityGrahamstownSouth Africa

**Keywords:** Angola, checklist, Global Biodiversity Information Facility, mammals, Okavango Basin, scientific collections

## Abstract

Using various sources, including the Global Biodiversity Information Facility (GBIF), published literature, recent (2015–2017) collections, as well as bat detector and camera trap surveys with opportunistic sightings and live capture in the upper Okavango catchment in central Angola, we present an updated mammal checklist of 275 species from 15 different orders for Angola (including the Cabinda region). Recent surveys (captures and bat detectors) of small mammals from the upper Okavango catchment yielded 46 species (33 species of bats, ten species of rodents and three species of shrews). One bat (*Pipistrellusrusticus*, rusty pipistrelle); two rodents (*Mussetzeri*, Setzer’s mouse and *Zelotomyswoosnami*, Woosnam’s broad-faced mouse) and one shrew (*Suncusvarilla*, lesser dwarf shrew) were captured for the first time, in Angola. While our species lists of bats conformed to predicted totals, terrestrial small mammals were under sampled, with only 13 species recorded by our trapping survey compared to a total of 42 shrew and rodent species expected based on GBIF records for the central Angolan highlands. Seven terrestrial small mammal species (one shrew and six rodents) are endemic to the central and western Angolan highlands but none of these were captured in our survey. The bat detector surveys added three further bat species to the country list: *Pipistrellushesperidus*, *Kerivoulaargentata*, and *Mopsmidas*. Camera trap surveys and opportunistic sightings in the upper Okavango catchment in 2016 yielded a total of 35 species of medium-large mammals, from 17 families, although all of these had been reported previously in Angola. GBIF proved to be an excellent source of biodiversity data for Angolan mammals, most importantly for documenting dramatic historical range changes of larger mammals such as the sable (*Hippotragusnigerniger*), Kirk’s sable (*H.nigerkirkii*) and the giant sable (*H.nigervariani*).

## Introduction

Country species checklists and distribution maps for key taxa such as mammals represent a critical step in national efforts towards reaching international (e.g., the Convention on Biological Diversity) and national biodiversity targets and planning for conservation management and sustainable development at regional and local levels. Rapid advances in biodiversity informatics leading to huge volumes of reliable historical and recent occurrence data through public portals such as the Global Biodiversity Information Facility (https://www.gbif.org) make it possible to conduct taxonomic and conservation biodiversity assessments and compile reliable annotated species lists even for poorly known countries and regions (Soberón and Peterson 2004; Beaman and Cellinese 2012; Coetzer 2012; Wieczorek et al. 2012).

At the same time, advances in technology such as camera traps and microphones (including bat detectors) and associated analytical tools are facilitating rapid and efficient field inventories of groups such as larger mammals, bats, birds, crickets, and amphibians. In many cases, acoustic systems have been developed for automated species classification of huge volumes of call data. In the case of bats, echolocation calls are not songs, making the identification to species from bat calls a challenging exercise that requires suitable cross-testing of results using reliably identified calls, e.g., from captured and released individuals (Barclay 1999; Taylor et al. 2013; Monadjem et al. 2017; Rydell et al. 2017). Similarly, camera traps have enabled efficient and comprehensive surveys of medium and large-sized mammals and other groups (Stein et al. 2008; Tobler et al. 2008; Rovero and Marshall 2009; Rovero et al. 2014).

The objectives of this study were firstly to consolidate available data to compile an updated species checklist of terrestrial mammals of Angola, and secondly to add to this list the results of recent surveys of mammals in the poorly surveyed Okavango catchment area of the central highlands of Angola, using both live capture and remote camera trap and acoustic techniques.

**Brief historical overview of mammal collections from Angola**. Based largely on a collection of 2,300 specimens from the south-western quarter of Angola in the American Museum of Natural History from the Vernay-Lang (conducted in 1925) and Phipps-Bradley (in 1932) expeditions, Hill and Carter (1941) listed a total of 223 species of mammals for Angola, including 13 shrews (Soricomorpha), one hedgehog (Erinaceomorpha), one golden mole and one otter shrew (Afrosoricida), two elephant shrews (Macroscelidea), one aardvark (Tubulidentata), two pangolins (Pholidota), 53 bats (Chiroptera), 10 primates (Primates), two hares (Lagomorpha), 66 rodents (Rodentia), 36 carnivores, one elephant (Proboscidea), two hyraxes (Hyracoidea) and 32 ungulates (four Perissodactyla and 28 Artiodactyla). Subsequent to this publication, Angola has been largely neglected in terms of mammal survey effort. For example, those who led the Smithsonian Institution’s ambitious African Mammal Project (1961–1972), which collected 63,213 voucher specimens from throughout Africa and led to the definitive "Mammals of Africa: An Identification Guide" (Meester and Setzer 1971), did not visit Angola at all (Schmidt et al. 2008). Crawford-Cabral and co-authors compiled a database of just under 10,000 records (hosted by the University of Lisbon, Instituto de Investigação Científica Tropical in Portugal) mainly from 1930–1980 of 140 species and subspecies of carnivores, ungulates, and rodents collected from Angola (Crawford-Cabral 1998; Crawford-Cabral and Simões 1987, 1988; Crawford-Cabral and Veríssimo 2005).

The Lubango Museum, originally housed by Instituto de Investigação Científica de Angola (IICA) and currently housed at the the Instituto Superior da Ciências e Educação (ISCED) comprises about 4,000 mammal specimens of at least 123 species from Angola (https://www.gbif.org/publisher/975daf99-f28c-4201-86f2-2bfce0cba085). Another important museum in Angolan history is that established in Dundo. This was a relatively well-stocked museum and was in an important location (in the far northeast) for tropical species. There are at least two important papers by A Monard in 1931 and 1935 (cited in Hayman 1963) that detail the bat species in that collection (as well as other mammals). The small mammals in this collection were reviewed by Hayman (1963) and he mentioned 602 specimens that he examined (apparently these were shipped to him in London) belonging to 91 species and subspecies (including some 14 not previously reported for Angola).

It is little appreciated that Angola was actually relatively well known compared with East Africa until the turn of the 20^th^ century. Early explorers and scientists such as Bocage made enormous contributions. Bocage described 25 Angolan taxa based on new collections between 1878 and 1890, and seven Angolan taxa were named after him by other scientists (Hill and Carter 1941). Of the taxa described by Bocage, although most have been relegated to synonyms or subspecies in current lists, at least eight currently recognized Angolan mammals were named by him: the Angolan fruit bats *Epomophorusangolensis* and *Epomopsdobsoni*, the murid rodents *Myomyscusangolensis* and *Otomysanchietae*, the squirrel *Funisciurusbayoni*, the gerbil *Gerbilliscusvalidus*, the genet *Genettaangolensis* and the mongoose *Herpestesflavescens*.

Notwithstanding changes in taxonomy and the relative lack of survey effort, the list of species known to occur in Angola has increased, particularly in the case of small mammals. A relatively recent synthesis of Angolan murid rodents was published by Crawford-Cabral (1998) after an older synthesis of bats (Crawford-Cabral 1986). More recently, biogeographical and taxonomic syntheses of African bats (Monadjem et al. 2010a) and rodents (Monadjem et al. 2015) have listed 60 bat species and 78 rodent species from Angola, representing species richness increases of 13% and 18%, respectively, in comparison to Hill and Carter (1941).

**Figure 1.**
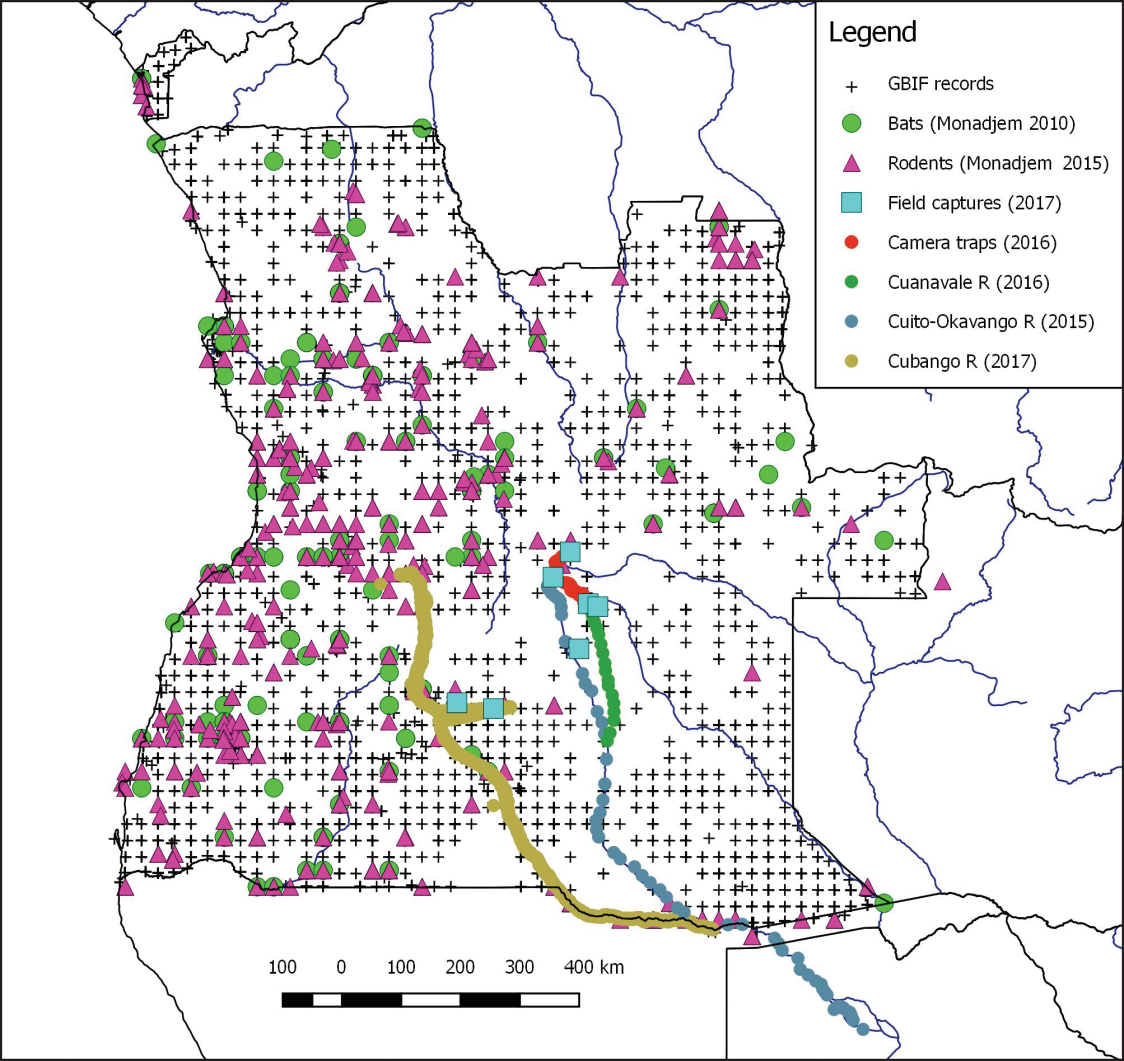
Map of Angola showing spatial occurrence of mammal records obtained from various sources, including the present study which reported on captures of small mammals and acoustic recordings of the echolocation calls of bats. Although the Cuito-Okavango River trip of 2015 extended beyond Angola into Namibia and Botswana, there were no species identified from acoustic data in Namibia and Botswana that were not also detected in Angola.

## Materials and methods

### Updating of mammal list for Angola

We combined records from the literature (Hill and Carter 1941; Hayman 1963; Crawford-Cabral 1986, 1998; Crawford-Cabral and Simões 1987, 1988; Crawford-Cabral and Veríssimo 2005; Monadjem et al. 2010a, 2015) with records obtained from a search of the GBIF portal (www.gbif.org) conducted on 18 December 2017 (GBIF.org 2017), which yielded 14,275 records based on 31 databases (Suppl. material 1). The two main institutions contributing data were the Instituto de Investigação Científica Tropical in Portugal (8,977 records) which incorporates the works of Crawford-Cabral (1998); Crawford-Cabral and Simões (1987, 1988) and Crawford-Cabral and Veríssimo (2005), and the American Museum of Natural History (2,240 records), which incorporates the survey of Hill and Carter (1941). Other important contributors include the Field Museum of Natural History (1,223 records) and The Natural History Museum in London (895 records). Together, these four databases comprised 93% of all records. We added records from the 2016 collection of 68 small mammals (bats, rodents and shrews) from the Okavango catchment of Angola deposited in the Durban Natural Science Museum (DNSM). The DNSM mammal collection also yielded an additional 14 records of Angolan rodents collected by S. Eiseb and J. Jarvis. Finally, we also added records of bat species determined by acoustic bat detector and capture surveys, shrews and rodents by live trapping methods and medium to large mammals recorded by camera traps supplemented with verifiable opportunistic sightings or signs.

To compile an updated species list based on the above sources, we adopted the taxonomy of Wilson and Reeder (2005) or more recent taxonomic treatments for certain groups, e.g., Monadjem et al. (2010a) and the 2016 African Chiroptera Report for bats (ACR 2016), Monadjem et al. (2015) and Denys et al. (2017) for rodents, and species accounts from all volumes of the series on Mammals of Africa (Kingdon et al. 2013a). We also tested the current taxonomic validity of each name using the Interagency Taxonomic Information System (www.itis.org) and the Mammal Diversity Database of the American Society of Mammalogists (Mammal Diversity Database 2018https://mammaldiversity.org). Species of dubious occurrence were defined as those having only a single record and/or collector, no clear basis for identification, occurring well outside their known range, and not included in authoritative texts for Angola (Hill and Carter 1942; Hayman 1963; Crawford-Cabral 1986; Crawford-Cabral 1998; Crawford-Cabral and Simões 1987; 1988; Crawford-Cabral and Veríssimo 2005; Monadjem et al. 2010a, 2015). Dubious species were flagged as such, including the reason for their exclusion. As mentioned above, bat species records based only on acoustic data were also added to this list.

**Figure 2.**
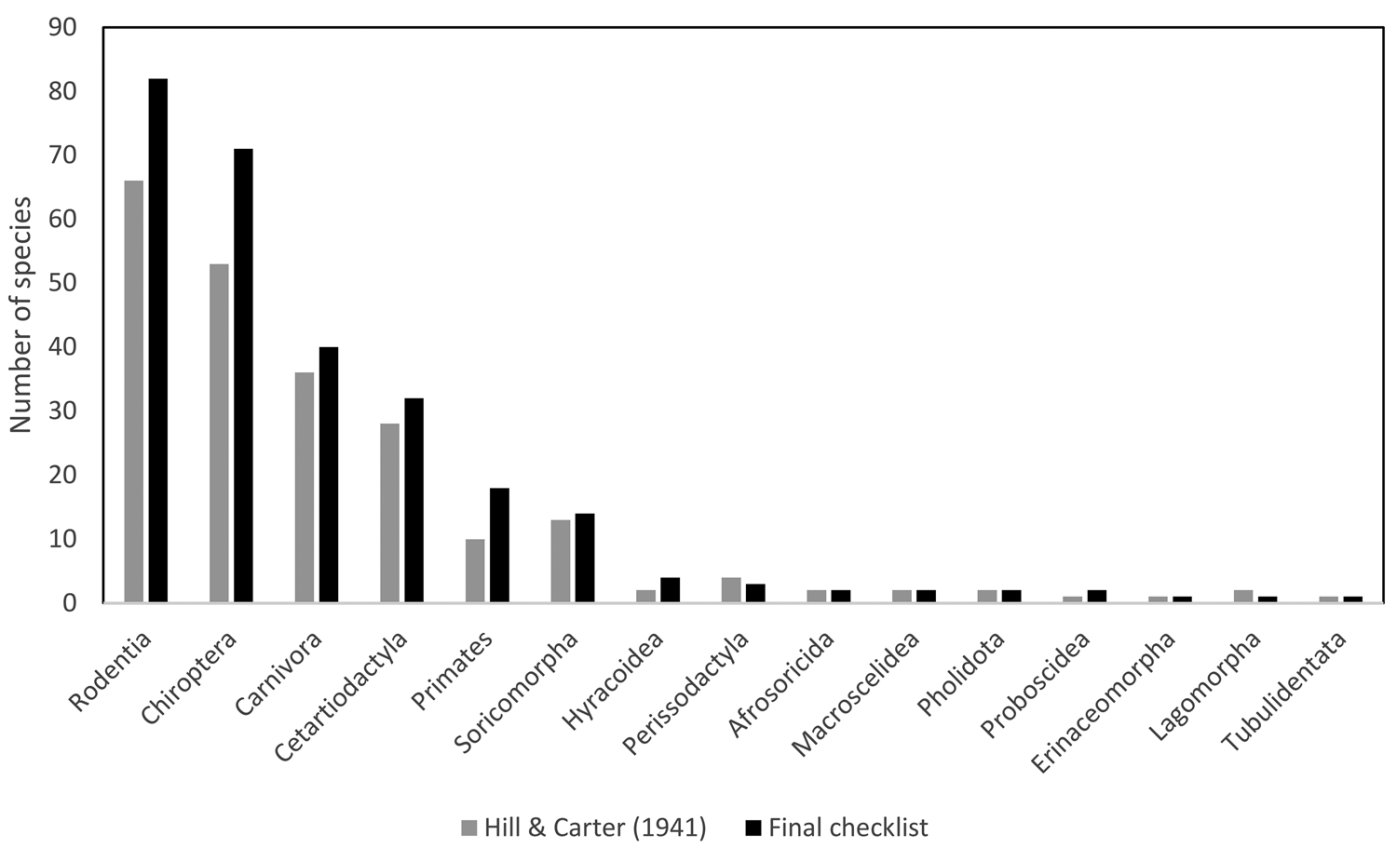
Histogram summarising species number of Angolan terrestrial mammals per order based on Hill and Carter (1943) and the current study.

### Sampling of upper Okavango catchment region

Sampling of small mammals from the highlands of Angola using both capture and acoustic techniques was carried out in 2013 (acoustic only), 2015 (acoustic only), 2016 (capture and acoustic) and 2017 (acoustic only).

*Capture survey.* Between 16 and 22 March 2016, and 29 October and 4 November 2016, bats were captured using one to three mist nets (Ecotone; 6 m, 9 m and 12 m) set per night, a two-bank harp trap (“Austharp”, Faunatech), and via searches for roosting bats, e.g., under the loose bark of trees. During the two 2016 field periods, as well as opportunistically on other occasions, shrews and rodents were captured using standard Sherman live-traps, usually 25–30 per night, set 5–10 m apart in a line. Some shrews were captured in the herpetofauna drift fence arrays. Two bats were collected opportunistically in 2013 and 2016 by W. Conradie.

*Acoustic recordings of bats*. Using four different bat detectors, we obtained recordings for a total of 208 detector-nights between 2015 and 2017. Apart from monitoring of several point localities, regular (mostly nightly) acoustic monitoring was carried out during three canoe river journeys down the length of the Cuanavale, Cuito, Cubango and Okavango Rivers, totalling a 2,744 km transect. During the 2015 field season, passive acoustic recordings were obtained with an EM3 bat detector (Wildlife Acoustics, Concorde, USA) for 75 nights (between 23 May and 18 September 2015) during a river expedition starting at the Cuito source and continuing to the Okavango Delta. During the early 2016 field season, passive acoustic recordings of bat echolocation calls were obtained using two Song Meter SM2BAT+ bat detectors (Wildlife Acoustics) and an ANABAT SD2 bat detector (Titley Electronics). Passive recording was carried out for six nights in March 2016 with an SM2BAT+ detector and three nights with ANABAT SD2 (in both cases between 15 and 21 March 2016), from the Cuito and Cuanavale source areas, Cuchi Gorge and Samboana Village. Additionally, recordings were obtained for 43 nights in total (between 17 February and 4 April 2016), of which 37 nights yielded calls, during a river trip from the source of Cuanavale River to its confluence with the Cuito River. Passive recordings were obtained between 21 October and 4 November 2016 (eight nights) using an SM2BAT+ detector, from 27 October to 4 November 2016 (10 nights) with an ANABAT SD2 detector and from 27 October to 2 November 2016 (six nights) with an EM3 detector. During the late 2016 season, recordings focussed on the Cuanavale and Saliakwembo source areas. During 2017, recordings were taken for a period of 1–2 hours nightly for a total of 51 nights with an ANABAT SD1 detector during a river trip down the Cubango River.

The following approach was used to identify bat calls to species or species-groups. Call analysis and identification was undertaken independently by three observers (PJT, MK and DP) although consensus was later obtained on the definition of call parameters for each species after extensive consultation and comparison of calls by PJT. Based on this, a final species list was derived by PJT (Table 1). Using Kaleidoscope Pro software (Wildlife Acoustics), the wave files collected with two SMBAT2+ detectors during the early 2016 season (February to April 2016), both from the Cuanavale River transect and from recordings by PJT in the Cuanavale and Cuito source areas, were firstly converted to zero-crossing (ANABAT) files and then identified manually by PJT using AnalookW software (version 4.1t, Chris Corbin, www.hoarybat.com), for comparison with zero-crossing ANABAT calls obtained directly from ANABAT SD1 and SD2 detectors. The latter included recordings from a SD2 bat detector from the Cuanavale and Cuito source lakes area and Cuchi Gorge in early 2016, as well as those from an Anabat SD1 detector during the 2017 transect of the Cubango River. Identifications were based on reference calls obtained both from ANABAT calls from captured and field-identified individuals that were subsequently released, as well as from standard references (Monadjem et al. 2010, 2017; Happold and Happold 2013; Taylor et al. 2013) and unpublished call data (Taylor, unpublished, Monadjem, unpublished). Using the same identification criteria, calls recorded from the Cuanavale and Saliakembo source lakes in late 2016 using EM3, SM2+ and Anabat SD2 detectors were identified by MK, while calls recorded with the EM3 detector during the 2015 transect of the Cuito and Okavango Rivers were identified by DP using a different software (SonoBat). After extensive comparisons of calls and consultation between PJT, MK, and DP, PJT derived a standard list of putative bat species. This library of identified calls is available on request from PJT.

**Table 1.** List of small mammal species collected in the central region of Angola in 2013 (four specimens) and 2016 (64 specimens) and deposited in the Durban Natural Science Museum. All specimens were checked by PJT based on cleaned skulls and skins in ethanol.

Species	Common name	IUCN status	Localities recorded
**Order Chiroptera**			
* Plerotes anchietae *	Anchieta’s Broad-Faced Fruit Bat	Data Deficient	Cuanavale Source
* Epomophorus angolensis *	Angolan Epauletted Fruit Bat	Near Threatened	Cuchi Gorge
* Epomops dobsonii *	Dobson’s Epauletted Fruit Bat	Least Concern	Sambojana, Saliakembo
* Hypsugo anchietae *	Anchieta’s pipistrelle	Least Concern	13 km north of Chett
*Pipistrellusrusticus	Rusty Pipistrelle	Least Concern	Cuito Source, Sambojana, Cuanavale Source, Saliakembo
* Neoromicia zuluensis *	Zulu Pipistrelle	Least Concern	Cuanavale Source, Saliakembo Source
* Neoromicia capensis *	Cape Serotine	Least Concern	Cuanavale Source, Saliakembo Source
**Laephotisangolensis	Angolan Long-Eared Bat	Data Deficient	Cuanavale Source, Sambojana, Saliakembo Source
* Scotophilus leucogaster *	White-bellied House Bat	Least Concern	Saliakembo Source
* Mimetillus thomasi *	Thomas’s flat-headed bat	Not assessed	Cuanavale Source, Saliakembo Source
* Chaerephon nigeriae *	Nigerian Free-Tailed Bat	Least Concern	Cuanavale Source
**Order Soricomorpha**			
* Crocidura fuscomurina *	Bicolored musk shrew	Least Concern	Mupapa Falls
* Crocidura hirta *	Reddish-Grey Musk Shrew	Least Concern	Cuanavale Source, Saliakembo Source
* *Suncusvarilla*	Lesser Dwarf Shrew	Least Concern	En route to Sambojana
**Order Rodentia**			
**Otomysanchietae	Angolan Vlei Rat	Least Concern	Cuito Source
Rhabdomys cf. dilectus	Striped Mouse	Least Concern	Cuanavale Source
* Mastomys natalensis *	Multimammate mouse	Least Concern	Cuanavale Source Lake
*Mussetzeri	Setzer’s Mouse	Least Concern	Cuanavale Source, Cuito Source Cunde Falls
* Lemniscomus griselda *	Single-Striped Mouse	Least Concern	25 km west of Menongue
*Zelotomyswoosnami	Woosnam’s Broad-Faced Mouse	Least Concern	Cuito Source
* Gerbilliscus leucogaster *	Lowveld Gerbil	Least Concern	Cuanavale Source, Cunde Falls
* Graphiurus kelleni *	Dormouse	Least Concern	Cuito Source
* Saccostomus campestris *	Pouched mouse	Least Concern	Cuanavale Source, Cuito Source, Sambojana
* Steatomys krebsii *	Fat Mouse	Least Concern	Cuanavale Source, Cuito Source, Mupapa Falls

* New record for Angola ** Angola endemic.

*Camera trapping*. The medium and large mammal surveys were conducted using two systematic camera-trapping assessments during 2016. The first camera trapping survey ran between February and March 2016 (936 trap nights) and the second ran between July and November 2016 (1,349 trap nights).

In the first camera trapping survey, two sites near the southern end of the Bie and Moxico provinces were sampled using linear transects. Transect 1 (north-west orientation along the Cuanavale and Cuando Rivers) was approximately 56 km long and was separated by ca. 20 km from transect 2 (south-east orientation just south of the town of Munhango) which was 50 km long. We sampled 19 camera stations along transect 1 and 20 camera stations along transect 2, with each station spaced ca. 3 km (range: 2–4 km) apart. One camera trap was placed at each station and was either a Cuddeback C2 (n = 11), E3 (n = 8) (Non-Typical, Inc. Green Bay, Wisconsin) or Bushnell Trophy Cam Aggressor HD (n = 20) (Bushnell Outdoor Products, Cody, Kansas). Cameras were placed on animal paths about 30cm high on the base of the tree or on a stake placed in the ground to maximize photographic captures of the full range of mammalian body sizes (Mann et al. 2015). Cameras were set to take three sequential photographs per trigger, with an interval of one minute between trigger events. Sensitivity of the sensor was set on medium (normal) and picture quality was set to 5MP. Cameras were operational for 24 hours a day and were checked only upon collection.

The second camera trapping survey employed a similar approach. However, in this survey 17 camera stations were sampled along the Cuanavale River south-east from the village of Tchijanga towards the Quembo River. The transect was approximately 120 km long and camera traps (13 Cuddeback and four Bushnell) were again placed on well used animal paths, but with no specific distance between each camera station (range: 1–20 km).

The date and GPS locations of any verifiable sightings and signs of medium and large mammals were recorded during each expedition. Analyses of photographs from each camera trap survey were limited to those photographs taken from 12:01am the day after setting up a camera trap until 12:00pm of day before camera trap stopped recording. To ensure independence of capture events for each camera trap, images of individuals of the same species were ignored if captured within one hour of a previous sighting (O’Brien et al. 2003). For each photograph we recorded the site, date, time, and species. We excluded sightings of birds, small (< 1 kg) mammals, domestic animals, people, and any unidentifiable images. The total number of capture events (n) per species was tallied and their percentage contribution (spp %) to the total number of photographs was calculated. The capture frequency (CF) for each species was calculated as the number of capture events (n) per 100 camera-trapping days (Tobler et al. 2008).

## Results

### Mammal list for Angola

An updated species checklist of 275 species of Angolan mammals is presented in Suppl. material 1, comprising 245 species records obtained from GBIF and an additional 30 species added from the literature and recent surveys (additional records marked in bold in Suppl. material 1). The list excludes commensal and domesticated species. Of this total, nine bat species were added to the revised GBIF list by Monadjem et al. (2010a), four rodent species were added by Monadjem et al. (2015), and additional bat, rodent and shrew species were added by the current survey (see paragraph below).

### New small mammal capture data from Okavango Basin

We collected 68 specimens of 24 species of small mammals (three shrew, ten rodent, and 11 bat species; Table 1). Of these species, one bat (*Laephotisangolensis*, the Angolan long-eared bat) and one rodent (*Otomysanchietae*, the Angolan vlei rat) are Angolan endemics (Table 1). One bat (*Pipistrellusrusticus*, rusty pipistrelle), two rodent species (*Mussetzeri* Setzer’s mouse and *Zelotomyswoosnami* Woosnam’s broad-faced mouse) and one shrew (*Suncusvarilla*, lesser dwarf shrew) were recorded for the first time in Angola, all listed as Least Concern, by the IUCN Red List. Two of the bat species were listed as Data Deficient (*Plerotesanchietae*, Anchieta’s broad-faced fruit bat and Angolan long-eared bat) and one is classified as Near Threatened (*Epomophorusangolensis*, Angolan epauletted fruit bat), while one was not assessed.

There was a very marked difference in bat activity between March 2016 (which was characterised by heavy rainfall), when only 13 bats were caught, and October/November 2016 (less frequent rainfall) when 151 bats were collected, in spite of very similar trap/net effort and six nights sampled for both periods. Captures in late 2016 were dominated by *Pipistellusrusticus*, *Neoromiciacapensis*, and *N.zuluensis*.

### New acoustic data from the Okavango Basin

From manual identification of zero-crossing calls, we identified a total of 29 putative species of insectivorous bats (Tables 2, Suppl. material 2).

### Diversity and importance of small mammals in Angola and the Okavango source lakes region

The current capture survey adds one bat species, two rodent species and one shrew species to the country lists above, bringing to 67 and 87 the total number of Angolan bat and rodent species. From our current survey, we recorded 13 non-volant small mammals from the upper Okavango catchment of Angola, and some 33 species of bats, based on the estimate of insectivorous species from acoustic data (29 species; see above) combined with one rare species (*Mimetillusthomasi*, Thomas’s flat headed bat), for which we have no echolocation data, and three additional species of fruit bats captured with mist nets/harp-traps (Table 1).

### Camera trap data from the Okavango source lakes region

A total of 35 species of medium-large mammals, from 17 families were recorded in 2016 through opportunistic sightings and two formal camera-trapping surveys (Table 3). Ten species were recorded by both camera surveys and opportunistic sightings (common duiker, large-spotted genet, honey badger, side-striped jackal, serval, spotted hyena, porcupine, scrub hare, and vervet monkey; see Table 3 for scientific names). Eleven species were recorded by the camera traps only (greater bushbaby, tree squirrel, springhare, aardwolf, caracal, African wild cat, lion, aardvark, blue duiker and steenbok). Five species were only detected opportunistically during expeditions (African elephant, oribi, roan, lechwe and sitatunga).

**Table 2.** Putative bat species definitions based on analysis of bat calls from various acoustic surveys in the Okavango catchment of Angola between 2015 and 2017. Although the Cuito-Okavango River trip of 2015 extended beyond Angola into Namibia and Botswana, there were no species identified in Namibia and Botswana that were not also detected in Angola. Matching of calls with species was based on release calls from bats captured and released in Angola (for *Laephotisangolensis*, *Neoromiciacapensis*, *Pipistrellusrusticus* and *N.zuluensis*) as well as Monadjem et al. 2010a, Happold and Happold 2013, Taylor et al. 2013a and unpublished data from PJT and AM. Known occurrence of species in Angola is shown based on previous evidence from specimens (based on the current survey, GBIF records or listed as such by Monadjem et al. 2010) or only from the literature. The occurrence of a species is shown as “predicted” where records are known from adjacent countries and high probabilities of occurrence were indicated for any part of Angola in Maximum Entropy Modeling (MaxEnt for short) species models depicted in Monadjem et al. (2010a). R = River.

Species	Functional group (Monadjem et al. 2010a)	Overlap species	Evidence for occurrence in Angola	Caught in current survey	No. calls (Cuanavale R)	No. nights (Cuanavale R)	Cuito-Okavango R (2015)	Cuanavale R (2016)	Source lakes (early 2016)	Source lakes and Cuchi Gorge (2016)	Source Lakes (late 2016)	Cubango R. (2017)
**Family Emballonuridae**												
* Coleura afra *	Open-air	Possibly *T.perforatus* (unrecorded)	Specimen	No	29	7	√	√	√			√
* Taphozous mauritianus *	Open-air	*C.pumilus*, *T.aegyptiaca*	Specimen	No	272	24		√			√	√
**Family Hipposideridae**												
* Macronycteris vitattus *	Clutter	None	Specimen	No	0	0					√	
**Family Rhinolophidae**												
* Rhinolophus fumigatus *	Clutter	None	Specimen	No	14	5		√	√		√	
**Family: Miniopteridae**												
Miniopterus cf. fraterculus	Clutter-edge	* M. fraterculus *	Acoustic evidence only	No	20	12	√	√	√	√	√	√
* Miniopterus natalensis *	Clutter-edge	* P. rusticus *	Specimen	No	55	19	√	√				
**Family Molossidae**												
* Chaerephon ansorgei *	Open-air	*T.ventralis*, *C.nigeriae*, *T.fulminans*	Specimen	No	0	0	√					
* Chaerephon nigeriae *	Open-air	*T.ventralis*, *C.ansorgei*, *T.fulminans*	Literature	Yes	6	3		√			√	√
* Chaerephon pumilus *	Open-air	*T.aegyptiaca*, *M.condylurus*, *T.mauritianus*	Specimen	No	79	12	√	√		√	√	√
* Mops condylurus *	Open-air	*C.pumilus*, *T.aegyptiaca*	Specimen	No	21	9	√	√		√	√	√
* Mops midas *	Open-air	* C. nigeriae *	Predicted	No	2	2	√	√			√	√
* Otomops martiensseni *	Open-air	None	Specimen	No	1	1		√				√
* Tadarida aegyptiaca *	Open-air	*T.mauritianus*, *C.pumilus*	Specimen	No	101	18	√	√		√	√	√
Mops cf. condylurus	Open-air	* M. condylurus *	Calls do not match any known species	No	23	3		√		√	√	
**Family: Vespertilionidae**												
* Eptesicus hottentotus *	Clutter-edge	*S.dinganii*, *M.welwitschii*	Specimen	No	8	4		√			√	√
* Hypsugo anchietae *	Clutter-edge	* M. natalensis *	Specimen	Yes	80	22	√	√	√	√	√	√
* Kerivoula argentata *	Clutter-edge	None	Predicted	No	0	0		√			√	√
* Kerivoula lanosa *	Clutter-edge	None	Specimen	No	0	0					√	
* Laephotis angolensis *	Clutter-edge	* P. hesperidus *	Specimen	Yes	2	2					√	√
* Myotis welwitschii *	Clutter-edge	* M. bocagii *	Specimen	No	16	9		√	√		√	
* Neoromica zuluensis *	Clutter-edge	* P. hesperidus *	Specimen	Yes	43	10		√	√	√	√	√
* Neoromicia capensis *	Clutter-edge	*S.viridis*, *S.hindei*/*S.albigula*, *N.schlieffeni*	Specimen	Yes	29	13	√	√		√	√	√
* Neoromicia nana *	Clutter-edge	None	Specimen	No	0	0			√	???		√
Neoromicia cf. nana	Clutter-edge	* N. nana *	Calls do not match any known species	No	59	4	√		√	√		√
* Nycticeinops schlieffeni *	Clutter-edge	*S.viridis*, *S.hindei*/*S.albigula*, *N.capensis*	Specimen	No	124	14	√	√	√	√	√	√
* Pipistrellus hesperidus *	Clutter-edge	*N.zuluensis*, *L.angolensis*	Predicted	No	805	25	√	√	√	√	√	√
* Pipistrellus rusticus *	Clutter-edge	* M. natalensis *	Predicted	Yes	161	25	√	√	√	√	√	√
* Scotophilus dinganii *	Clutter-edge	*E.hottentotus*, *L.botswanae*	Specimen	No	28	7		√	√	√	√	√
* Scotophilus leucogaster *	Clutter-edge	*N.schlieffeni*, *S.viridis*, *N.capensis*	Specimen	Yes	28	17		√	√	√		

**Table 3.** All medium-large mammal species detected opportunistically (see methods) and during two formal camera-trapping surveys of the south-eastern highlands of Angola in 2016. Where applicable, the total number of photographic events per species (n), their percentage contribution (Spp. %) to the total number of photographic events, and the capture frequency (CF) (number of events/100 camera days) is shown. V = verified opportunistic sighting, C1 = camera trap survey 1, C2 = camera trap survey 2. If a species was recorded in both camera-trapping surveys, the values for both surveys are separated by a backslash (i.e., C1/C2).

Family	Species	Common name	V	C1	C2	n	Spp. %	CF
Galagidae	* Otolemur crassicaudatus *	Greater bushbaby		√		3	2.5	0.3
Cercopithecidae	* Chlorocebus cynosuros *	Vervet monkey	√	√	√	10/3	8.5/0.6	1.1/0.2
Leporidae	* Lepus victoriae *	Africa savanna hare	√	√	√	8/7	6.8/1.5	0.9/0.5
Sciuridae	* Paraxerus cepapi *	Tree squirrel		√		1	0.9	0.1
Pedetidae	* Pedetes capensis *	Springhare			√	11	2.3	0.8
Hystricidae	* Hystrix africaeaustralis *	Porcupine	√	√	√	2/8	1.7/1.7	0.2/0.6
Protelidae	* Proteles cristatus *	Aardwolf		√		1	0.9	0.1
Hyaenidae	* Crocuta crocuta *	Spotted hyena	√	√	√	2/6	1.7/1.2	0.2/0.4
Felidae	* Acinonyx jubatus *	Cheetah		√	√	3/1	2.5/0.2	0.3/0.1
	* Caracal caracal *	Caracal			√	3	0.6	0.2
	* Felis lybica *	African wildcat			√	1	0.2	0.1
	* Leptailurus serval *	Serval	√	√	√	1/1	0.9/0.2	0.1/0.1
	* Panthera leo *	Lion			√	1	0.2	0.1
	* Panthera pardus *	Leopard	√		√	48	10.0	3.6
Canidae	* Lycaon pictus *	African wild dog	√		√	8	1.7	0.6
	* Canis adustus *	Side-striped jackal	√	√	√	4/13	3.4/2.7	0.4/1.0
Mustelidae	* Mellivora capensis *	Honey badger	√	√	√	1/4	0.9/0.8	0.1/0.3
	* Ictonyx striatus *	Striped polecat (Zorilla)	√		√	1	0.2	0.1
Viverridae	* Civettictis civetta *	African civet	√	√		3	2.5	0.3
	* Genetta maculata *	Large-spotted genet	√	√	√	25/3	21.2/0.6	2.6/0.2
Herpestidae	* Atilax paludinosus *	Marsh mongoose	√		√	1	0.2	0.1
	* Ichneumia albicauda *	White-tailed mongoose		√	√	2/9	1.7/1.9	0.2/0.7
	* Mungos mungo *	Banded mongoose		√		1	0.9	0.1
Orycteropodidae	* Orycteropus afer *	Aardvark			√	4	0.8	0.3
Elephantidae	* Loxodonta africana *	African elephant	√			–	–	–
Suidae	* Potamochoerus porcus *	Bushpig	√	√	√	1/24	0.9/5.0	0.1/1.8
	* Phacochoerus africanus *	Warthog	√		√	5	1.0	0.4
Bovidae	* Cephalophus silvicultor *	Silver-backed duiker		√	√	5/14	4.2/2.9	0.5/1.0
	* Philantomba monticola *	Blue duiker		√	√	4/19	3.4/3.9	0.4/1.4
	* Sylvicapra grimmia *	Common duiker	√	√	√	23/285	19.5/59.1	2.5/21.1
	* Ourebia ourebi *	Oribi	√			–	–	–
	* Raphicerus campestris *	Steenbok			√	2	0.4	0.1
	* Hippotragus equinus *	Roan	√			–	–	–
	* Kobus leche *	Lechwe	√			–	–	–
	* Tragelaphus spekii *	Sitatunga	√			–	–	–
Unidentifiable	Unidentifiable	Unidentifiable		√		18	15.2	–

Common duikers and large-spotted genets were the most frequently photographed species in both camera surveys (Table 3). Interestingly, leopards were only recorded in the second camera survey and not the first, but a total of four per 100 days of sampling would likely yield a single leopard photographic event, higher than any other carnivore recorded in our study (Table 3). Other species which stood out during one or both of the camera surveys were vervet monkeys, side-striped jackals, bushpigs, silver-backed duiker and blue duiker. All other species, although detected during the course of the study, were recorded in less than one per 100 days of sampling (Table 3). Unfortunately, 15% of the photographic events in the first camera survey were unidentifiable due mostly to poor placement of cameras (Table 3).

## Discussion

### Surveys of Okavango source lakes

Based on both acoustic and trapping surveys, at least 46 species of small mammals occur in the upper Okavango catchment region, including several rare and endemic species. This diversity compares favourably with studies reviewing the diversity of terrestrial small mammals (Taylor et al. 2015) and bats (Schoeman et al. 2013; Taylor et al. 2013; Cooper-Bohannon 2016; Herkt et al. 2016) in African highlands generally. However, the estimate of terrestrial small mammal richness is probably grossly under-estimated as a GBIF search of central Angola yielded 42 species, compared with our list of 13 species of shrews and rodents based on captures. Many of the small mammals recorded in our survey such as shrews, Anchieta’s vlei rat and fruit bats are habitat specialists which would be adversely affected by deterioration of wetlands due to anthropogenic effects such as extensive fires, tree clearing, wetland drainage and overgrazing, typical in the Miombo woodlands (Syampungani et al. 2009; Jew et al. 2016). Fruit bats provide valuable ecosystem services through seed dispersal and pollination, Anchieta’s broad-faced fruit bat possesses whiskers which are thought to be involved in pollination (Monadjem et al. 2010). Bats of the genera *Eidolon*, *Epomophorus*, and *Rousettus* are known to pollinate baobab trees (*Adansoniadigitata*) over much of Africa (Baum 1995).

### Mammal checklist

**Afrosoricida**

This order is represented by two families in mainland Africa: Chrysochloridae (golden moles) and Potamogalidae (otter-shrews). Angola harbours just one species of golden mole, the Congo golden mole *Huetialeucorhina* (previously *Calcochlorisleucorhinus*, see Asher et al. 2010). This group of subterranean fossorial mammals is therefore either poorly represented in Angola, or it has been greatly overlooked in the country. The species *H.leucorhina* is classified as Data Deficient and is known from just 10 scattered locations including one from Angola where a series of seven specimens from the Field Museum of Natural History were collected by H. R. Heinrich in 1954–1955 from Canzele, 30 km west of Camabatela in central Angola. Otter-shrews are represented by just three species, all of them associated with tropical forests in Africa. Of these, the largest and most widely distributed *Potamogalevelox* has been recorded from numerous localities in forested regions of northern Angola.

**Carnivora**

In addition to 37 species of Angolan carnivores in the GBIF database, reliable literature records (Kingdon and Hoffmann 2013a) add three species, including the Congo clawless otter *Aonyxcongicus* and the African golden cat *Profelisauratus* from N Angola, and the black-footed cat *Felisnigripes* from extreme south-east Angola. The king genet *Genettapoensis*, the servaline genet *G.servalina*, the central African linsang *Poianarichardsonii*, the long-nosed mongoose *Xenogalenaso* and the black-legged mongoose *Bdeogalenigripes* all have distributions apparently encompassing or bordering Cabinda but without any specific mention of their occurrence in Cabinda, Angola (Kingdon and Hoffmann 2013a) so we do not add them to the checklist.

This order is represented by seven terrestrial families in Africa: Canidae (dogs), Mustelidae (weasels, polecats and allies), Nandiniidae (palm civet), Felidae (cats), Viverridae (genets and civets), Hyaenidae (hyenas and aardwolf) and Herpestidae (mongooses). Five canid species have been recorded in Angola. However, only two species (African wild dog *Lycaonpictus* and side-striped jackal *Canisadustus*) were photographed and/or sighted in the Okavango catchment during our 2016 assessment. Nevertheless, the relatively frequent rate with which the wild dogs were photographed on the camera traps (capture frequency = 0.6) is pleasing given their Red List Endangered status on the continent.

Eight felid species occur in Angola of which six (all except *P.auratus* and *F.nigripes*) were recorded during our 2016 camera trapping, suggesting that these species may have relatively cosmopolitan distributions across the country. However, our field data indicates that the largest of these obligate carnivores (the lion *Pantheraleo*) occurs at much lower densities than the other felids. The lion is often one of the first species to be lost from the large carnivore guild when prey becomes limiting and/or conflict with humans escalates (*sensu* the effects of war). Thus, their presence in the south-east of Angola (albeit patchy) suggests some post-civil war recolonization. Interestingly, leopards *P.pardus* were the most frequently encountered felid during the 2016 camera trapping (capture frequency = 3.6) supporting the notion that leopards are adaptable generalists.

Two hyena species and the aardwolf *Protelescristatus* have been recorded in Angola. However, only the aardwolf and the spotted hyena *Crocutacrocuta* were photographed in our 2016 assessment. The latter finding is somewhat unsurprising given that brown hyena *Parahyaenabrunnea* density is known to be negatively correlated with that of spotted hyenas (Mills 2015).

The Herpestidae family is presented by 12 Angolan species, with several of these species occurring throughout much of sub-Saharan Africa. Three species of the mongoose family were formally recorded in the Okavango catchment survey of 2016, the marsh mongoose, *Atilaxpaludinosus* was recorded from a visual and camera survey, with banded mongoose, *Mungosmungo* and white-tailed mongoose *Ichneumiaalbicaudata* just recorded by camera surveys. The banded mongoose occurs in a broad range of habitats, with extensive range throughout Angola (Gilchrist and Do Linh San 2016). Selous’s Mongoose, *Paracynictusselousi* occurs in savanna and woodland in central and southern parts of Angola (Stuart and Stuart 2013a). The black-legged mongoose, *Bdeogalenigripes* occurs in far north-west Angola (van Rompaey and Colyn 2013a). Yellow mongooses *Cynictispenicillata* occur marginally in southern Angola (Taylor 2013a). The highly social meerkat, *Suricatasuricatta*, is endemic to the more arid open regions of western parts of southern Africa, and has a marginal intrusion in to SW Angola (Macdonald 2013). The dwarf mongoose (*Helogaleparvula*, and a subspecies *H.parvulamimetra* (sometime treated as valid species *H.mimetra*) are reported to occur in Angola (Creel 2013), with *H.parvula* reported to occur in open woodlands, thickets and often associated with termite mounds etc., allowing for suitable dens (Creel 2013). Creel (2013) also indicates that there is a subspecies *H.p.varia* that occurs in north-east Angola.

Three species of *Herpestes* occur in Angola, the Egyptian mongoose, *H.ichneumon* (Do Linh San et al. 2016), the common slender mongoose *H.sanguineus* (Hoffmann and Taylor 2013), and the Kaokoveld slender mongoose, *H.flavescens* from SW Angola including Benguela Province (Taylor 2013c). The taxonomic history of *H.flavescens* is very confusing. It has been included as a chestnut-coloured race of different species included in either *H.pulverulentus* or *H.ochraceus* but these two last-mentioned species are now considered to have much more restricted distributions in South Africa and S Namibia and Somalia and Ethiopia respectively. Crawford-Cabral (1996) demonstrated that *flavescens* was conspecific with the blackish-coloured *nigrata* race from north-east Namibia, a view that we follow. A chestnut-coloured form from Caconda near Benguela in Angola, *H.ansorgei* Thomas & Schwann, 1905 should be assigned to *H.flavescens* on colour and geographic grounds, and not to *H.ochraceus* as proposed by Taylor (2013b).

Ansorge’s Crusimanse *Crossarchusansorgei* is a cryptic Angolan Herpestidae species known from a single location from the proposed Angolan distribution collected in 1908 (Angelici and Do Lin San 2015 citing Crawford-Cabral 1989; Van Rompaey and Colyn 2013b).

*Mustelidae*. The Zorilla, *Ictonyxstriatus*, ranges across a broad range of habitats of sub-Saharan Africa, and is common throughout its range, yet reported to be locally uncommon (Stuart and Stuart 2013b). Our upper Okavango catchment study of 2016 recorded this species by visual sightings and camera trap surveys. African clawless otters, *Aonyxcapensis*, are medium sized otter species, and occur widely, associated with seasonal rivers, and can occur in fresh and marine water. The clawless otter is reported to have a distribution range in the central and southern parts of Angola (Somers and Nel 2013), yet was not recorded in the 2016 assessment. The spotted necked otter, *Hydrictismaculicollis* appears to be closely linked to permanent freshwater systems with abundant food resources (fish) and shoreline cover. Their continental distribution is considered to be wide. However, there is a paucity of information for this otter species in Angola (Reed-Smith et al. 2015). The ratel, *Mellivoracapensis*, occurs across Africa in most habitat types, from deserts to forests. This species is reported to exist in low densities, and is often regarded to be rare throughout its range (Begg et al. 2013). This species was recorded in the Okavango catchment expedition through visual observation and camera trap survey. African striped weasels, *Poecilogalealbinucha*, are thought to be uncommon and rare, throughout their extensive range in sub-Saharan Africa (Stuart and Stuart 2013c), and are delineated to occur in the northern parts of Angola.

*Nandiniidae*. The two-spotted Palm Civet, *Nandiniabinotata*, are arboreal and found across the west and central African forest belt, likely to only be found in the North east of Angola (Gaubert 2013).

*Viverridae*. The African civet, *Civettictiscivetta*, is distributed throughout sub-Saharan Africa, with suitable habitat from 29°S latitude extending north to south of 15°N latitude, and a commonly encountered carnivore (Ray 2013). This species was recorded from visual observation and through camera trap surveys in our upper Okavango catchment expedition. The Miombo genet, *Genettaangolensis*, originally known from three adult specimens, lost in a fire in 1978 (Crawford-Cabral 2013) has a broad distribution from Angola, Democratic Republic of Congo, Mozambique, Malawi, Zambia and Tanzania. This species is reported to be similar in size to the common genet, and occurs in “open miombo woodlands” and is reported to be locally common in some areas (e.g., the Luando Strict Nature Reserve (Central Angola) (Crawford-Cabral 2013, Gaubert et al. 2016). The common genet, *Genettagenetta*, is a widespread and locally common genet, and in its southern distribution range occur from central and south Angola through Botswana, Namibia, Zimbabwe and South Africa, replaced to the east by the large spotted genet (Delibes and Gaubert 2013). *Genettamaculata*, along with *G.tigrina* and *G.pardina* form part of the ‘large spotted genet complex’ (Gaubert and Dunham 2013). The species is very common and widespread with over 50 localities recorded by GBIF records. Based on camera trap images and visual sightings, *G.maculata* was present during the Okavango catchment expeditions in 2016. The servaline genet *G.servalina* is depicted as occurring in Cabinda by Van Rompaey and Colyn (2013c) but without any specimen or sighting records, its occurrence there is dubious albeit possible.

**Certartiodactyla**

This order is represented by five indigenous families in Africa and Angola: Suidae (pigs), Hippopotimidae (hippopotamuses), Tragulidae (chevrotains), Giraffidae (giraffes) and Bovidae (bovines and antelopes). Following the taxonomy of Kingdon and Hoffman (2013b), a total of 32 artiodactlys are recognised in Angola, of which 26 are bovids (Suppl. material 1). Following the taxonomy of Groves and Grubb (2011), the list would result in 35 Angolan Artiodactyla including 29 bovids. This increase of three bovid species is because these latter authors regard Lichtensteini’s harbertebeest *Alcelaphuslichtensteini* as a separate species from the red hartebeest *A.buselaphus*; furthermore, two species (a tropical forest Kabinda species and a southern savanna species) are recognised each within the African buffalo (*Syncerusnanus* and *S.caffer* respectively) and the bushbuck (*Tragelaphusornatus* and *T.phaleratus* respectively) populations from Angola. The taxonomy of Groves and Grubb (2011) doubled the number of recognised African ungulate species. This species-splitting process would raise several local Angolan subspecies to species rank and result in name changes for several of the Angolan ungulates. It would make two bovid species endemic to Angola, the Angolan blue duiker *Philantombaanchietae* and the Angolan springbok *Antidorcasangolensis*. The klipsringer *Oreotragustyleri* would become near-endemic to Angola, extending marginally to north-west Namibia. Some conservationists have opposed the taxonomy of Groves and Grubb (2011); for recent debates see: Gippoliti and Groves (2012), Heller et al. (2013), Zachos et al. (2013), Cotterill et al. (2014), Heller et al. (2014), Garnett and Christidis (2017), Cotterill et al. (2017), and Groves et al. (2017).

**Figure 3.**
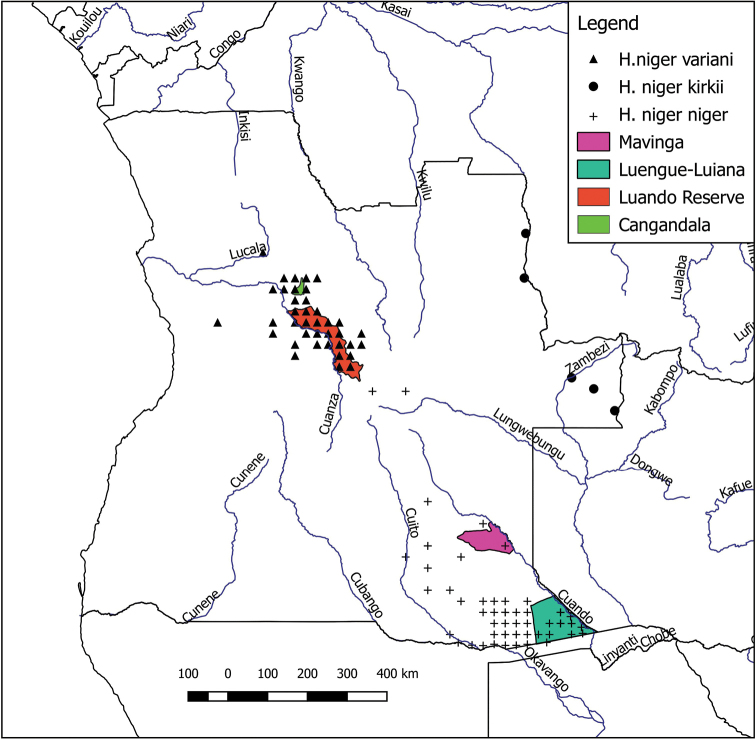
Historical distribution of the nominate sable subspecies (*Hippotragusnigerniger*), Kirk’s sable (*H.nigerkirkii*) and the giant sable (*H.nigervariani*) in Angola based on records of the Instituto de Investigação Científica Tropical (IICT) in Portugal (obtained via GBIF search). Protected areas where *H.n.niger* and *H.n.variani* currently known to occur are indicated in the legend. The subspecies *H.n.kirkii* from eastern Angola has not been recorded during the past 40 years.

GBIF yielded 5,821 species-identified historical records of Artiodactyla dating back to 1889, of which 5,438 were compiled by the IICT in Portugal, and 385 were preserved specimens, predominantly from the American Museum of Natural History and Field Museum of Natural History. These records provide valuable insights into the historical distribution of the larger ungulates, some of which have become extinct or almost extinct in the wild in Angola. For example, the GBIF database provides 13 georeferenced records of Lichtenstein’s hartebeest from 13 localities throughout E Angola, whereas this species is now thought to be extinct in Angola (Gosling and Capellini 2013). The GBIF database contains 213 records (including 25 museum specimens dating back to 1925) of the black-faced impala (*Aepycerosmelampuspetersi*) from 78 georeferenced localities throughout S Angola whereas the status of the species in Angola is currently uncertain. It occurs at Iona National Park and possibly two other national parks (Fritz and Bourgarel 2013). The case of the three sable subspecies, the nominate form (*Hippotragusnigerniger*), Kirk’s sable (*H.nigerkirkii*) and the giant sable *H.n.variani* is also remarkable. All three subspecies were widespread in central, south-eastern, and eastern Angola (Figure 3) but are currently much more restricted. The giant sable came close to extinction during the Angola civil war and is currently known from just two reserves in central Angola, the Cangandalo National Park and the Luando Reserve (Figure 3) where its survival continues to be threatened by poaching and hybridization with roan antelope *Hippotragusequinus* (Vaz Pinto 2006, 2018; Estes 2013). In the case of Kirk’s sable from eastern Angola, (Figure 3), there have been no observations for 40 years (Cabral and Veríssimo 2005). However the nominate race (*H.n.niger*) is still known to occur in the region between the Cuito and Cuando Rivers in southeastern Angola, including the Luengue-Luiana and Mavinga National Parks (Funston et al. 2017, Figure 3). However no sable were detected in the present camera trap survey or ad hoc observations conducted in the upper Okavango catchment.

A dubious record of the common reedbuck *Reduncaredunca* from Calunga in SE Angola in the Los Angeles County Museum must be a misidentified *R.arundinum* since *R.redunca* is not known to occur anywhere near Angola (Kingdon and Hoffman 2013b).

As one of the most speciose certartiodactyl families in Africa, it is unsurprising that 26 bovid species occur in Angola. Only eight of these species were recorded during our 2016 survey in the south-east (*Cephalophussilvicultor*, *Philantombamonticola*, *Sylvicapragrimmia*, *Ourebiaourebi, Raphiceruscampestris*, *Hippotragusequinus*, *Kobusleche* and *Tragelaphusspekii*). Anecdotal data from our camera trapping and discussions with local Angolans suggests that the bovids are likely the most targeted mammalian group when it comes to poaching for bushmeat that is routinely traded commercially in many parts of the country.

Three pig species have been recorded in Angola. The common warthog *Phacochoerusafricanus* and the bushpig *Potomochoeruslarvatus* have been collected/sighted from numerous localities across Angola. However, the red river hog *Potomochoerusporcus* is restricted to the forested northern regions of the country based on 12 GBIF records (seven museum specimens and five additional historical records from IICT). Although not formally recorded during our 2016 camera trapping survey, hippopotamuses *Hippopotamusamphibius* have been regularly recorded across Angola wherever suitable water and nocturnal grazing is available. Only one chevrotain species *Hyemoschusaquaticus* has been recorded in Angola and this species occurs in the forested north of the country. The giraffe *Giraffacamelopardalis* has been recorded at numerous localities in Angola but was not formally recorded in the upper Okavango catchment during our 2016 assessment. However, what may have been giraffe tracks/spoor were noted.

**Chiroptera**

The GBIF search alone reported 59 species of Angolan bats while Monadjem et al. (2010a) reported 64, seven of which were not included in the GBIF search. The current survey added one new Angolan species record based on captures (*Pipistrellusrusticus*) and three tentative species records based on acoustic identification (*Kerivoulaargentata*, *Mopsmidas*, *Pipistrellushesperidus*; Table 2). Happold and Happold (2013) added one species (*Triaenopsafer*). Merging these three sources resulted in a combined list of 71 species (Suppl. material 1). Over 220 species of bats have been recorded in mainland Africa of which over 75% occur in Sub-Saharan Africa (Happold and Happold 2013). Of these, 116 species occur in southern Africa defined by Monadjem et al. (2010a), a region which includes Angola. Within this region, the more diverse countries have bat species richness typically between 60–70 species (Monadjem et al. 2010b). For example, 67 species have been recorded in Mozambique, 65 species in Zambia, and 62 species each in Malawi and Zimbabwe (Monadjem et al. 2010b). Therefore, the current total of 71 species that we report for Angola in this study, is on the higher end of richness for the region, in spite of the fact that Angola remains one of the most poorly known southern African countries with respect to bats (and mammals in general). For example, during the course of just a few weeks of fieldwork we were able to add a new species of bat to the country list, as well as additional (possible) new records of a further three species, based on echolocation calls, which would bring the total up to 71 species (making it the most diverse country in the region). We suspect that there are numerous species of bat that still await discovery in Angola, and we further predict that a number of new (and possibly endemic) species will be eventually recorded from the western escarpment region that is high in bird endemism (Mills et al. 2011, 2013). To the best of our knowledge, no recent bat (or other small mammal) surveys have been conducted in any of the remaining Afromontane forests and adjacent grasslands along this escarpment, and this must remain a critical zone for future surveys.

Two other regions are likely to be fruitful survey locations for bats. One is Kabinda which is on the northern side of the Congo River; this river forms a substantial barrier for African mammals resulting in it forming the southern limit for many species. To the best of our knowledge, Cabinda has not been surveyed specifically for bats before. We suspect that a large number of tropical rainforest species (e.g., *Myopteruswhitleyi*, *Mopsannulus*, *Mopsthersites* and *Nycterisgrandis*) will be shown to occur in Cabinda. The second is the extreme south-western arid zone on the Namibian border (part of which is included in Iona National Park). This region has also not been the focus of dedicated bat surveys, but may harbour arid zone specialist species (e.g., *Sauromyspetrophilus* and *Rhinolophusdenti*) that have not yet been recorded from Angola. We suspect that these three regions (western escarpment, south-western arid zone and Kabinda) will also harbour new country records of other mammal groups, and therefore encourage mammalogists to specifically target these areas.

One reason for this high bat diversity is the considerable range in habitats from lowland desert to high altitude Afromontane forest, and from open grassland to tropical rainforest. Despite the high diversity, no bat species are endemic to Angola. This is rather surprising considering the large number of endemic birds and reptiles (Mills et al. 2011). We suspect that future research will uncover endemic bat species to Angola, most probably along the western escarpment, and particularly on isolated mountain tops coated in forest. By way of example, *Rhinolophuseloquens* has been collected from Jau, Huila Province, a location that is over 2,000 km away from the closest records in the east of the Democratic Republic of Congo (DRC) and Rwanda. These specimens from the American Museum of Natural History are worth re-examining as we suspect that this may refer to a new species within the *R.eloquens*/*R.hildebrandtii* group.

**Hyracoidea**

Four species of hyraxes are confirmed in Angola including two species of tree hyraxes (Kingdon et al. 2013a). A large number of records of the southern tree hyrax *D.arboreus* in the GBIF database come from 36 georeferenced localities in north-east Angola. The western tree hyrax *D.dorsalis* is known only from Cabinda in Angola (Shulz and Roberts 2013) and is vouched for by two specimens from the collection of the Royal Belgian Institute of Natural Sciences.

**Lagomorpha**

Only one lagomorph species (*Lepusvictoriae*, *African savanna hare*) is known from Angola. GBIF records assigned to *L.capensis*, *L.saxatilis* and *Poelagusmarjorita* must be mis-identifications as there is no valid modern records or voucher specimens for the occurrence of these species in Angola.

**Macroscelididea**

Only two species of sengis or elephant shrews are known to occur in Angola, *Elephantulusbrachyrhynchus* and *E.intufi* (Suppl. material 1). Based on proximity to the border, *Macroscelidesproboscideus* and *Elephantulusrupestris* may eventually be shown to occur in south-west Angola.

**Erinaceomorpha**

Only one widespread species in known, the southern African hedgehog, *Atelerixfrontalis*.

**Perissodactyla**

Three native species can be confirmed for Angola (Suppl. material 1). This order is represented by two families in Africa: Equidae (zebras) and Rhinocerotidae (rhinoceroses). Two zebra species can be confirmed to occur in Angola. The threatened Hartman’s zebra *Equuszebrahartmannae* is known from Iona National Park while the plain’s zebra *E.quagga* is more widely distributed across the country. A single Grevy’s zebra *E.grevyi* specimen housed by UCM is from an unknown collector and the locality is simply given as Angola and is likely misidentified since the species is known only from Kenya and Ethiopia (Williams 2013). Only the black rhinoceros *Dicerosbicornis* has been recorded in Angola and they have all but been extirpated from the country due to the civil war. No zebras or rhinoceroses were recorded during our 2016 camera trapping assessment.

**Pholidota**

Four species of pangolin are present in Africa, of which two have been confirmed within the borders of Angola (the ground pangolin *Smutsiatemmincki* and the tree pangolin *Phataginustricuspid* (Hill and Carter 1941; Kingdon and Hoffman 2013b) and two (*Smutisagigantea* and *Phataginustetradactyla*) may possibly occur in Cabinda (the Congo River is the southern limit for both these species); this makes Angola possibly the only country on the continent where all pangolin species probably occur.

**Primates**

After correcting for taxonomic changes, the GBIF database listed 17 species of primates occurring in Angola. Based on species distributions in Butynski et al. (2013), two of these GBIF records could not be verified. Although three un-dated specimens from the Royal Belgian Institute of Natural Sciences from Angola (no specific locality) were labelled as the grey-cheeked mangabey *Lophocebusalbigena*, according to Butynski et al. (2013) the species occurs only north of Cabinda. While it could conceivably occur in Cabinda, until we have clear evidence, we regard the Angolan GBIF records of *L.aligena* as dubious. Likewise, although the Red-capped Mangabey *Cercocebustorquatus* is not documented as occurring in Angola by Ehardt (2013), two undated GBIF specimens from Cabinda in the Royal Belgian Institute of Natural Sciences collected by Serge M. Frechkop are labelled as *C.torquata*. Although the species is known to occur near to the Cabinda border, for the moment we treat this record as uncertain. Although the Kinda baboon *Papiokindae* from Angola was included as a synonym of the yellow baboon *P.cyanocephalus* by Butynski et al. (2013), it was shown to be distinct by Mittermeier et al. (2013). Therefore *P.kindae* and not *P.cyanocephalus* is found in Angola. One species not included in the GBIF database but validated by Goossens et al. (2002) and Butynski et al. (2013) as occurring in Cabinda, is the robust chimpanzee *Pantroglodytes*. Considering the above, we consider there to be 16 species of primates occurring in Angola.

Cercopithecidae. The red-tailed monkey *Chlorocebusascanius* (synonym *Cercopithecusascanius*) occurs through most of central and north Angola into DRC and other countries. With a highly fragmented distribution, three subspecies are considered to occur in parts of Angola, *C.a.katangae*, *C.a.atrinasus*, and *C.a.ascanius* (Oates et al. 2008a). *Chlorocebusascaniusatrinasus* is reportedly found in northwest Lunda district of northeast Angola, and known from nine specimens (Oates et al. 2008b). GBIF records account for one 1925 Angolan specimen of *C.ascanius* in the American Museum of Natural History collected by Lang, and four specimens from the Field Museum collected in 1954 by G. H. Heinrich from Canzele, 30 km west of Camabatela. The range of the moustached monkey *Cercopithecuscephus* extends from Cameroon southwards into parts of far north west of Angola (likely Cabinda province) (Oates et al. 2008c). The gentle monkey, or blue monkey *Cercopithecusmitis* has great variation with disputed taxonomy but the nominate subspecies *C.m.mitis* is generally considered to be endemic to Angola (Lawes et al. 2013). The De Brazza’s Guenon *Cercopithecusneglectus* is found in NE Angola associated with riverine forest habitats, common in many parts of its range (Struhsaker et al. 2008). The Malbrouck monkey *Chlorocebuscynosuros* is part of the *C.aethiops* (vervet) group but was elevated to full species by Groves (2001). *Chlorocebuscynosuros* extends throughout Angola, Zambia and parts of the DRC (Sarmiento 2013). A total of 25 GBIF records from six widely separated localities in west and central Angola document its widespread presence within the country.

The Angola colobus, *Colobusangolensis*, is considered to be endemic to equatorial Africa. The Sclater’s Angola colobus, *C.a.angolensis* occur throughout the north-east part of Angola, with populations restricted to forest fragments (Bocian and Anderson 2013).

The southern Talapoin monkey *Miopithecustalapoin*, or Angolan Talapoin monkey is regarded as endemic to Northern Angola and south-west DRC.

The Kinda baboon occurs in central and northern parts of Angola (north of the Cunene river) ranging eastwards through DRC, Zambia. The preferred habitat of this species is reported to be Miombo (*Brachystegia*) woodland in the fire-climax stage (Kingdon 2016). The Chacma baboon *Papioursinus*, is sympatric in Angola in some part of the range of *Papiokindae*, with the Chacma considered endemic to southern Africa. The species is considered to have three subspecies, of which two inhabits Angola: *P.u.ruacana* ranging into south and central Angola from its Namibian distribution and the *P.u.griseipes* ranging through Zimbabwe, Botswana, and Mozambique with a south eastern intrusion to Angola (Cowlishaw 2013). The chacma baboon occurs in a broad range of habitats within Angola (Hoffman and Hilton-Taylor 2008).

The southern lesser galago *Galagomoholi*, ranges broadly across southern Africa, Angola in the North-western extension of their range, reportedly being from the subspecies *G.m.bradfieldi* that occur in north Namibia and south central Angola through to southern DRC and then Zambia and Tanzania, south into northern South Africa (likely the *G.m.moholi*). The southern lesser galago prefers semi-arid woodland and savanna habitats (Pullen and Bearder 2013). The Demidoff’s Dwarf Galago, *Galagoidesdemidoff*, also referred to *G.demidovii*, are recorded in the northern parts of Angola extending northwards and north-eastwards into the DRC Rainforest and Afromontane–Afroalpine forests zones (Bearder 2016). The large eared greater Galago, *Otolemurcrassicaudatus*, (synonym *Galagocrassicaudatus*) is found throughout most of southern Africa, ranging from Angola in the west, to Tanzania in the east, extend south into KwaZulu-Natal, South Africa. The proposed subspecies, the Miombo silver Galago *Otolemurc.monteiri* (or often elevated to species level *O.monteiri*) are found widely across the range in Angola (Bearder and Svoboda 2013). We photographed what we believe was *Otolemurc.monteiri* during one of the camera trap surveys in the Okavango catchment expedition in 2016.

The western Gorilla *Gorillagorilla*, extends only into the Cabinda province of Angola.

The potto, *Perodicticuspotto*, is considered to have several subspecies and even groups that can be elevated to species designation. The potto, *P.pottoedwardsi* occurs in the north-east of Angola and in Cabina, occurring in a range of habitats (Pimley and Bearder 2013). Two undated specimens from Cabinda are located in the Royal Museum of Central Africa.

**Proboscidea**

There are two elephant species in Africa, *Loxodontaafricana* and *L.cyclotis* and both occur in Angola (Kingdon et al. 2013a). Despite being the largest, and presumably most conspicuous mammal, records of elephant presence in Angola have declined substantially post-civil war. Their current distribution appears to be restricted to the northern parts of the country. However, elephant signs (dung and tracks but not photographs) were recorded during our 2016 survey in the Okavango catchment.

**Rodentia**

The 77 Angolan rodent species identified by the GBIF search provided a close match to the 78 Angolan rodent species recorded by Monadjem et al. (2015). Of the 77 species recorded by GBIF, four species were deleted from the final checklist as they could not be validated due to imprecise locality information and/or un-corroborated identifications which were not supported by other studies: the squirrel, *Heliosciurusrufobrachium*, the molerat *Fukomysdamarensis*, the cane rat *Thryonomysgregorianus* and the climbing mouse, *Dendromusmessorius*. An additional seven species were added based on validated species records from Happold (2013) or Monadjem et al. (2015): the squirrels, *Funisciuruslemniscatus* and *Anomalurusbeecrofti* (Happold 2013), the dassie rat *Petromustypicus*, the long-eared mouse *Malacothrixtypica*, the striped mouse *Rhabdomysbechuanae*, the giant rat *Cricetomysemini*, and the climbing mouse *D.leucostomus* (Monadjem et al. 2015). Two new species were added by the current survey of the Angolan Highlands: Setzer’s mouse *Mussetzeri* and Woosnam’s broad-headed mouse *Zelotomyswoosnami*. This brings the final total of Angolan rodent species to 82. As indicated below, several additional rodent species are known to occur on or close to the borders of Angola and will probably be shown to occur there in the future.

Sciuridae. Thomas’s rope squirrel *Funisciurusanerythrus* has been recorded at the border of Kabinda and could possibly occur there (Monadjem et al. 2015). The ribboned rope squirrel *F.lemniscatus* was recorded from Kabinda by Happold (2013) although not reported there by Monadjem et al. (2015). The red-legged sun squirrel *Heliosciurusrufobrachium* is not known to occur south of the Congo River but a GBIF record from “Raca Camele, north of Quionlungo” was attributed to a specimen from Yale Peabody Museum identified by A. Heinrich. We flagged this as a dubious record. The isolated population of the forest giant squirrel *Protoxerusstangeri* in northern Angola is treated as an endemic subspecies *P.s.loandae* (Happold 2013).

Gliridae. A high diversity of five species of dormice has been recorded in Angola.

Muridae. Muridae comprises by far the largest family of rodents and 42 indigenous murid species occur in Angola. The species list from the GBIF database corresponded closely with that of Monadjem et al. (2015). Our current trapping survey of the Okavango source lakes region added two murid rodent species to the list for Angola, *Mussetzeri* and *Zelotomyswoosnami* (Table 1). As both of these rodents are associated with sandy soils in arid savannas it was surprising to record them from mesic miombo woodlands in the Angolan highlands. However, the rivers and source lakes of the upper Okavango catchment are bordered by sandy habitats that form obvious corridors for the dispersal of these species from the lower catchments of the Okavango Basin where they have hitherto been recorded in Botswana and Namibia. Given that two murid species were added to the country after just two weeks of survey effort in a limited area, it is highly likely that more species of murid rodents will be shown to occur in Angola by future collecting. For example tropical species such as *Deomysferrugineus*, *Lophuromysansorgei*, *Hybomysunivitattus*, *Hylomyscusaeta*, *H.anselli*, *Mylomysdybowski*, *Praomyspetteri* and *Stochomyslongicaudatus* occur close to the border of Kabinda or north Angola. On the other hand, species from arid savannas associated with the Okavanga River in Botswana and Namibia, such as *Dasymyscabrali*, may also be found to occur in the upper Okavanga catchments. Although the desert pygmy mouse *Musindutus* was shown to occur in south-east Angola by Happold (2013), identification of this species is difficult and no molecular sequences are available of this species from Angola (Lamb et al. 2014, Monadjem et al. 2015). Hence, this species is included in the checklist as a dubious record. Another semi-arid habitat murid species that extends into extreme south-west Angola is *Rhabdomysbechuanae* (Du Toit et al. 2012).

Nesomyidae. The presence of Emin’s giant pouched rat *Cricetomysemini* from Kabinda is vouched by Musser and Carleton (2005) and Monadjem et al. (2015) but the species does not appear to occur as widely in north Angola as indicated in Happold (2013). The banana African climbing mouse *Dendromusmessorius* was recorded by three specimens from the Field Museum from Dundo in the extreme north-east Angola collected by A. Barros Machado in 1948, but there are no known records close to this (Monadjem et al. 2015). We suspect this is a misidentification, and it is interesting that Hayman (1963) commented on a series of five *Dendromus* from the Dundo Museum as follows: “This unstriped *Dendromys* of the Lubda District appears to represent *ansorgei* rather than *messorius* Thomas of the Cameroons, under which name Sanborn (1952) listed a series from the Dundo region examined by him”. Since *ansorgei* is a synonym of *mystacalis*, it seems very likely that specimens from Dundo refer to the chestnut African climbing mouse *D.mystacalis*. Vernay’s African climbing mouse *D.vernayi* is endemic to the central highlands of Angola and only known from the type locality. The monotypic *Dendroprionomys* (velvet climbing mouse) is known only from the type locality Brazzaville which is close to Cabinda and may be shown to occur there. The long-eared mouse *Malacothrixtypica* was not recorded in the GBIF database but is known from extreme south-west Angola (Hill and Carter 1941; De Graaff 1981).

Hystricidae. African brush-tailed porcupines *Atherurusafricanus* have been recorded on the border region of Cabinda on both Congo and DRC sides, and undoubtedly this species occurs in Cabinda (Monadjem et al. 2015); however without material evidence we do not include it here. The common porcupine *Hystrixafricaeaustralis* has been recorded throughout the country.

Petromuridae. Although not recorded in GBIF, several records of the dassie rat *Petromustypicus* are known from south-west Angola (Monadjem et al. 2015).

Thryonomyidae. The GBIF database contains records from three specimens labelled as *Thryonomysgregorianus* from mount Moco collected in 1954, but the closest known records of this species are from central DRC and west Zambia (Happold 2013; Monadjem et al. 2015). Given the difficulty in distinguishing this species from *T.swinderianus*, we treat this record as dubious.

Pedetidae. Only one widespread species is known to occur in Angola.

Anomaluridae. Although not listed in the GBIF database, both Happold (2013) and Monadjem et al. (2015) show Beecroft’s scaly-tailed squirrel *Anomalurusbeecrofti* occurring in Kabinda. Happold (2013) also indicates additional records from Angola south of the Congo River.

**Soricomorpha**

Our GBIF search revealed 13 species of shrews occurring in Angola, all from the genus *Crocidura*, but cross-checking against Hill and Carter (1943) and Happold and Happold (2013) resulted in two species being flagged as highly dubious, plus an additional two species known to occur in Angola (Hill and Carter 1943; Happold and Happold 2013), resulting in a final total of 13 species.

The lesser grey-brown shrew *C.silacea* and Dent’s shrew *C.denti* are both included in the GBIF database but their known range is nowhere near Angola (Happold and Happold 2013). The *C.denti* record was an undated record from the Natural History Museum with no recorded locality. The *C.silacea* record was from the Field Museum of Natural History, collected in 1954 from near Quela in north Angola by G. H. Heinrich. Given its location many hundreds of kilometers from other known records, and difficulties in identification of this species, we believe this to be a misidentification and this record is regarded as highly dubious. The heather shrew *C.erica* and the blackish shrew *C.nigricans* are both endemic to Angola. Ansell’s shrew *C.ansellorum* and the moonshine shrew *C.luna* have both been collected from the border region of Zambia and Angola, and probably occur in Angola.

Both the greater dwarf shrew *Suncuslixus* and the climbing dwarf shrew *Suncusmegalura* are known to occur in Angola (Happold and Happold 2013), hence were added to the final checklist (but were not captured in the GBIF database). Our field collections in the Okavango source lakes area in 2016 added an additional species for the country, the lesser dwarf shrew *Suncusvarilla* (Table 1). The species has a sparse distribution and was previously known from sout-east DRC so its occurrence in central Angola is not surprising. Although Hill and Carter (1943) described a similar number of species (13) as here recognised (14), some of their names have become synonyms and their list corresponded to nine of the currently recognised species, representing a 44% increase in real diversity since 1943.

### Tubulidentata

The aardvark *Orycteropusafer*, is considered to be a common species in suitable habitats across its range which extends through most of sub-Saharan Africa (Taylor and Lehmann 2015), and is strongly associated with ant nests and termitaria. This species was recorded in the Okavango catchment expedition during our camera trap survey in 2016, and probably occurs widely in the country.

## Concluding remarks

The current list of 275 species of Angolan mammals represents an increase of 52 species compared to the exhaustive survey of Hill and Carter (1941) that recorded 223 species. Throughout most of the twentieth century, Angola was largely neglected in terms of mammal research, particularly during the period of the civil war in the late twentieth century (1975–2002), but recent decades have seen renewed research efforts. For example, through the National Geographic Okavango Wilderness Project, acoustic, camera trap and trapping surveys carried out between 2015 and 2017 in the upper Okavango catchment, a previously neglected area, has added valuable new data on mammal occurrences. While estimates of bat species richness for the upper Okavango catchment based on combined acoustic and capture data from our study (33 species) correspond closely with predicted bat diversity for the region based on modelling studies (e.g., Herkt et al. 2016), our survey under-estimated non-volant small mammals species richness (13 species compared to 42 species predicted based on GBIF records for the central Angola plateau).

Rodrigues et al. (2015) identified four biogeographical subdivisions in Angola based mostly on ungulate distributions. In the north, the Zaire-Lunda-Cuanza region was mainly associated with Congolian forests. In the south, the Namibe and Cunene-Cuando Cubango regions were mainly characterized by ungulates widespread in south-western and southern Africa. In between these regions, the Central Plateau region was mainly characterized by a few widespread ungulate species that are relatively common in dense miombo woodlands. These patterns were largely determined by a north-south gradient of decreasing humidity, from mesic tropical forests in the north to savannas and then more arid regions in the south. Patterns corresponding to this rainfall gradient are also evident in bats (Monadjem et al. 2010) and rodents (Monadjem et al. 2015). Angolan is known for a fairly high number of endemic or near-endemic species, particularly in the case of rodents and shrews, where our study identified one shrew and six rodents endemic to the central and western Angolan highlands. The western escarpment Afromontane forests of Angola are particularly important as a centre of both species richness and endemism of plants and vertebrates including mammals (Carleton et al. 2015). We suggest that future work should target more intensive surveys of small mammals in the central and western Angolan highlands to verify the presence and conservation status of threatened and/or endemic small mammal taxa.

## Supplementary materials

Supplementary material 1 Test list of species names Data type: species data This is an open access article distributed under the terms of the Creative Commons Attribution License (CC BY 4.0), which permits unrestricted use, distribution, and reproduction in any medium, provided the original author and source are credited.Peter J. Taylor, Götz Neef, Mark Keith, Sina Weier, Ara Monadjem, Daniel M. Parker

Supplementary material 2 Labeled images of example sonograms of each bat species identified Data type: species data This is an open access article distributed under the terms of the Creative Commons Attribution License (CC BY 4.0), which permits unrestricted use, distribution, and reproduction in any medium, provided the original author and source are credited.Peter J. Taylor, Götz Neef, Mark Keith, Sina Weier, Ara Monadjem, Daniel M. Parker

Supplementary material 3 Excel spreadsheet of occurrence data from cleaned GBIG search of Angolan mammal Data type: occurence This is an open access article distributed under the terms of the Creative Commons Attribution License (CC BY 4.0), which permits unrestricted use, distribution, and reproduction in any medium, provided the original author and source are credited.Peter J. Taylor, Götz Neef, Mark Keith, Sina Weier, Ara Monadjem, Daniel M. Parker
