# Supplementary material for: Tapping into technology and the biodiversity informatics revolution: updated terrestrial mammal list of Angola, with new records from the Okavango Basin
Source: Zookeys. 2018 Aug 2;(779):51–88. doi: 10.3897/zookeys.779.25964 (PMC6085403; doi:10.3897/zookeys.779.25964)
Supplement: Supplementary material 1 — Test list of species names [file zookeys-779-051-s001.docx]

Supplementary File Appendix 1. Checklist of Angolan terrestrial mammals from GBIF and additional sources, listed by order and family. Additional species records contributed by Monadjem et al. (2010, 2015), Kingdon et al. (2013a) and the current captures and acoustic surveys are indicated in bold. Dubious records are indicated by question mark (see text for details).

| **Afrosoricida** |
| --- |
| **Chrysochloridae** |
| *Huetia leucorhina* (Huet, 1885) |
| **Tenrecidae** |
| *Potamogale velox* (Du Chaillu, 1860) |
| **Cetartiodactyla** |
| **Bovidae** |
| *Aepyceros melampus* (Lichtenstein, 1812) |
| *Alcelaphus buselaphus* (Pallas, 1766) |
| *Antidorcas marsupialis* (Zimmermann, 1780) |
| *Cephalophus dorsalis* Gray, 1846 |
| *Cephalophus nigrifrons* Gray, 1871 |
| *Cephalophus silvicultor* (Afzelius, 1815) |
| *Connochaetes taurinus* (Burchell, 1823) |
| *Damaliscus lunatus* (Burchell, 1823) |
| *Hippotragus equinus* (Desmarest, 1804) |
| *Hippotragus niger* (Harris, 1838) |
| *Kobus ellipsiprymnus* (Ogilby, 1833) |
| *Kobus leche* Gray, 1850 |
| *Kobus vardonii* (Livingstone, 1857) |
| *Madoqua kirkii* (Günther, 1880) |
| *Oreotragus oreotragus* (Zimmermann, 1783) |
| *Oryx gazella* (Linnaeus, 1758) |
| *Ourebia ourebi* (Zimmermann, 1783) |
| *Philantomba monticola* (Thunberg, 1789) |
| *Raphicerus campestris* (Thunberg, 1811) |
| *Redunca arundinum* (Boddaert, 1785) |
| *Sylvicapra grimmia* (Linnaeus, 1758) |
| *Syncerus caffer* (Sparrman, 1779) |
| *Tragelaphus oryx* Pallas, 1766 |
| *Tragelaphus scriptus* (Pallas, 1766) |
| *Tragelaphus spekii* Speke, 1863 |
| *Tragelaphus strepsiceros* (Pallas, 1766) |
| **Giraffidae** |
| *Giraffa camelopardalis* (Linnaeus, 1758) |
| **Hippopotamidae** |
| *Hippopotamus amphibius* Linnaeus, 1758 |
| **Suidae** |
| *Phacochoerus africanus* (Gmelin, 1788) |
| *Potamochoerus larvatus* (F. Cuvier, 1822) |
| *Potamochoerus porcus* (Linnaeus, 1758) |
| **Tragulidae** |
| *Hyemoschus aquaticus* (Ogilby, 1841) |
| **Carnivora** |
| **Canidae** |
| *Canis adustus* Sundevall, 1847 |
| *Canis mesomelas* Schreber, 1775 |
| *Lycaon pictus* (Temminck, 1820) |
| *Otocyon megalotis* (Desmarest, 1822) |
| *Vulpes chama* (A. Smith, 1833) |
| **Felidae** |
| *Acinonyx jubatus* (Schreber, 1775) |
| *Caracal caracal* Schreber, 1776 |
| ***Felis nigripes*** Burchell, 1824 |
| *Felis silvestris* Schreber, 1777 |
| *Leptailurus serval* Schreber, 1776 |
| *Panthera leo* (Linnaeus, 1758) |
| *Panthera pardus* (Linnaeus, 1758) |
| ***Profelis aurata*** (Temminck, 1827) |
| **Herpestidae** |
| *Atilax paludinosus* (G. Baron Cuvier, 1829) |
| *Bdeogale nigripes* Pucheran, 1855 |
| *Crossarchus ansorgei* Thomas, 1910 |
| *Cynictis penicillata* (G. Baron Cuvier, 1829) |
| *Helogale parvula* (Sundevall, 1847) |
| *Herpestes flavescens* Bocage 1889 |
| *Herpestes ichneumon* (Linnaeus, 1758) |
| *Herpestes sanguineus* (Rüppell, 1835) |
| *Ichneumia albicauda* (G. Baron Cuvier, 1829) |
| *Mungos mungo* (Gmelin, 1788) |
| *Paracynictis selousi* (de Winton, 1896) |
| *Suricata suricatta* (Schreber, 1776) |
| **Hyaenidae** |
| *Crocuta crocuta* (Erxleben, 1777) |
| *Hyaena brunnea* Thunberg, 1820 |
| *Proteles cristata* (Sparrman, 1783) |
| **Mustelidae** |
| *Aonyx capensis* (Schinz, 1821) |
| ***Aonyx congicus*** Lönnberg, 1910 |
| *Hydrictis maculicollis* Lichtenstein, 1835 |
| *Ictonyx striatus* (Perry, 1810) |
| *Mellivora capensis* (Schreber, 1776) |
| *Poecilogale albinucha* (Gray, 1864) |
| **Nandiniidae** |
| *Nandinia binotata* (Gray, 1830) |
| **Otariidae** |
| *Arctocephalus pusillus* (Schreber, 1775) |
| **Viverridae** |
| *Civettictis civetta* (Schreber, 1776) |
| *Genetta angolensis* Bocage, 1882 |
| *Genetta genetta* (Linnaeus, 1758) |
| *Genetta maculata* (Gray, 1830) |
| **Chiroptera** |
| **Emballonuridae** |
| *Coleura afra* (Peters, 1852) |
| ***Saccolaimus peli* (Temminck 1853)** |
| *Taphozous mauritianus* E. Geoffroy, 1818 |
| **Hipposideridae** |
| *Hipposideros caffer* (Sundevall, 1846) |
| *Hipposideros gigas* (Wagner, 1845) |
| *Hipposideros ruber* (Noack, 1893) |
| *Hipposideros vittatus* (Peters, 1852)  **Rhinonycteridae** |
| ***Triaenops afer* Peters 1877** |
| **Miniopteridae** |
| *Miniopterus natalensis* (A. Smith, 1833) |
| **Molossidae** |
| **?*Mops midas* (Sundevall, 1843)** |
| *Chaerephon ansorgei* (Thomas, 1913) |
| *Chaerephon chapini* J. A. Allen, 1917 |
| *Chaerephon nigeriae* Thomas, 1913 |
| *Chaerephon pumilus* (Cretzschmar, 1826) |
| *Mops condylurus* (A. Smith, 1833) |
| *Mops niveiventer* Cabrera & Ruxton, 1926 |
| *Otomops martiensseni* (Matschie, 1897) |
| *Tadarida aegyptiaca* (E. Geoffroy, 1818) |
| **Nycteridae** |
| *Nycteris arge* Thomas, 1903 |
| *Nycteris hispida* (Schreber, 1775) |
| ***Nycteris intermedia* Aellen, 1959** |
| *Nycteris macrotis* Dobson, 1876 |
| *Nycteris nana* (K. Andersen, 1912) |
| *Nycteris thebaica* E. Geoffroy, 1818 |
| **Pteropodidae** |
| *Eidolon helvum* (Kerr, 1792) |
| *Epomophorus angolensis* Gray, 1870 |
| *Epomophorus crypturus* Peters, 1852 |
| *Epomophorus grandis* (Sanborn, 1950) |
| *Epomophorus wahlbergi* (Sundevall, 1846) |
| *Epomops dobsonii* (Bocage, 1889) |
| *Epomops franqueti* (Tomes, 1860) |
| *Hypsignathus monstrosus* H. Allen, 1861 |
| *Megaloglossus woermanni* Pagenstecher, 1885 |
| *Micropteropus intermedius* Hayman, 1963 |
| *Micropteropus pusillus* (Peters, 1868) |
| *Myoncyteris angolensis* (Bocage, 1898) |
| *Myonycteris torquata* (Dobson, 1878) |
| ***Plerotes anchietae* (Seabra, 1900)** |
| *Rousettus aegyptiacus* (E. Geoffroy, 1810) |
| **Rhinolophidae** |
| ?*Rhinolophus eloquens* K. Andersen, 1905 |
| *Rhinolophus damarensis* Roberts, 1946 |
| ***Rhinolophus denti* Thomas, 1904** |
| *Rhinolophus fumigatus* Rüppell, 1842 |
| *Rhinolophus lobatus* Peters, 1852 |
| **Vespertilionidae** |
| **?*Kerivoula argentata* Tomes, 1861** |
| **?*Pipistrellus hesperidus* Temminck 1840** |
| *Cistugo seabrae* Thomas, 1912 |
| *Eptesicus hottentotus* (A. Smith, 1833) |
| *Glauconycteris argentata* (Dobson, 1875) |
| *Glauconycteris beatrix* Thomas, 1901 |
| *Glauconycteris variegata* (Tomes, 1861) |
| *Hypsugo anchietae* (Seabra, 1900) |
| *Hypsugo crassulus* (Thomas, 1904) |
| ***Kerivoula lanosa* (A. Smith, 1847)** |
| *Laephotis angolensis* Monard, 1935 |
| *Laephotis botswanae* Setzer, 1971 |
| *Mimetillus thomasi* Hinton, 1920 |
| *Myotis bocagii* Peters, 1870 |
| *Myotis welwitschii* (Gray, 1866) |
| *Neoromicia capensis* (A. Smith, 1829) |
| *Neoromicia grandidieri* (Dobson, 1876) |
| *Neoromicia nana (Peters, 1852)* |
| *Neoromicia tenuipinnis* (Peters 1872) |
| *Neoromicia zuluensis* (Roberts, 1924) |
| ***Nycticeinops schlieffeni* (Peters, 1859)** |
| *Pipistrellus rueppellii* (J. Fischer, 1829) |
| ***Pipistrellus rusticus* (Tomes, 1861)** |
| *Scotoecus hindei* Thomas, 1901 |
| *Scotophilus dinganii* (A. Smith, 1833) |
| *Scotophilus leucogaster* (Cretzschmar, 1826) |
| ***Scotophilus viridis* (Peters, 1852)** |
| **Erinaceomorpha** |
| **Erinaceidae** |
| *Atelerix frontalis* (A. Smith, 1831) |
| **Hyracoidea** |
| **Procaviidae** |
| *Dendrohyrax arboreus* (A. Smith, 1827) |
| *Dendrohyrax dorsalis* (Fraser, 1854) |
| *Heterohyrax brucei* (Gray, 1868) |
| *Procavia capensis* (Pallas, 1766) |
| **Lagomorpha** |
| **Leporidae** |
| *Lepus victoriae* Thomas, 1893 |
| **Macroscelidea** |
| **Macroscelididae** |
| *Elephantulus brachyrhynchus* (A. Smith, 1836) |
| *Elephantulus intufi* (A. Smith, 1836) |
| **Perissodactyla** |
| **Equidae** |
| *Equus quagga* Boddaert, 1785 |
| *Equus zebra* Linnaeus, 1758 |
| **Rhinocerotidae** |
| *Diceros bicornis* (Linnaeus, 1758) |
| **Pholidota** |
| **Manidae** |
| *Manis temminckii* Smuts, 1832 |
| ***Phataginus tricuspis*** (Rafinesque, 1821) |
| **Primates** |
| **Cercopithecidae** |
| *Cercocebus torquatus* (Kerr in Linnaeus, 1792) |
| *Cercopithecus ascanius* (Audebert, 1799) |
| *Cercopithecus cephus* (Linnaeus, 1758) |
| *Cercopithecus mitis* Wolf, 1822 |
| *Cercopithecus neglectus* Schlegel, 1876 |
| *Cercopithecus nictitans* (Linnaeus, 1766) |
| *Chlorocebus cynosuros* (Scopoli, 1786) |
| *Colobus angolensis* Sclater, 1860 |
| *Lophocebus albigena* (Gray, 1850) |
| *Miopithecus talapoin* (Schreber, 1774) |
| *Papio kindae* Lönnberg, 1919 |
| *Papio ursinus* (Kerr, 1792) |
| **Galagidae** |
| *Galago moholi* A. Smith, 1836 |
| *Galagoides demidoff* (G. Fischer, 1806) |
| *Otolemur crassicaudatus* (Geoffroy, 1812) |
| **Hominidae** |
| *Gorilla gorilla* (Savage in Savage and Wyman, 1847) |
| ***Pan troglodytes*** (Blumenbach, 1775) |
| **Lorisidae** |
| *Perodicticus potto* (Müller, 1766) |
| **Proboscidea** |
| **Elephantidae** |
| *Loxodonta africana* (Blumenbach, 1797) |
| ***Loxodonta cyclotis*** (Matschie, 1900) |
| **Rodentia** |
| **Anomaluridae** |
| ***Anomalurus beecrofti*** Fraser, 1853 |
| *Anomalurus derbianus* (Gray, 1842) |
| **Bathyergidae** |
| *Fukomys bocagei* (de Winton, 1897) |
| *Fukomys mechowi* (Peters, 1881) |
| **Gliridae** |
| *Graphiurus angolensis* de Winton, 1897 |
| *Graphiurus kelleni* (Reuvens, 1890) |
| *Graphiurus lorraineus* Dollman, 1910 |
| *Graphiurus monardi* (St. Leger, 1936) |
| *Graphiurus rupicola* (Thomas & Hinton, 1925) |
| **Hystricidae** |
| *Hystrix africaeaustralis* Peters, 1852 |
| **Muridae** |
| *Aethomys bocagei* (Thomas, 1904) |
| *Aethomys chrysophilus* (de Winton, 1897) |
| *Aethomys kaiseri* (Noack, 1887) |
| *Aethomys nyikae* (Thomas, 1897) |
| *Aethomys thomasi* (de Winton, 1897) |
| *Colomys goslingi* Thomas & Wroughton, 1907 |
| *Dasymys cabrali* (Verheyeni et al. 2003) |
| *Dasymys incomtus* (Sundevall, 1847) |
| *Dasymys nudipes* (Peters, 1870) |
| *Desmodillus auricularis* (A. Smith, 1834) |
| *Gerbilliscus brantsii* (A. Smith, 1836) |
| *Gerbilliscus leucogaster* (Peters, 1852) |
| *Gerbilliscus paeba* (A. Smith, 1836) |
| *Gerbilliscus setzeri* (Schlitter, 1973) |
| *Gerbilliscus validus* (Bocage, 1890) |
| *Grammomys dolichurus* (Smuts, 1832) |
| *Grammomys poensis* (Eisentraut, 1965) |
| *Hylomyscus carillus* (Thomas, 1904) |
| *Lemniscomys griselda* (Thomas, 1904) |
| *Lemniscomys striatus* (Linnaeus, 1758) |
| *Lophuromys angolensis* Verheyen et al., 2000 |
| *Lophuromys rita* Dollman, 1910 |
| *Malacomys longipes* Milne-Edwards, 1877 |
| *Mastomys natalensis* (A. Smith, 1834) |
| *Mastomys shortridgei* (St Leger, 1933) |
| *Micaelamys namaquensis* (A. Smith, 1834) |
| *Mus callewaerti* (Thomas, 1925) |
| **?** ***Mus indutus* (Thomas, 1910)** |
| *Mus minutoides* A. Smith, 1834 |
| *Mus sorella* (Thomas, 1909) |
| ***Mus setzeri*** Petter, 1978 |
| *Mus triton* (Thomas, 1909) |
| *Myomyscus angolensis* (Bocage, 1890) |
| *Oenomys hypoxanthus* (Pucheran, 1855) |
| *Otomys anchietae* (Bocage, 1882) |
| *Otomys angoniensis* Wroughton, 1906 |
| *Otomys cuanzensis* Hill & Carter, 1937 |
| *Pelomys campanae* (Huet, 1888) |
| *Pelomys fallax* (Peters, 1852) |
| *Pelomys minor* Cabrera & Ruxton, 1926 |
| *Praomys coetzeei* Van der Straeten, 2008 |
| *Praomys jacksoni* (de Winton, 1897) |
| ***Rhabdomys bechuanae*** (Thomas, 1893) |
| *Rhabdomys dilectus* (de Winton, 1897) |
| *Thallomys nigricauda* (Thomas, 1882) |
| *Zelotomys hildegardeae* (Thomas, 1902) |
| ***Zelotomys woosnami*** *(Schwann, 1906)* |
| **Nesomyidae** |
| ***Cricetomys emini*** Wroughton, 1910 |
| *Cricetomys ansorgei* Thomas, 1904 |
| ***Dendromus leucostomus*** Monard, 1933 |
| *Dendromus melanotis* (A. Smith, 1834) |
| *Dendromus mystacalis* (Heuglin, 1863) |
| *Dendromus nyikae* Wroughton, 1909 |
| *Dendromus vernayi* Hill & Carter, 1937 |
| ***Malacothrix typica*** (A. Smith, 1834) |
| *Petromyscus collinus* (Thomas & Hinton, 1925) |
| *Petromyscus shortridgei* Thomas, 1926 |
| *Saccostomus campestris* Peters, 1846 |
| *Steatomys bocagei* Thomas, 1892 |
| *Steatomys krebsii* Peters, 1852 |
| *Steatomys parvus* Rhoads, 1896 |
| *Steatomys pratensis* Peters, 1846 |
| **Pedetidae** |
| *Pedetes capensis* (Forster, 1778) |
| **Petromuridae** |
| ***Petromus typicus A. Smith, 1831*** |
| **Sciuridae** |
| *Funisciurus bayonii* (Bocage, 1890) |
| *Funisciurus congicus* (Kuhl, 1820) |
| ***Funisciurus lemniscatus*** (Le Conte, 1857) |
| *Funisciurus pyrropus* (F. Cuvier, 1833) |
| *Heliosciurus gambianus* (Ogilby, 1835) |
| *Paraxerus boehmi* (Reichenow, 1886) |
| *Paraxerus cepapi* (A. Smith, 1836) |
| *Protoxerus stangeri* (Waterhouse, 1842) |
| *Xerus princeps* (Thomas, 1929) |
| **Thryonomyidae** |
| *Thryonomys swinderianus* (Temminck, 1827) |
| **Soricomorpha** |
| **Soricidae** |
| *Crocidura cyanea* (Duvernoy, 1838) |
| *Crocidura erica* Dollman, 1915 |
| *Crocidura fuscomurina* (Heuglin, 1865) |
| *Crocidura hirta* Peters, 1852 |
| *Crocidura mariquensis* (A. Smith, 1844) |
| *Crocidura nigricans* Bocage, 1889 |
| *Crocidura nigrofusca* Matschie, 1895 |
| *Crocidura olivieri* (Lesson, 1827) |
| *Crocidura parvipes* Osgood, 1910 |
| *Crocidura roosevelti* (Heller, 1910) |
| *Crocidura turba* Dollman, 1910 |
| ***Suncus lixus*** (Thomas, 1898) |
| ***Suncus megalura*** *(Jentink, 1888)* |
| ***Suncus varilla*** (Thomas, 1895) |
| **Tubulidentata** |
| **Orycteropodidae** |
| *Orycteropus afer* (Pallas, 1766) |
