# Supplementary material for: Tapping into technology and the biodiversity informatics revolution: updated terrestrial mammal list of Angola, with new records from the Okavango Basin
Source: Zookeys. 2018 Aug 2;(779):51–88. doi: 10.3897/zookeys.779.25964 (PMC6085403; doi:10.3897/zookeys.779.25964)
Supplement: Supplementary material 2 — Labeled images of example sonograms of each bat species identified [file zookeys-779-051-s002.docx]

APPENDIX 2: Examples of sonograms obtained in AnalookW for each species defined from acoustic surveys of central Angola using files obtained directly from Titley ANABAT SD1 and SD2 detectors or converted from wav or wac files obtained from Wildlife Acoustic Echo Meter 3 or Song Meter 2+ bat detectors.

**Family Hipposideridae**


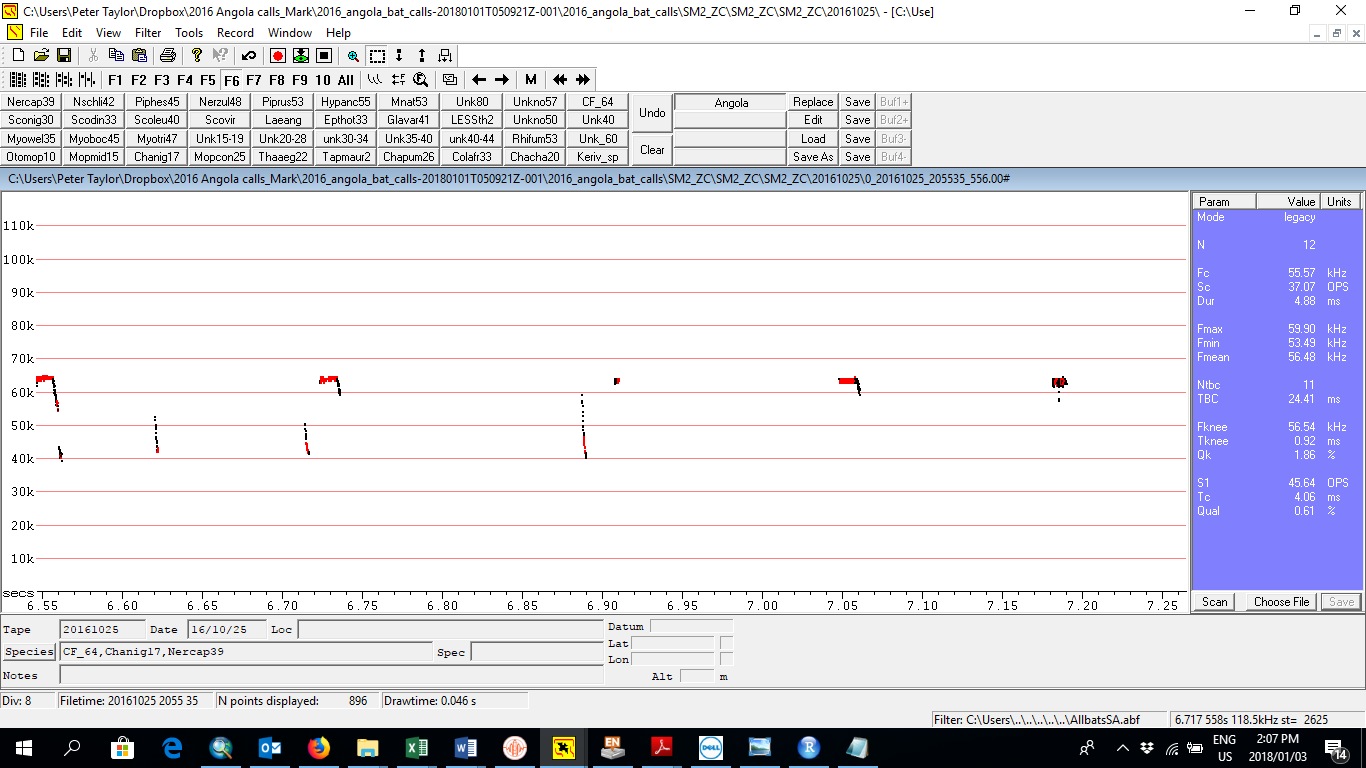


*Hipposideros vittatus*

**Family Rhinolophidae**


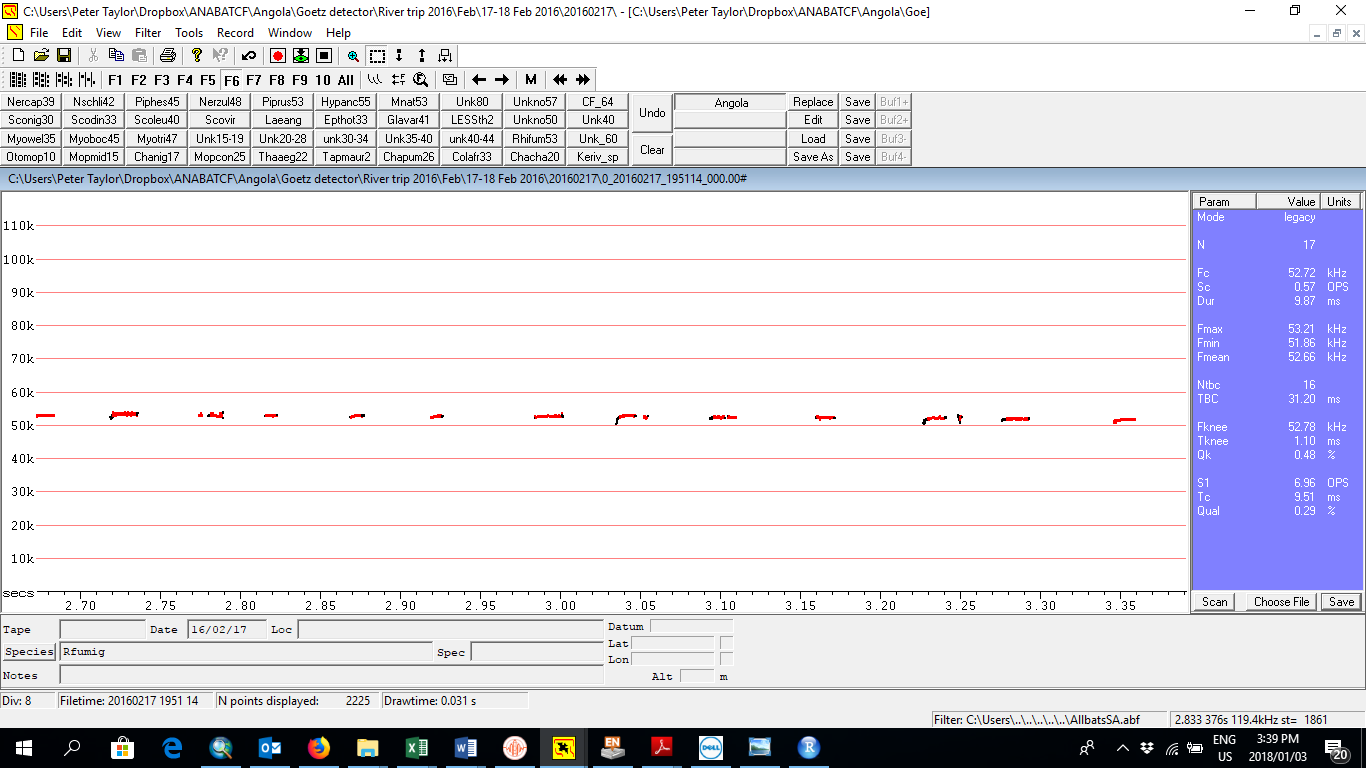


*Rhinolophus fumigatus*

**Family Emballanuridae**


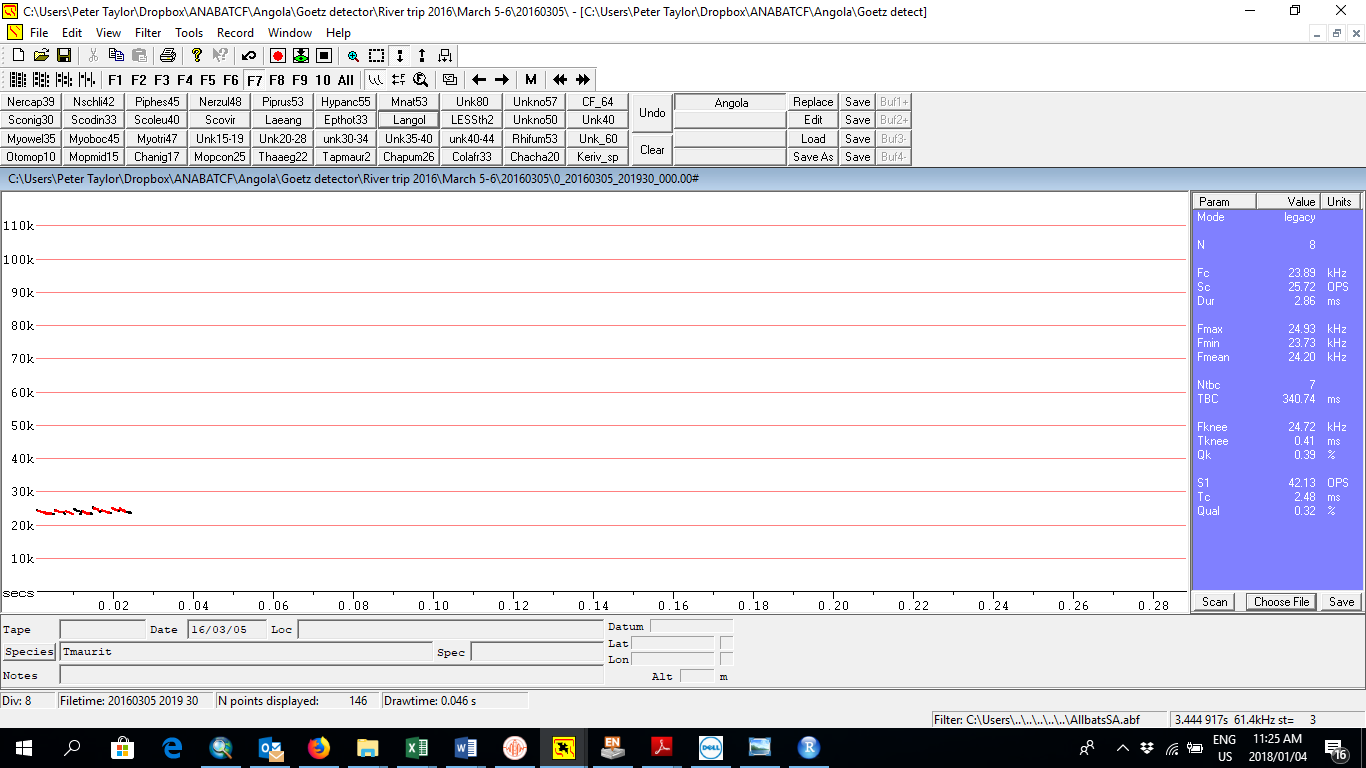


*Taphozous mauritianus*


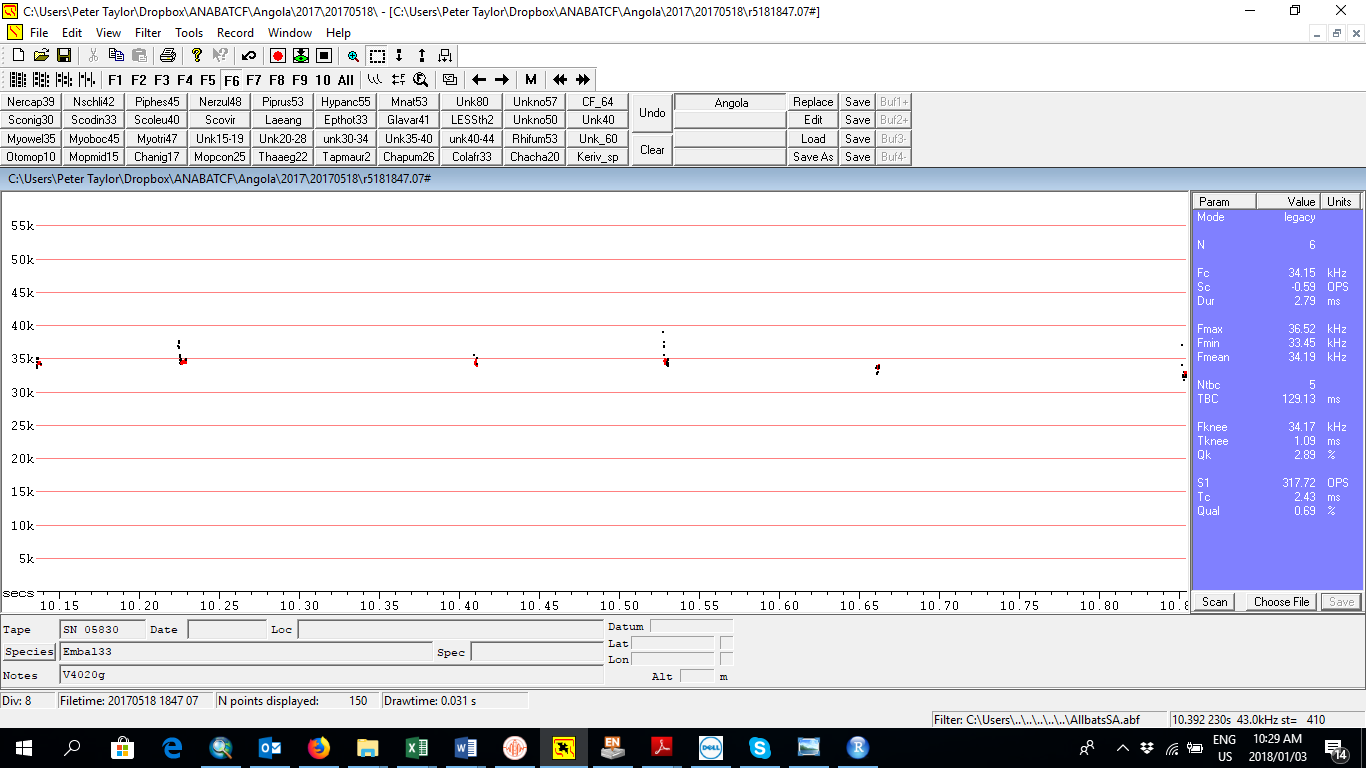


Unknown emballanurid (Fmin 33 kHz) possibly *Coleura afra* or *Taphozous perforatus*

**Family Miniopteridae**


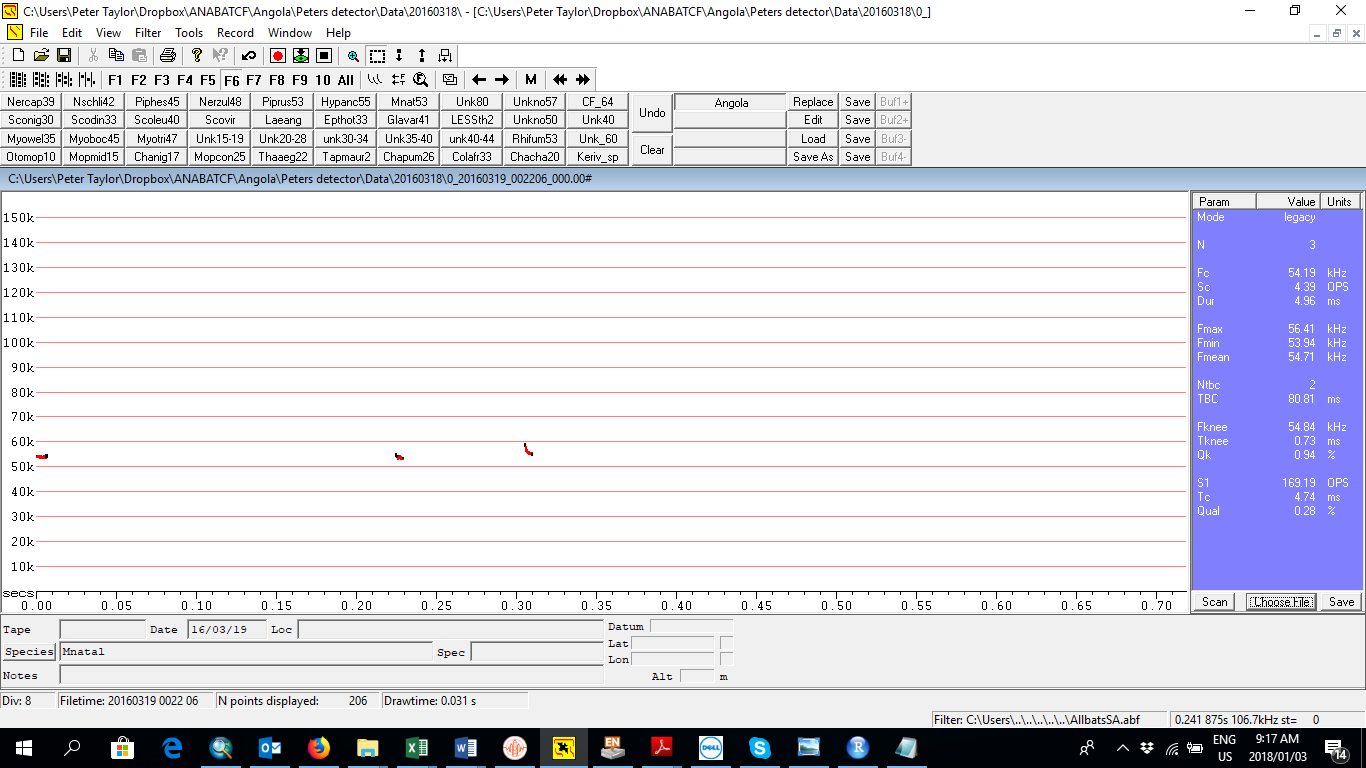


*Miniopterus natalensis*

**Family Vespertilionidae**


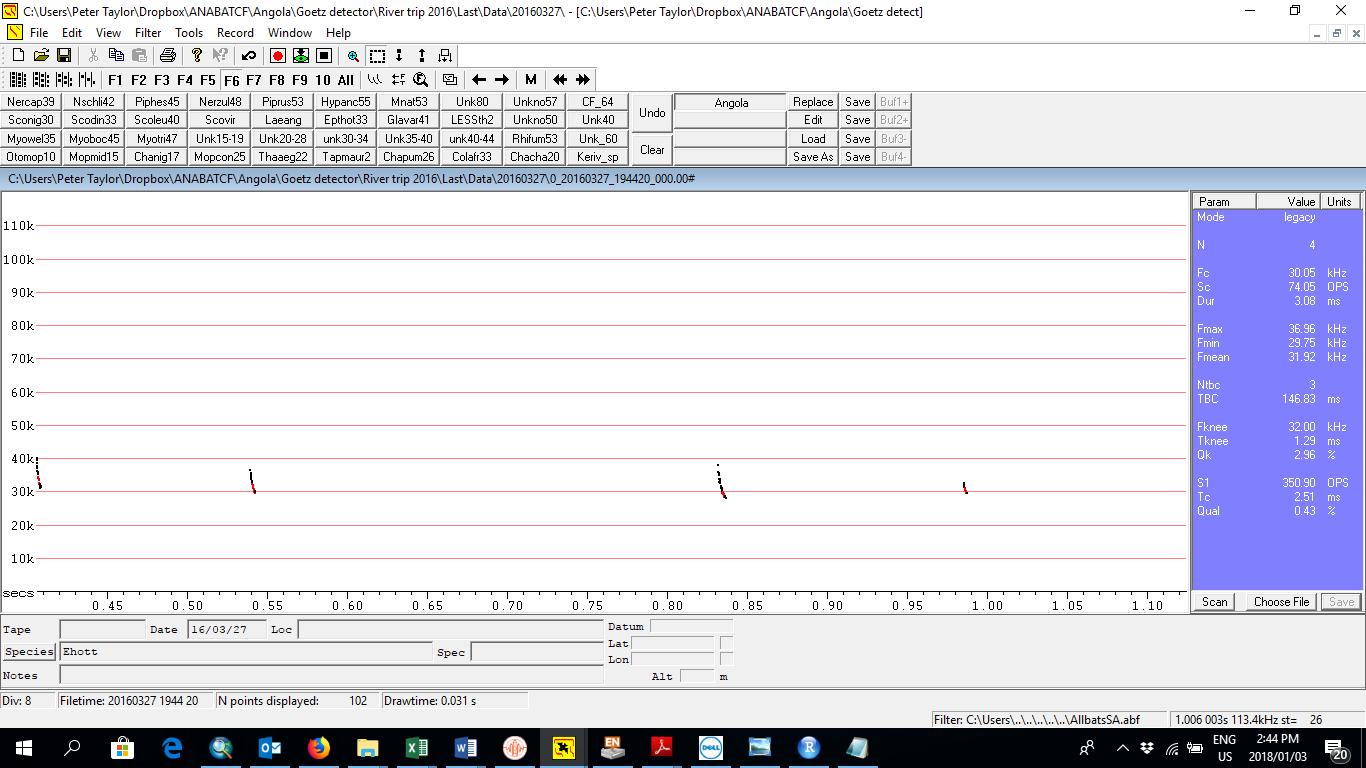


*Eptesicus hottentotus*


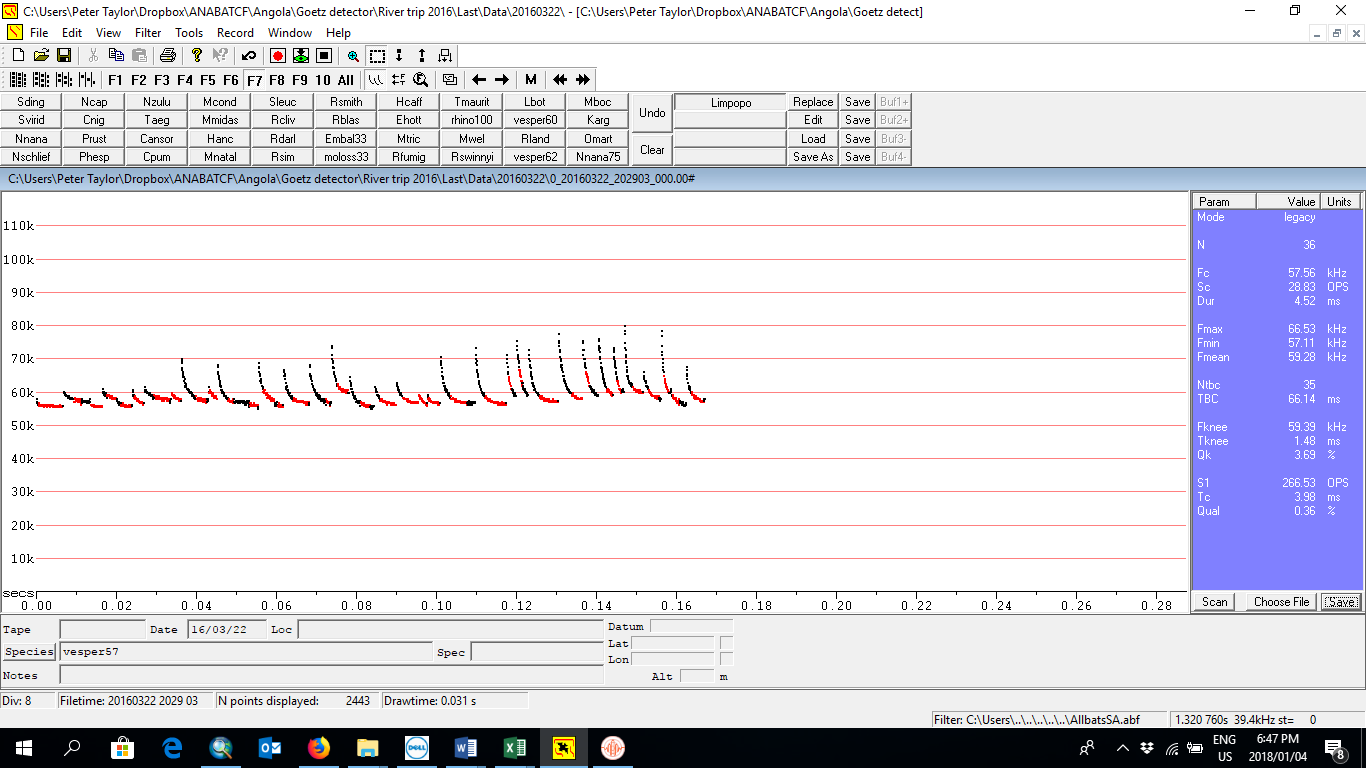


*Hypsugo anchietae*


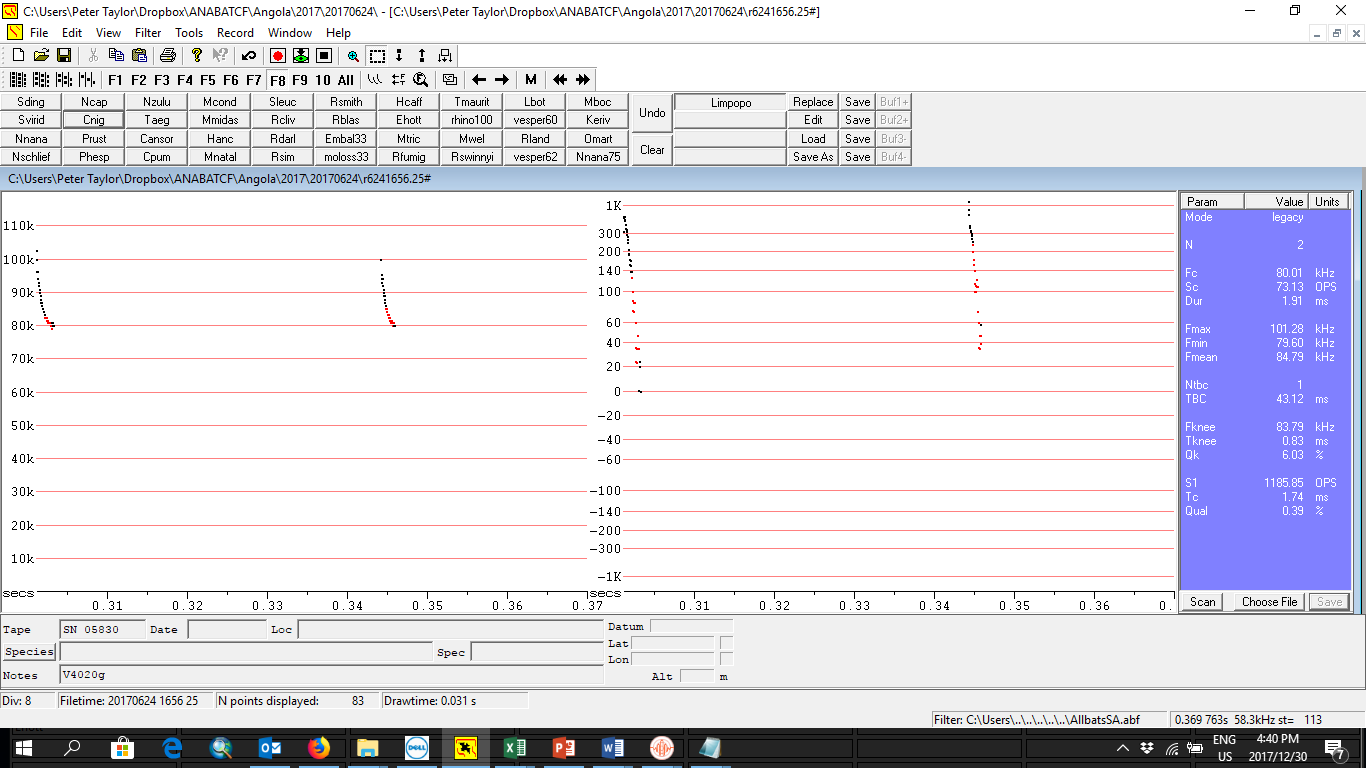


Kerivoula argentata


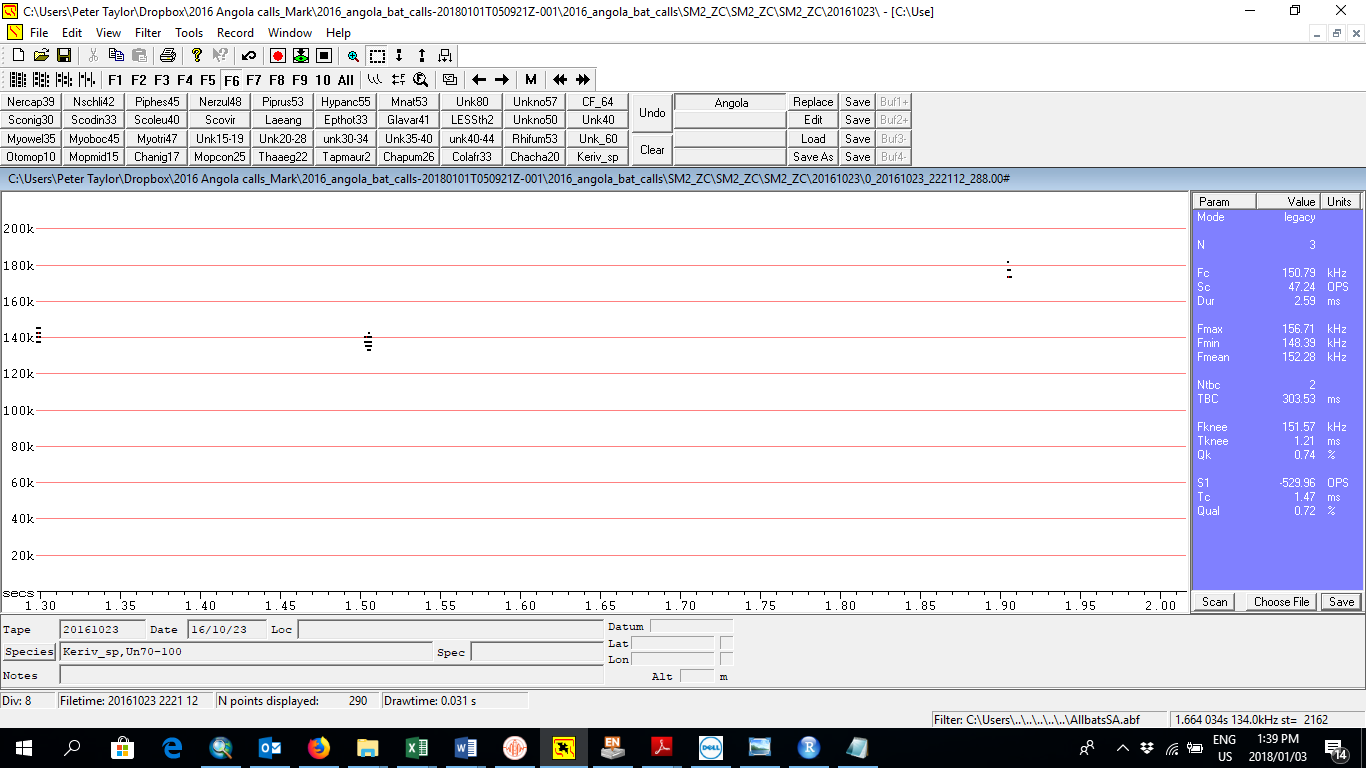


*Kerivoula lanosa*


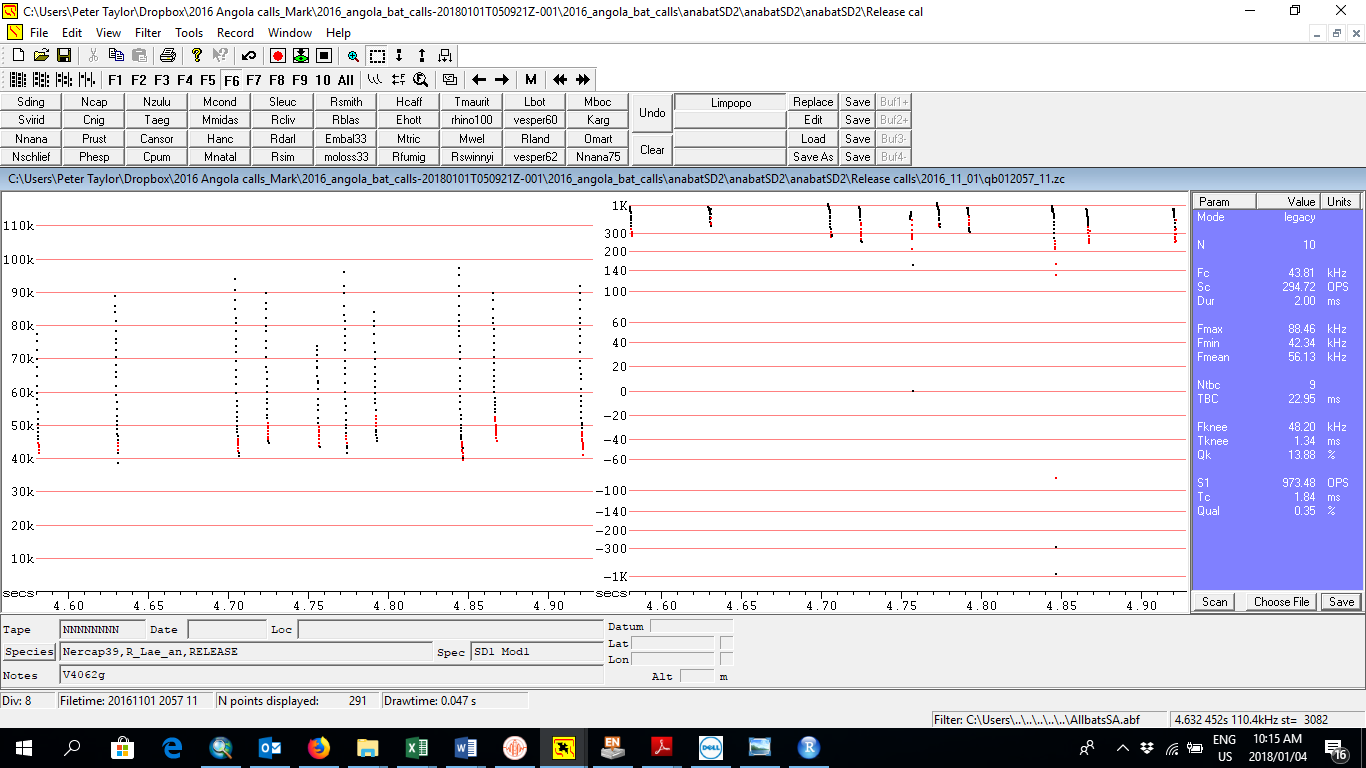


Laephotis angolensis (release call): duration < 2 ms; slope >300


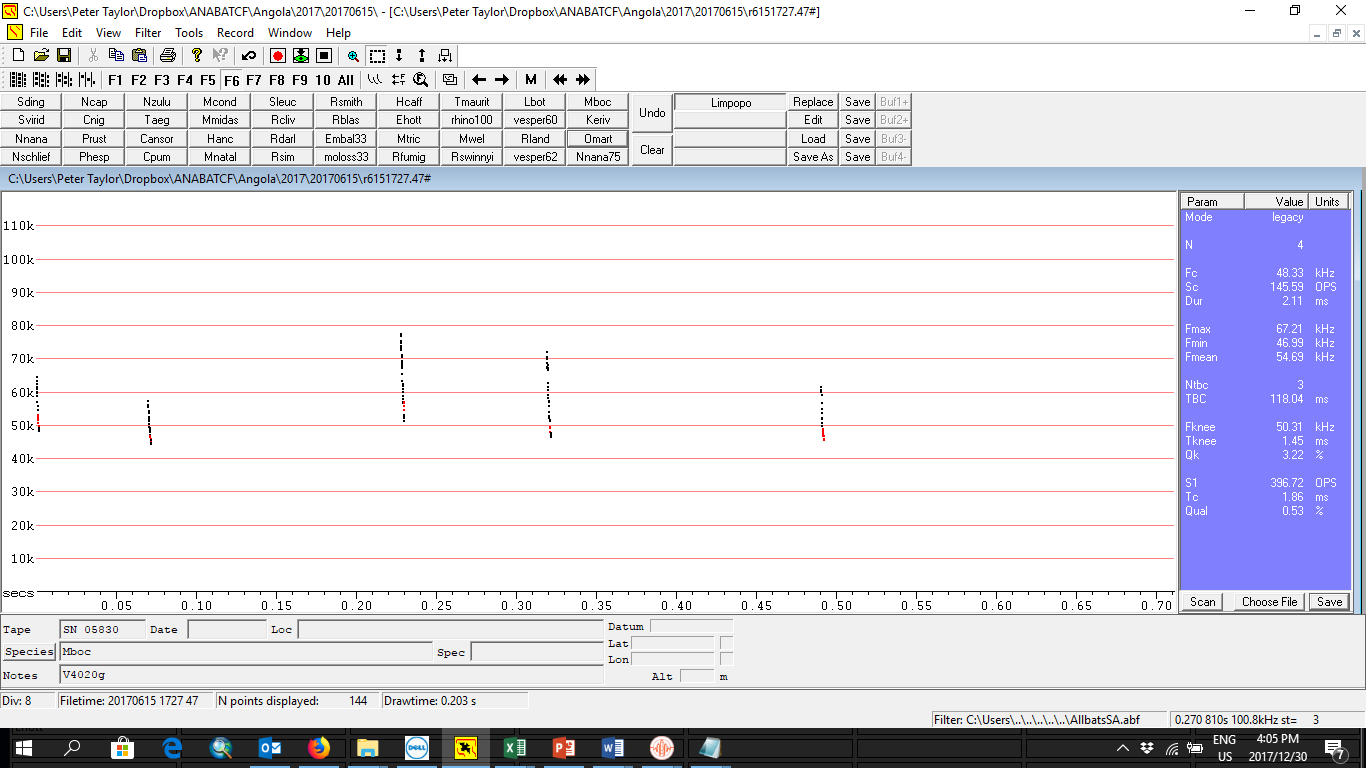


*Laephotis angolensis?*


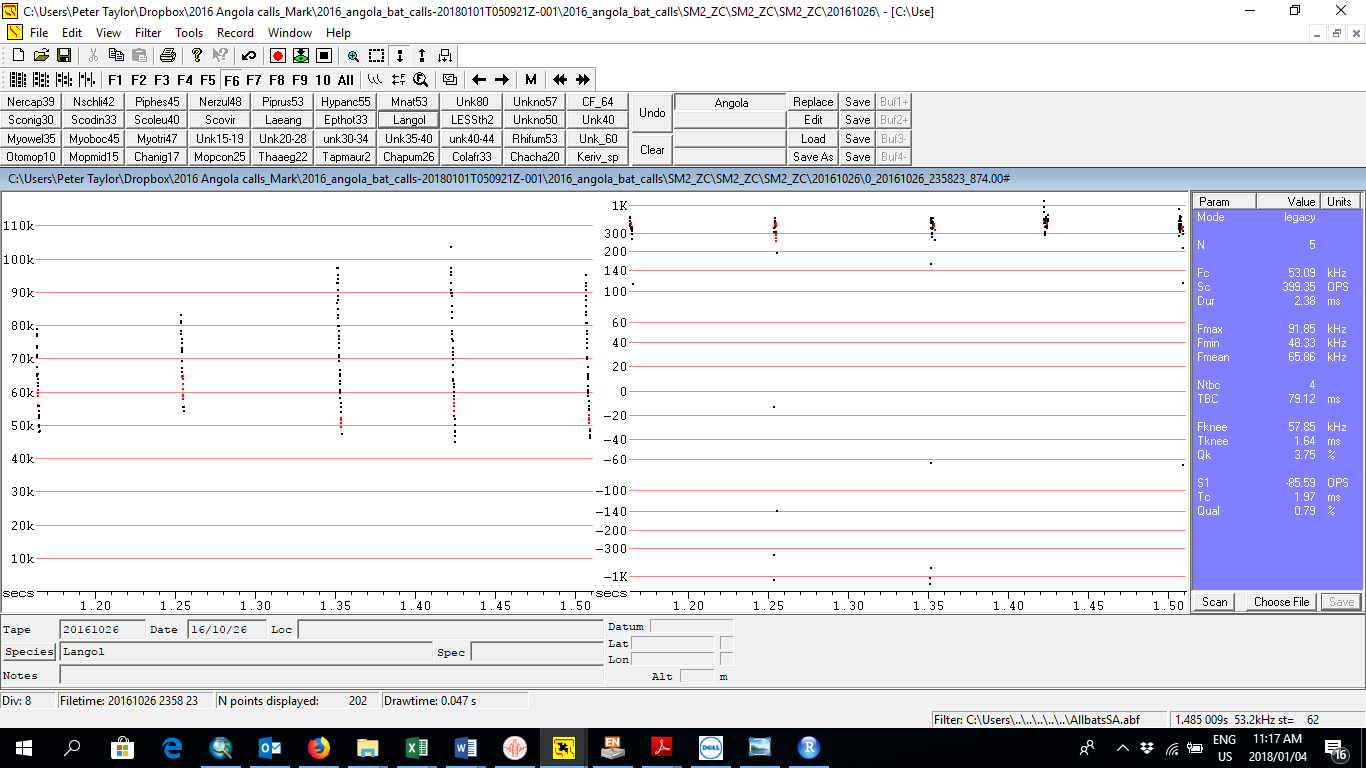


*Laephotis angolensis*?


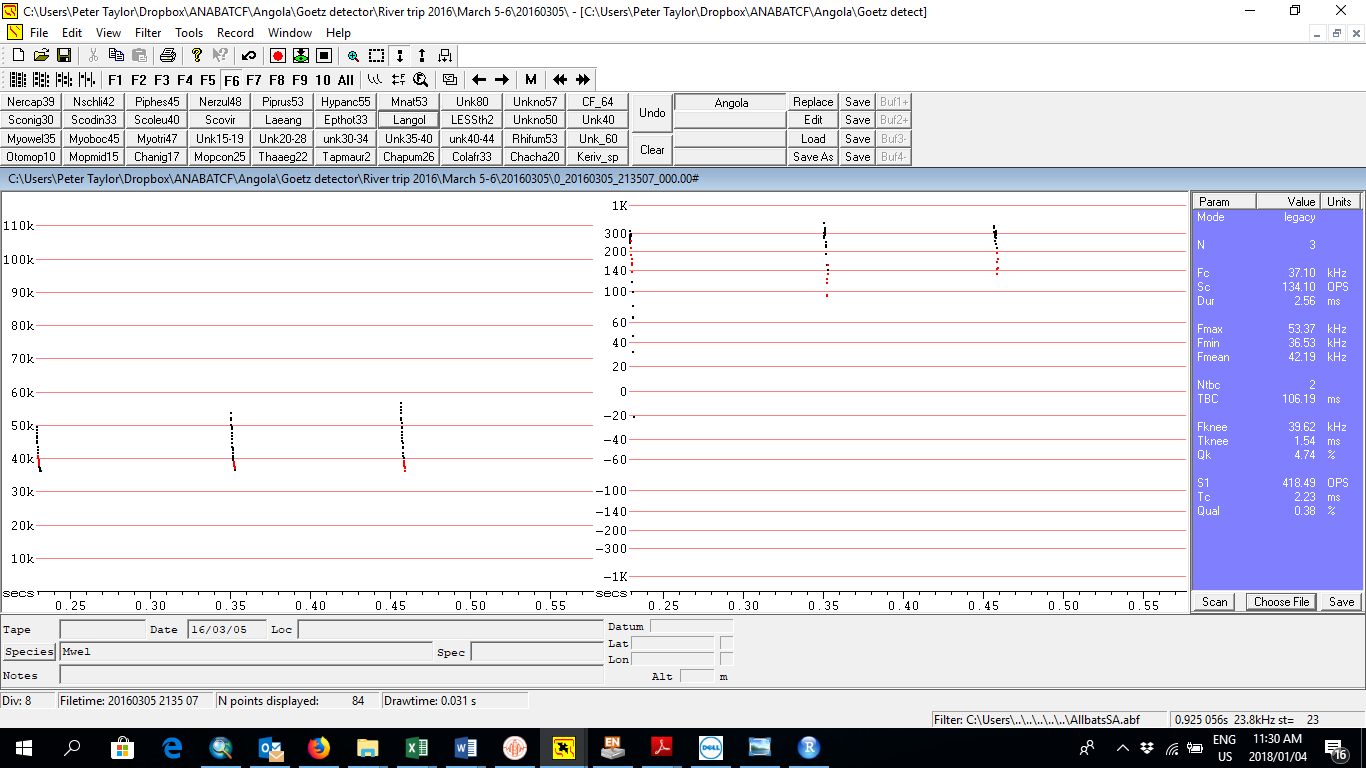


*Myotis bocagii*?


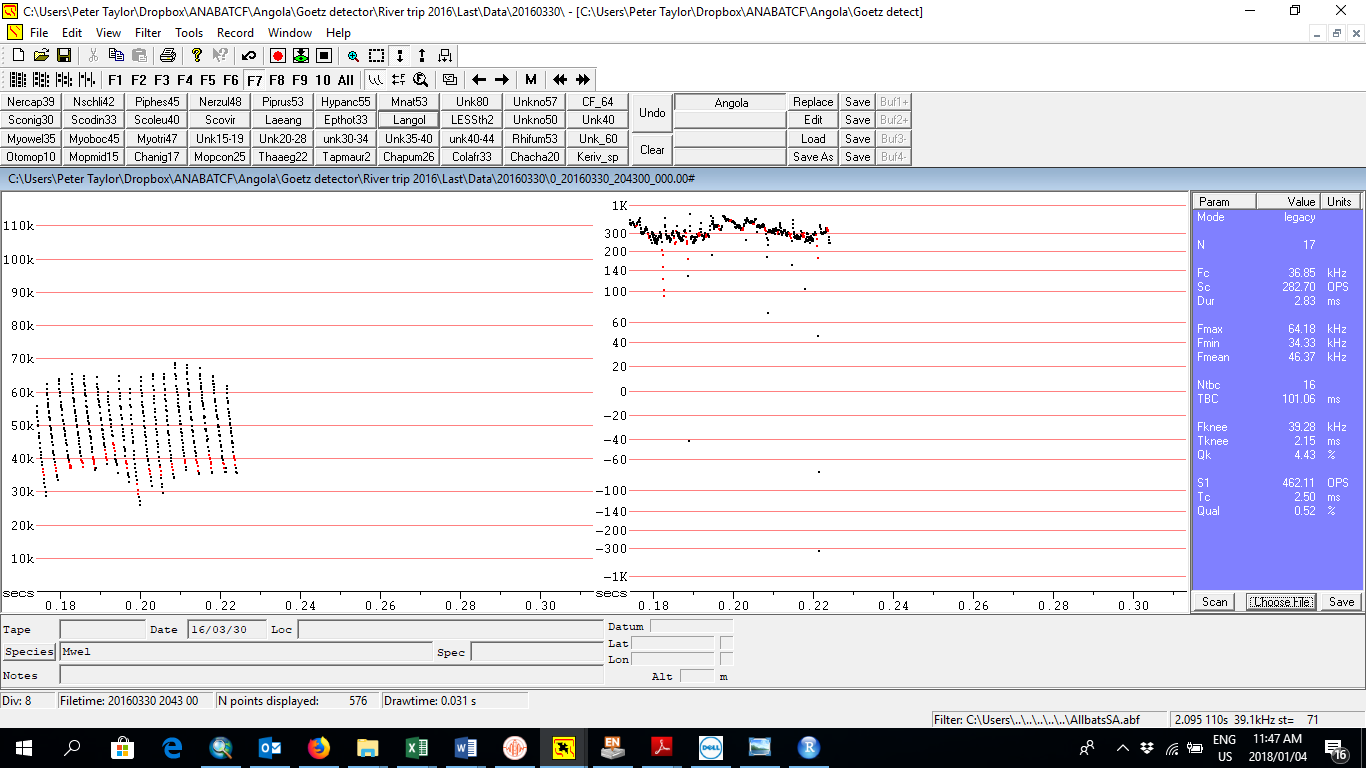


*Myotis welwitschii*


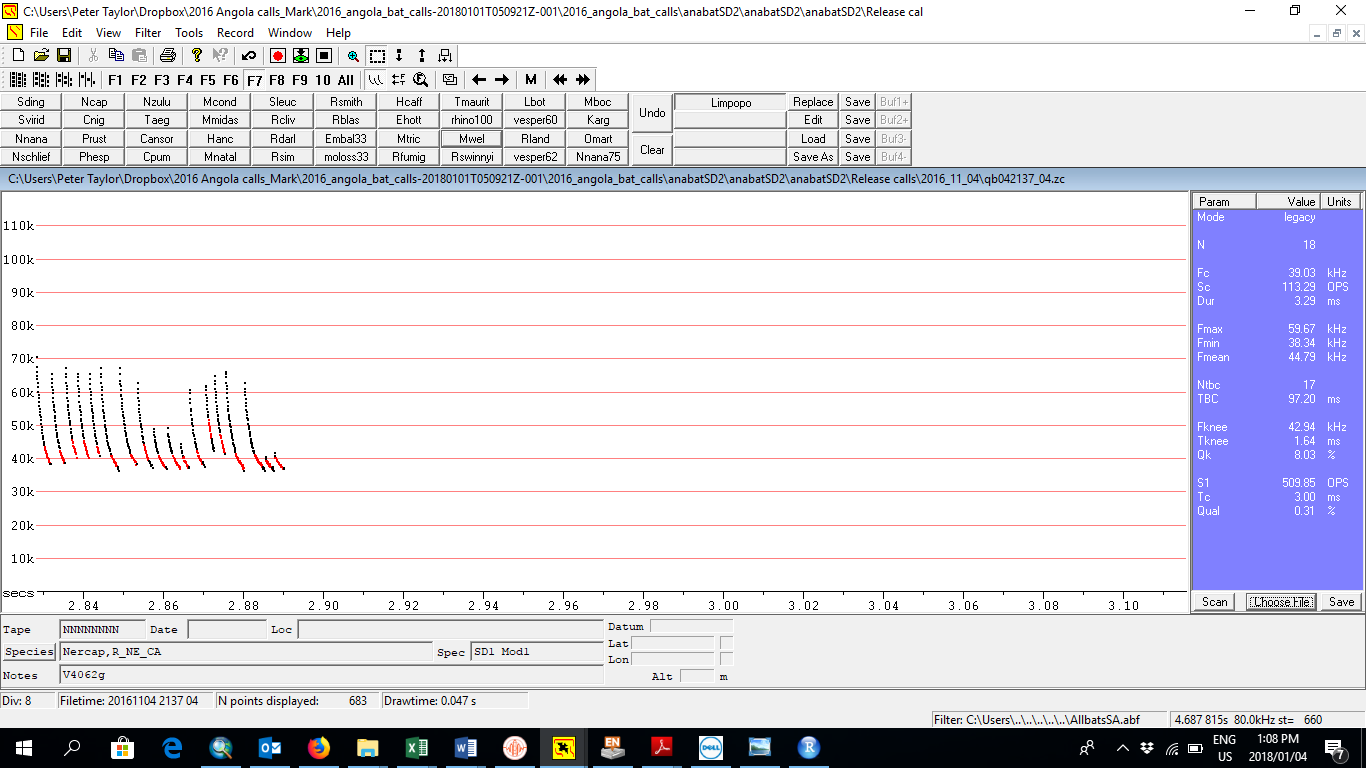


*Neoromicia capensis* release call


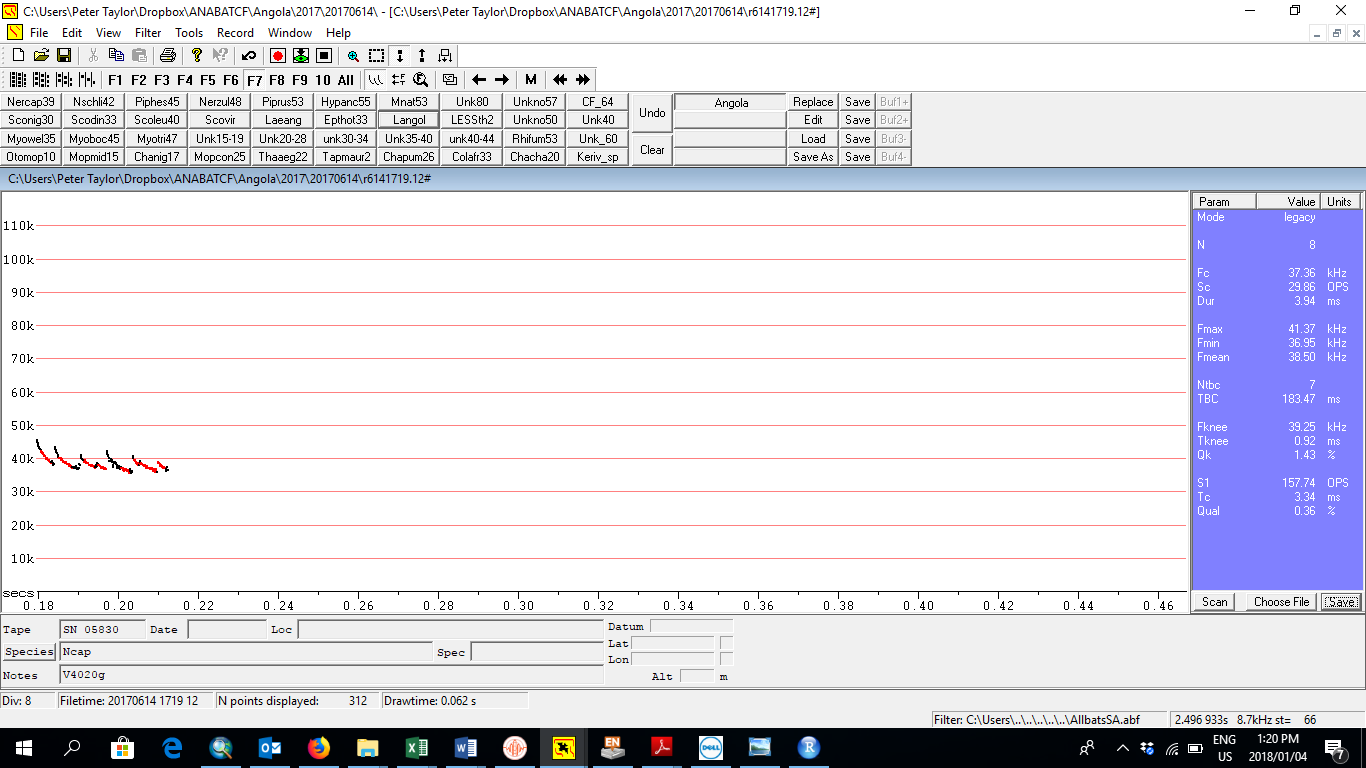


*Neoromicia capensis*


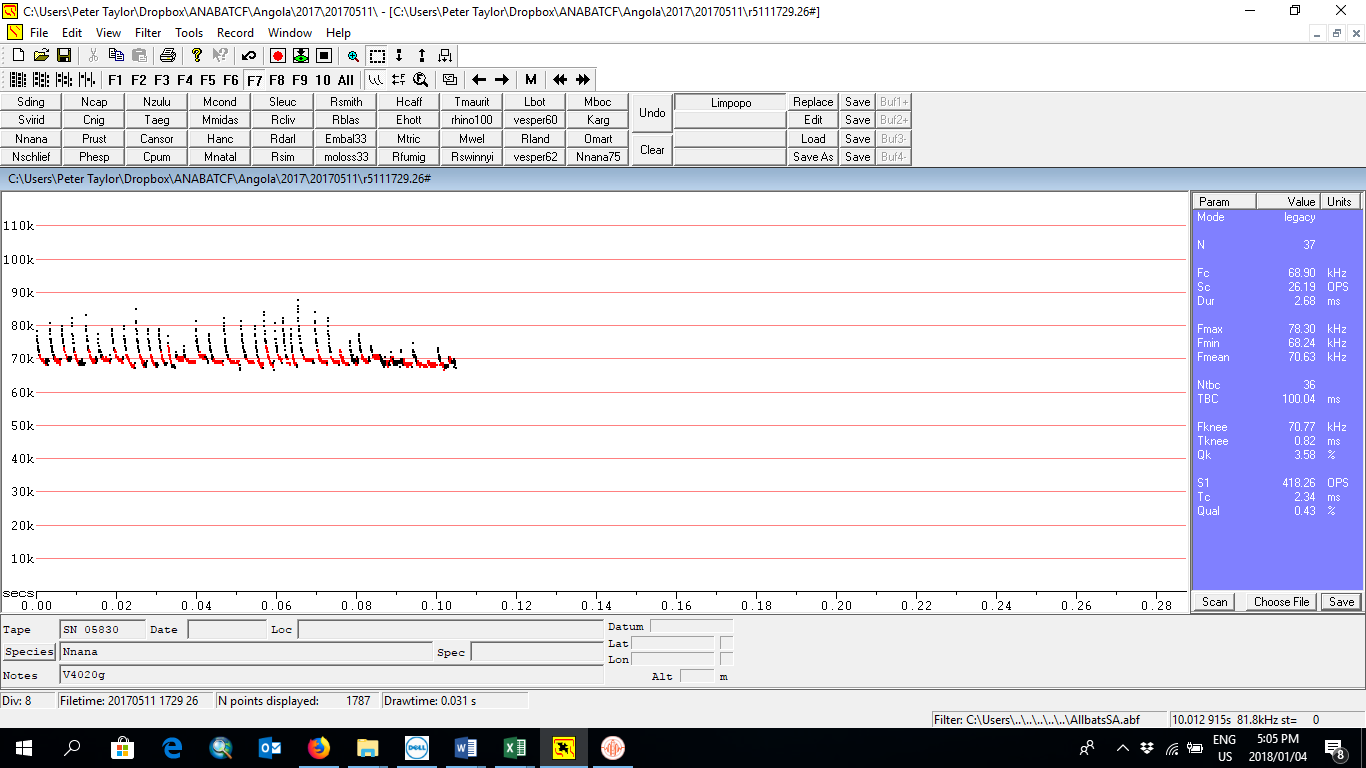


*Neoromicia nana*


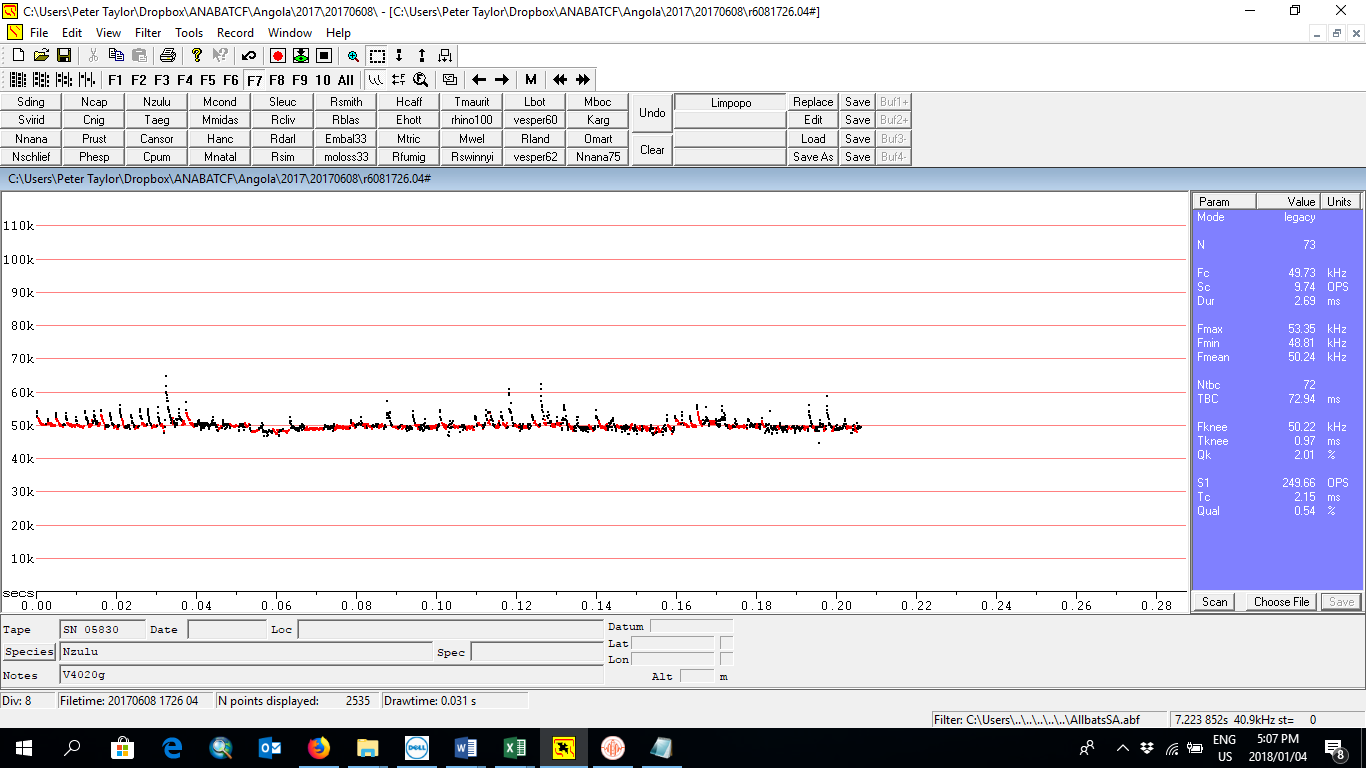


*Neoromicia zuluensis*


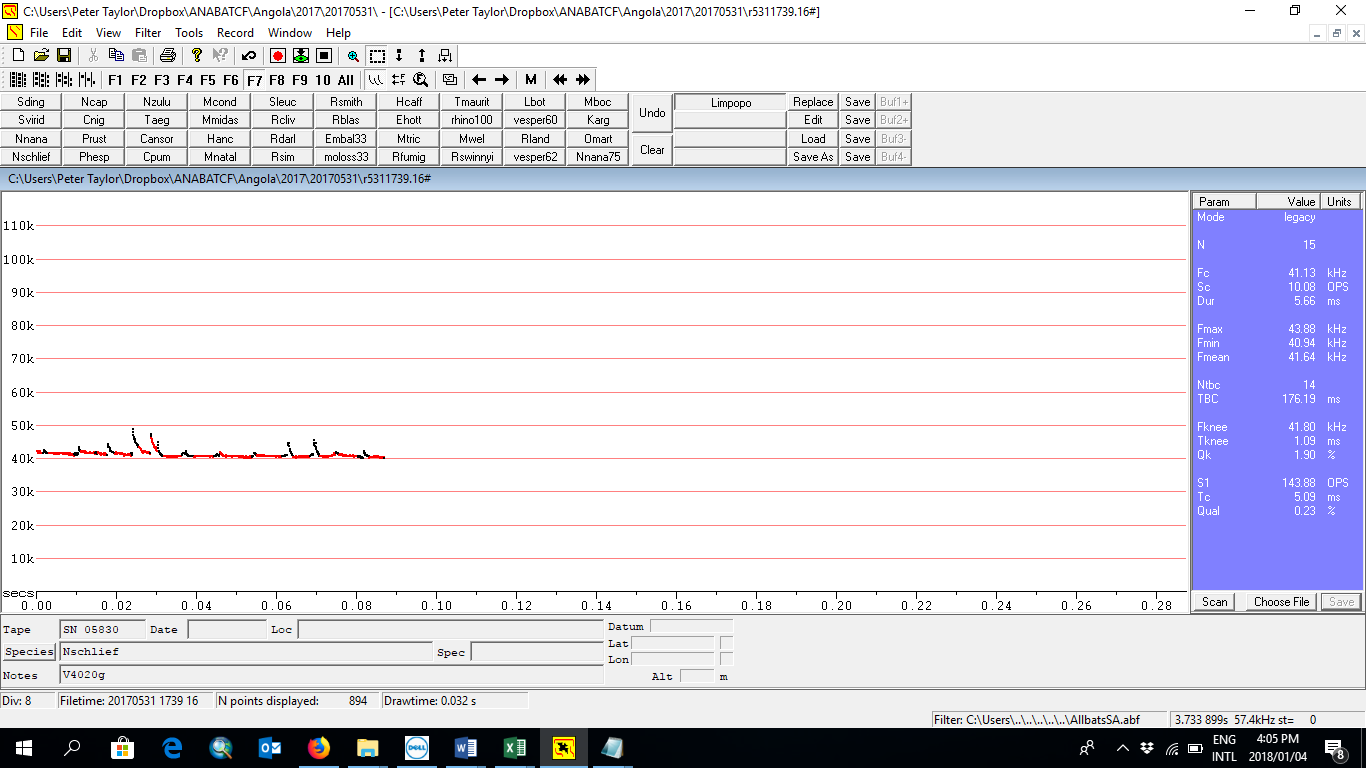


*Nycticeinops schlieffeni*


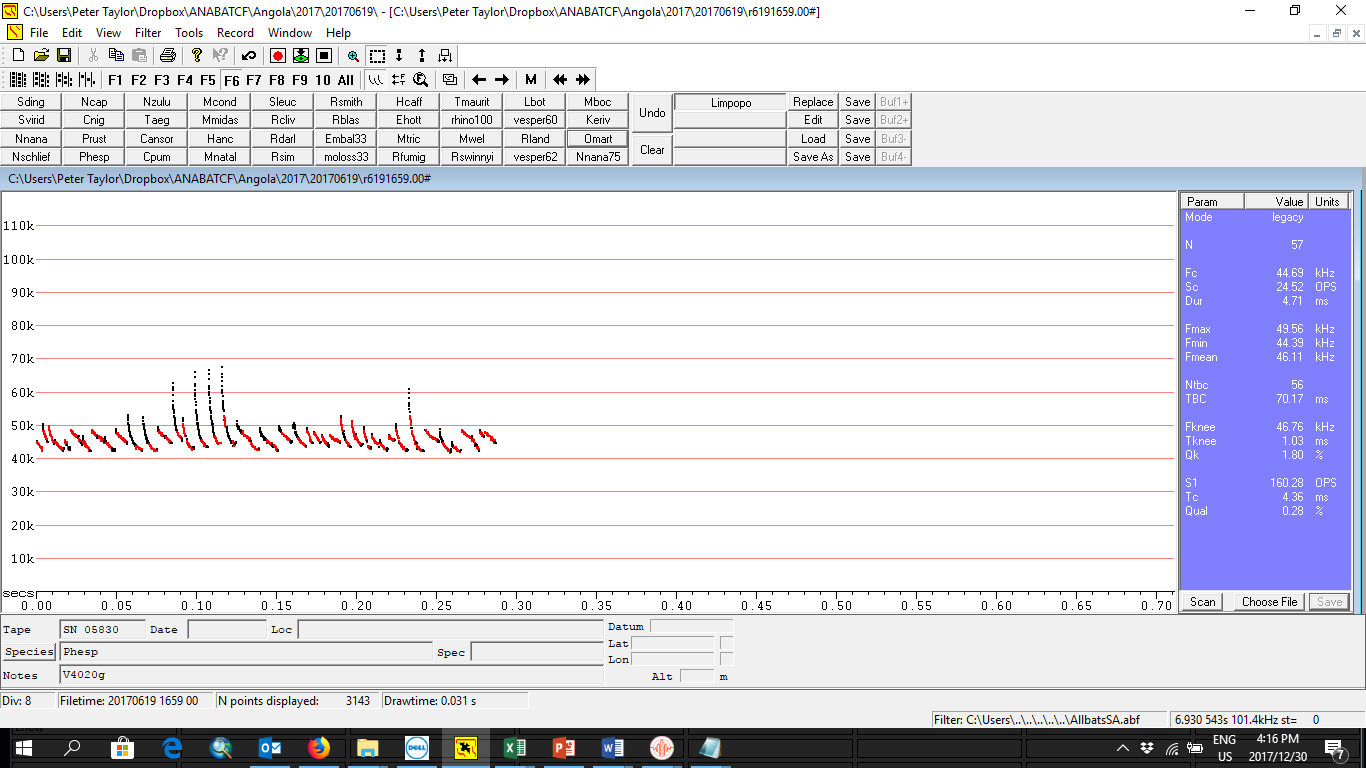


*Pipistrellus hesperidus* (=*L. angolensis*): longer duration


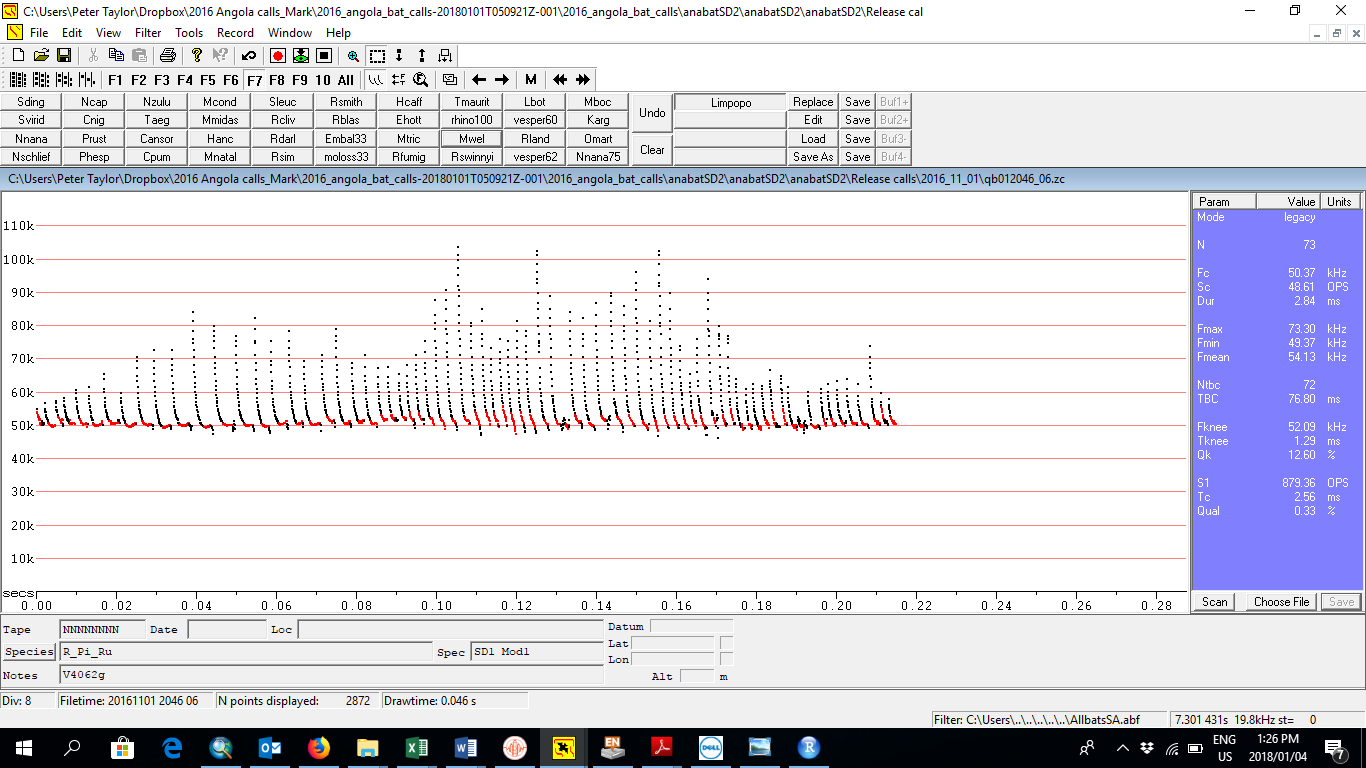


*Pipistrellus rusticus* release call


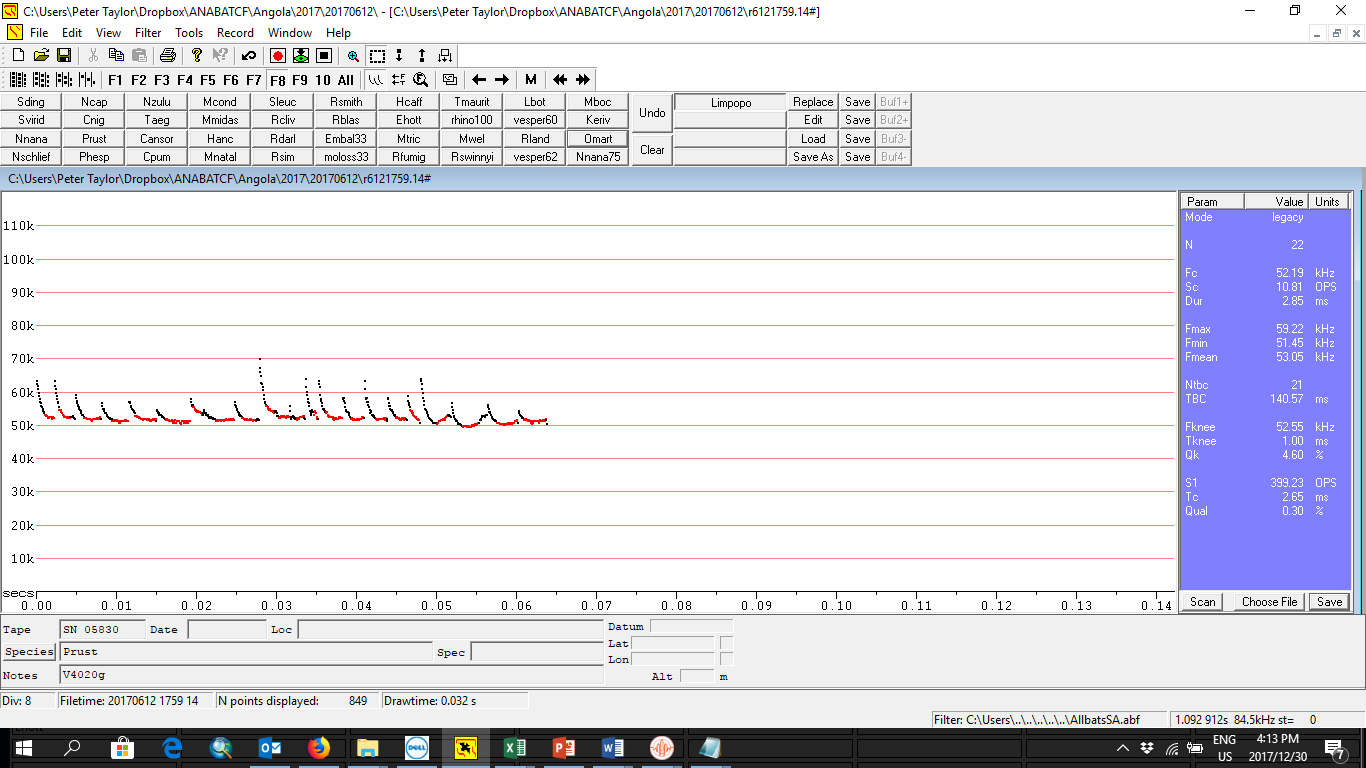


*Pipistrellus rusticus*


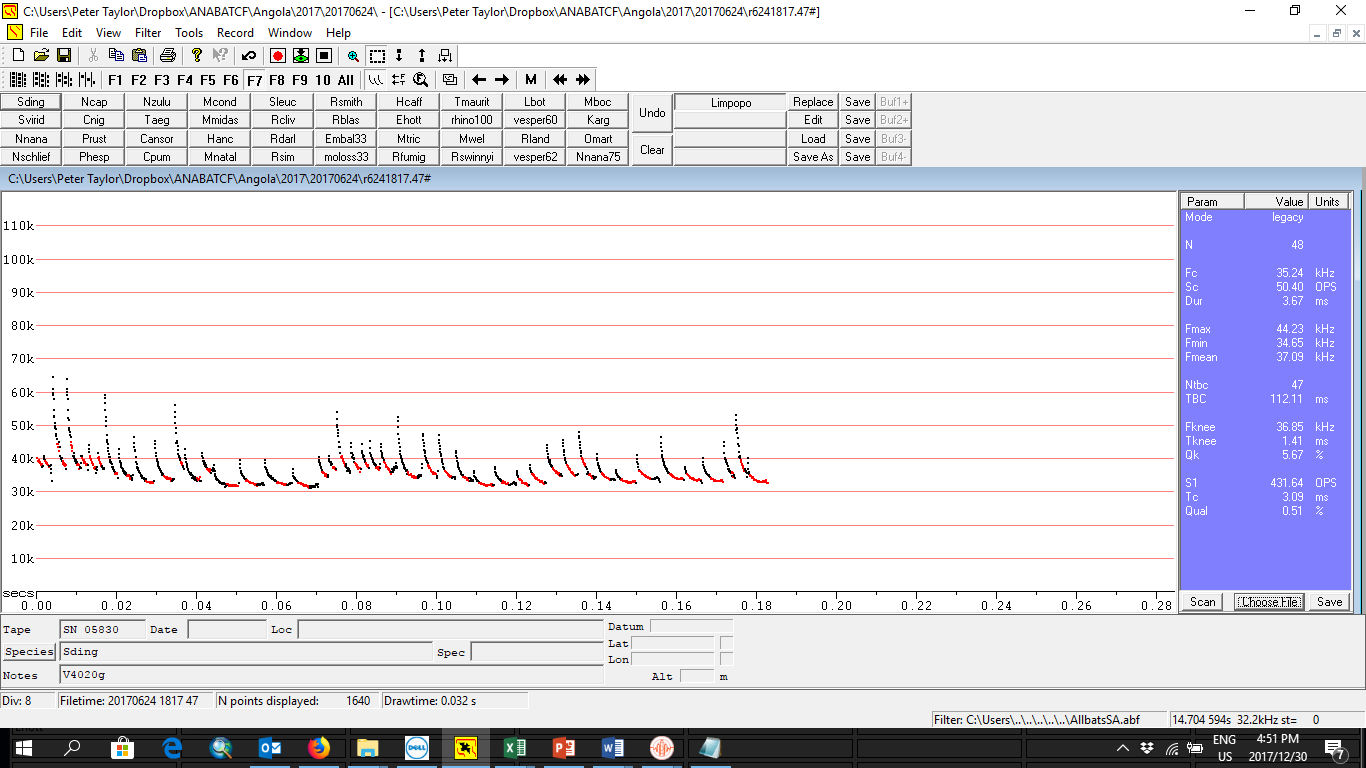


*Scotophilus dingani*


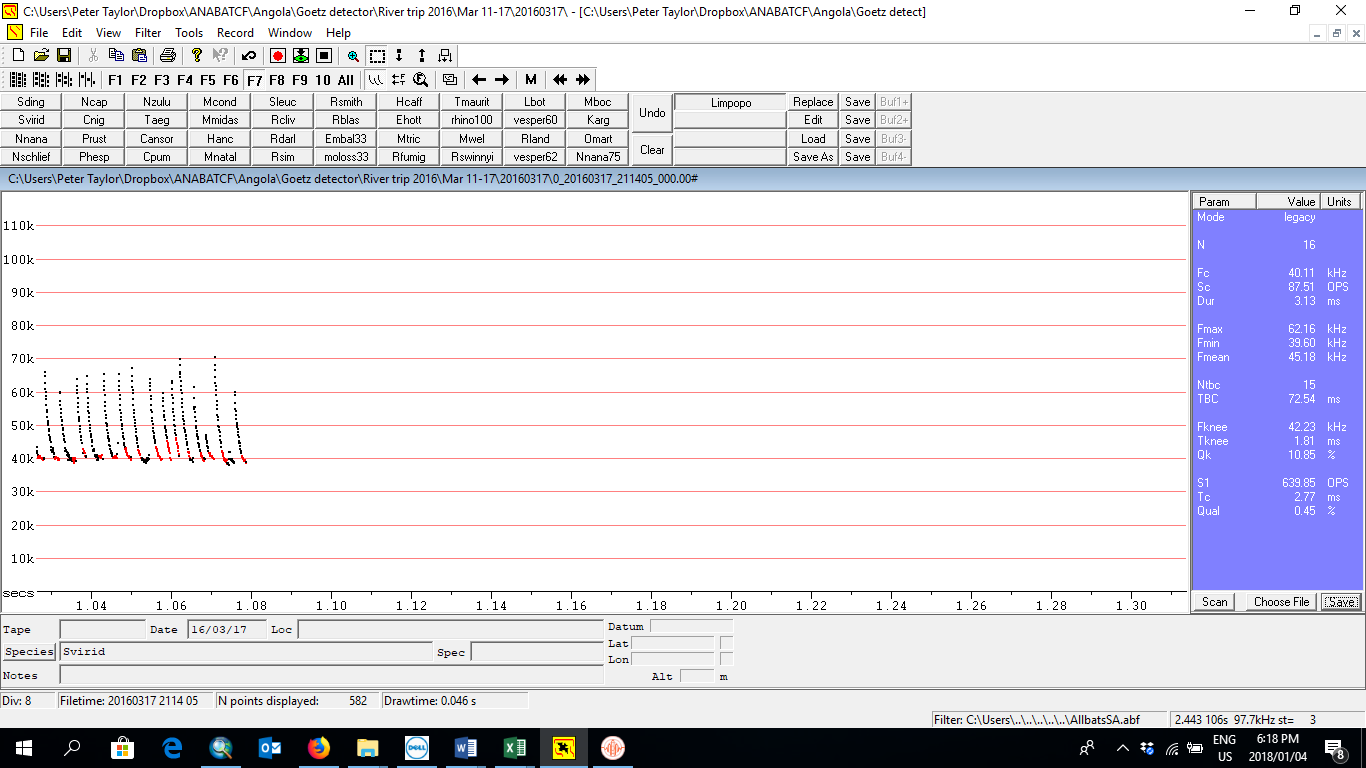


*Scotophilus leucogaster*


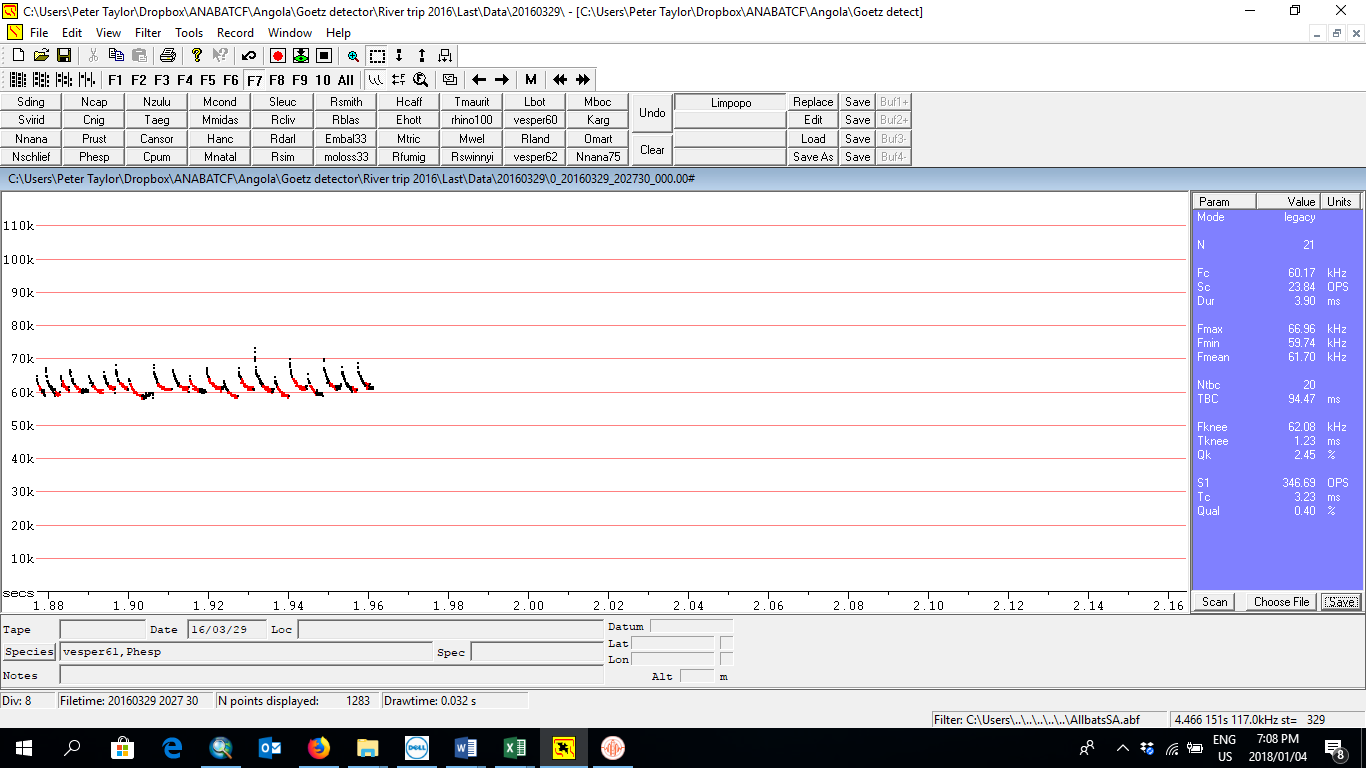


Vesper 61 (Miniopterus sp)

**Family Molossidae**


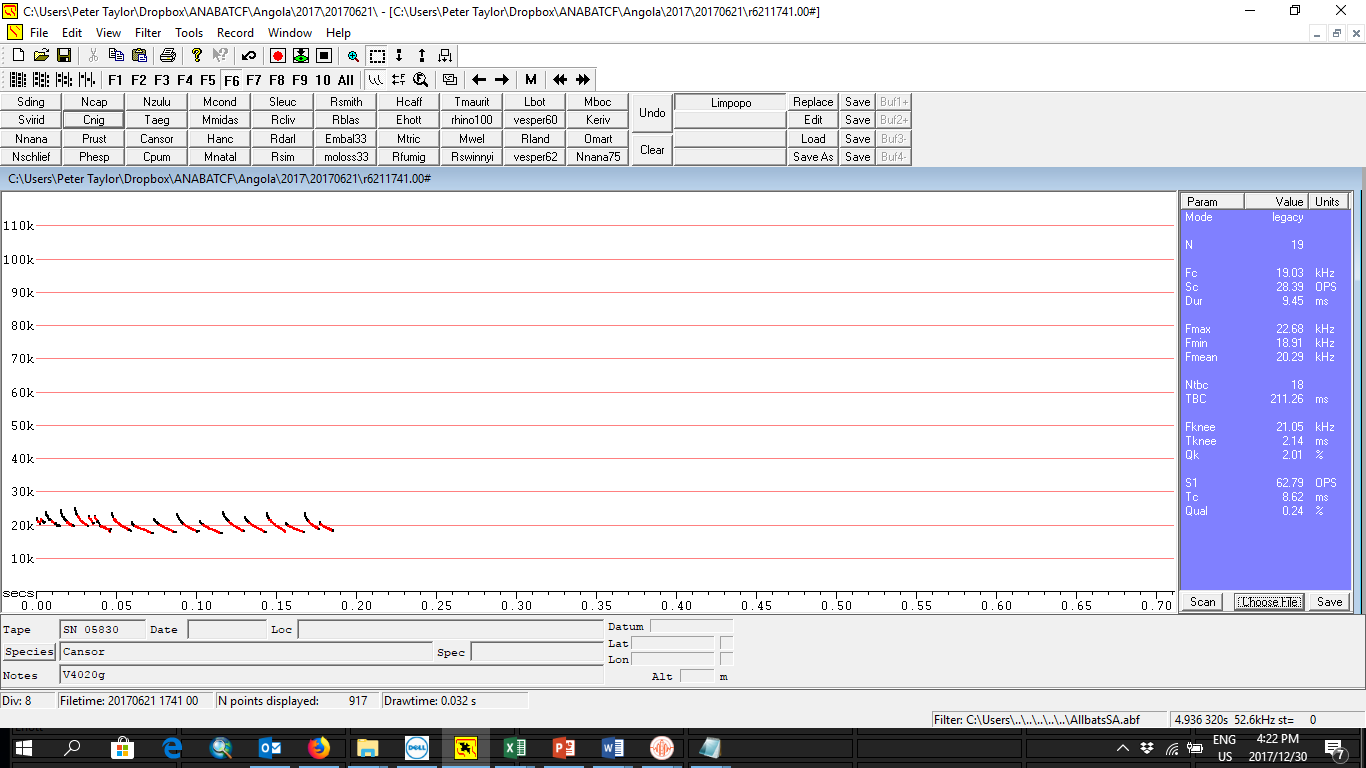


Chaerephon ansorgei


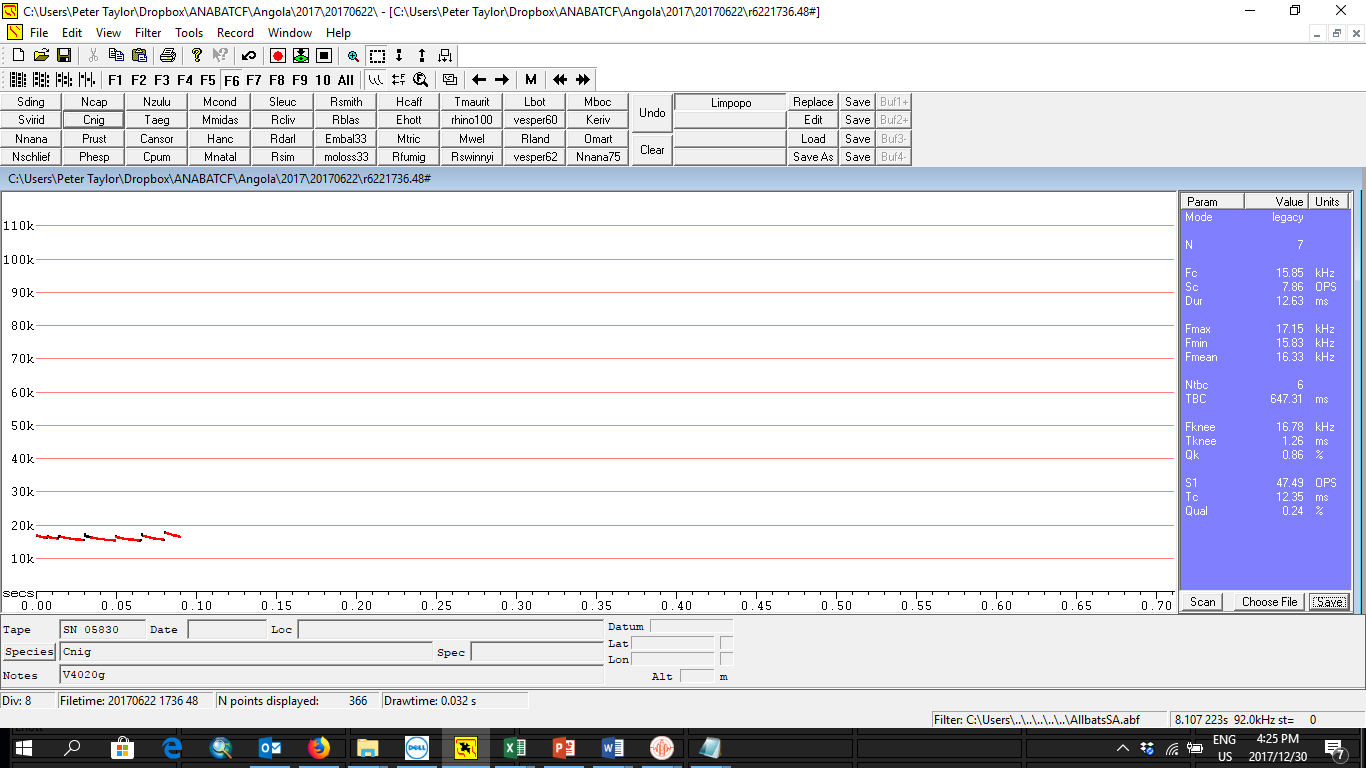


Chaerephon nigeriae 1


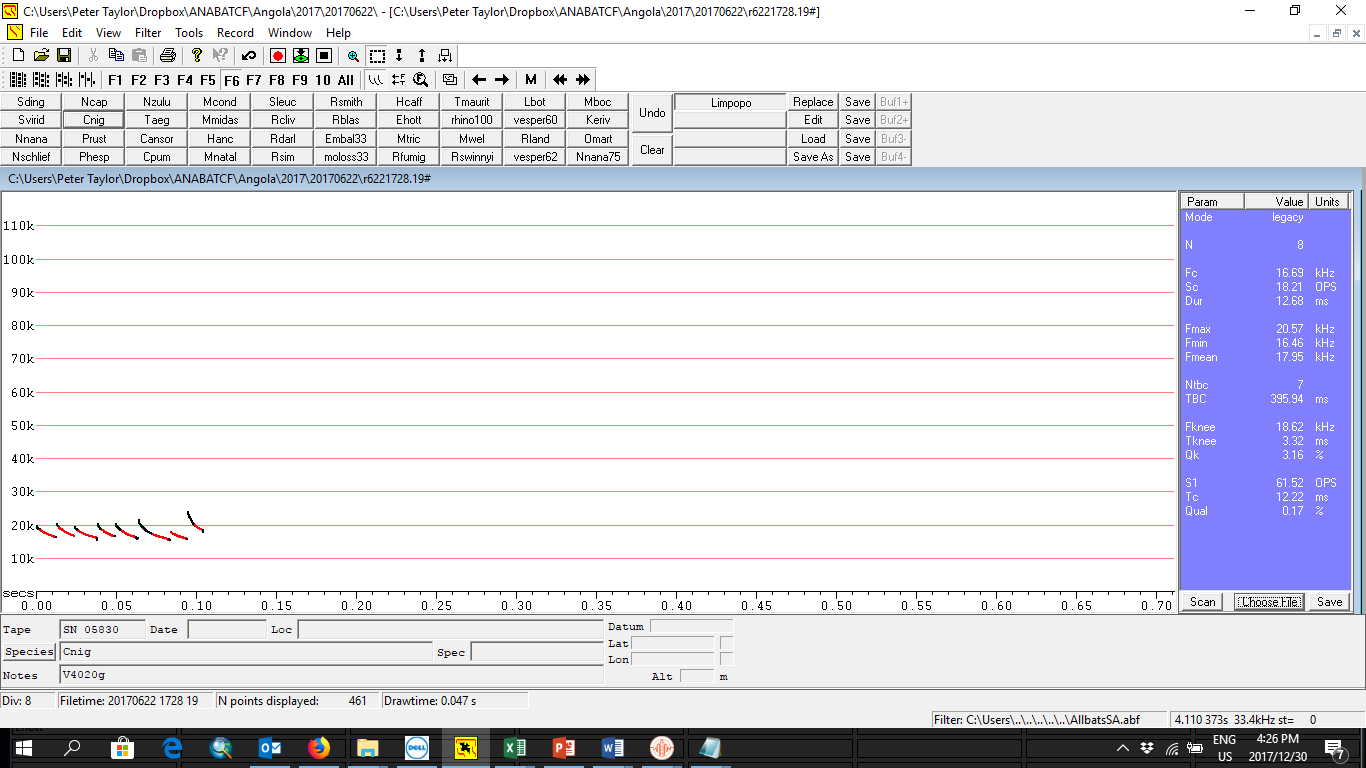


Chaerephon nigeriae2


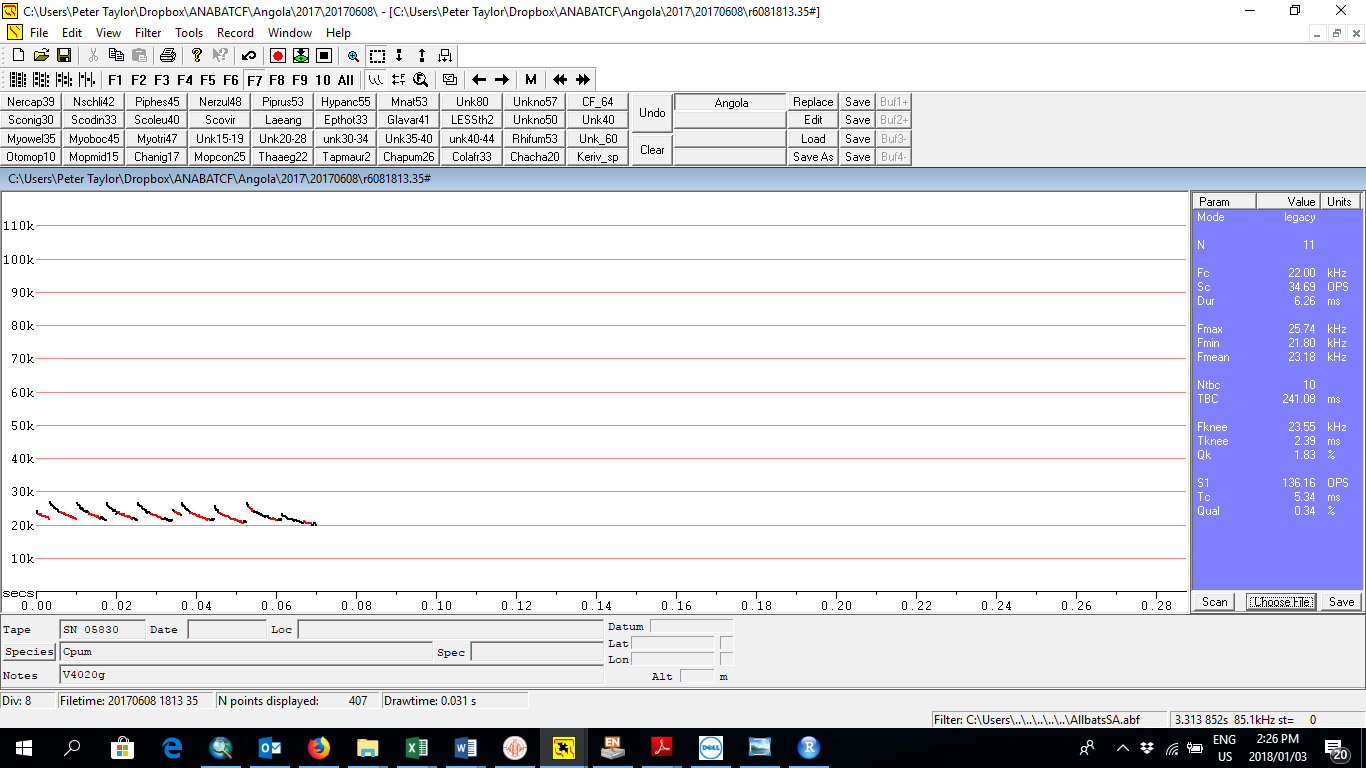


Chaerephon pumilus


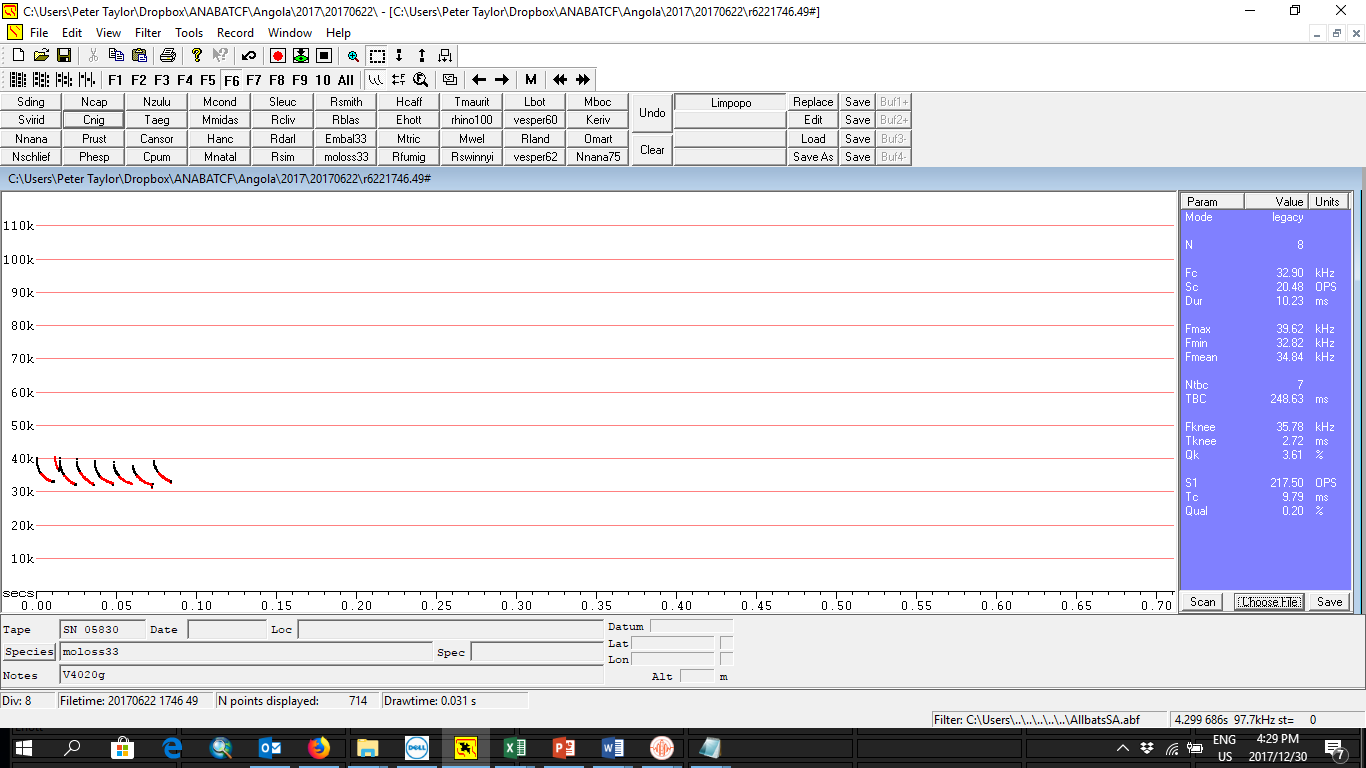


Molossid 33 kHz


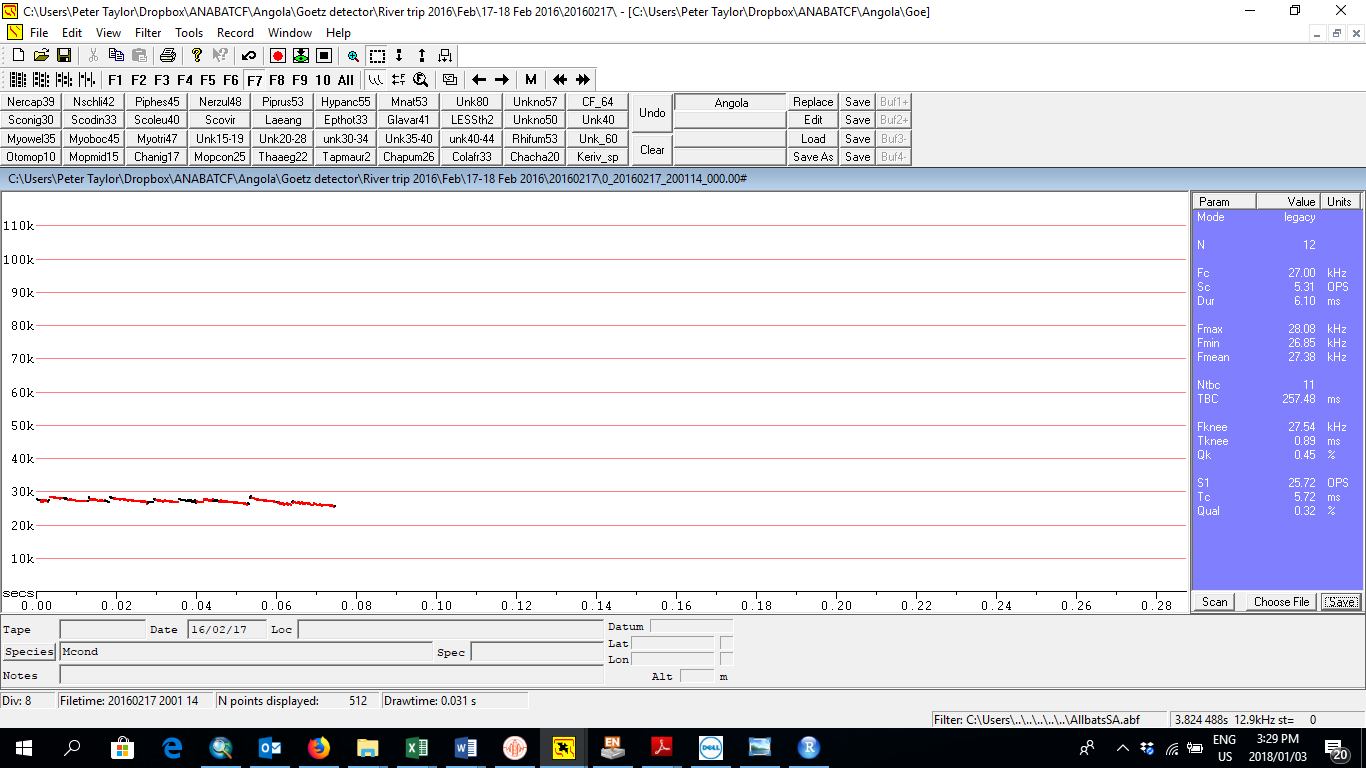


*Mops condylurus*


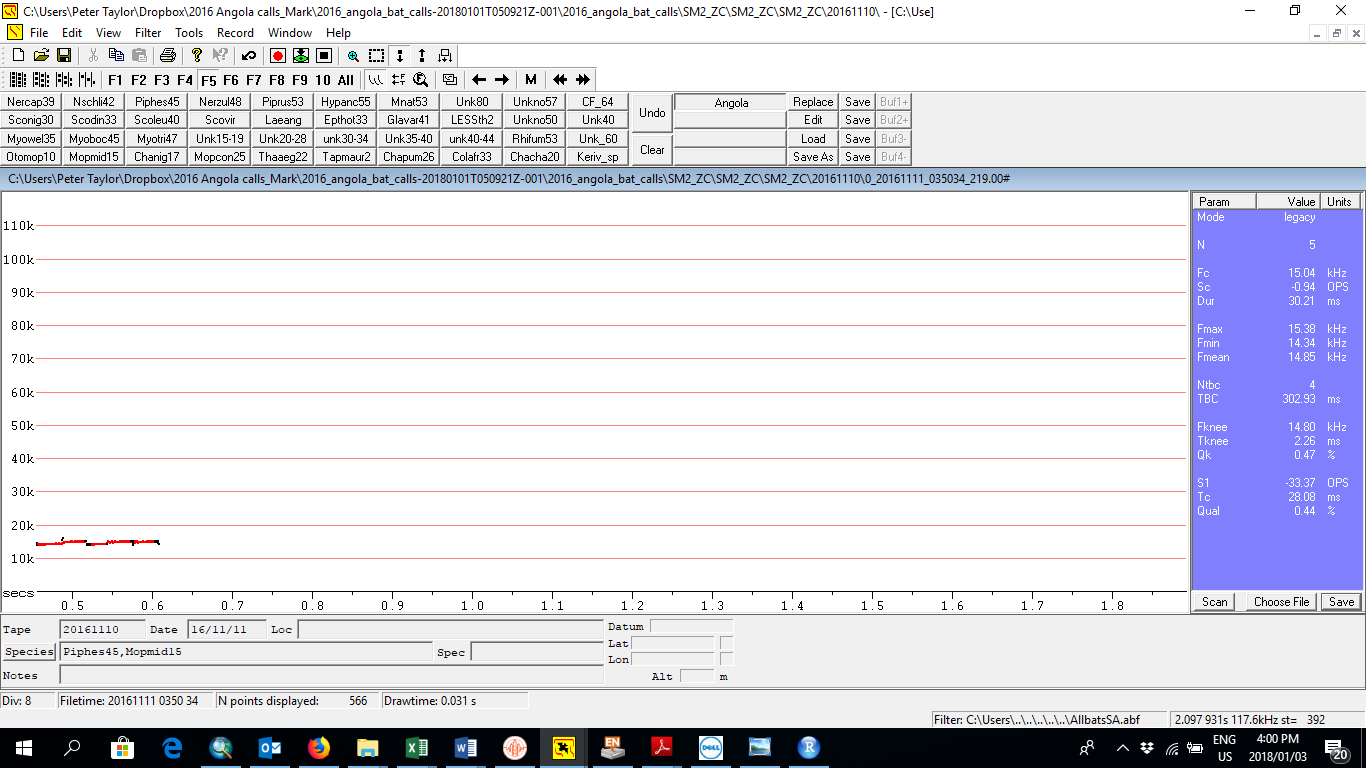


*Mops midas*


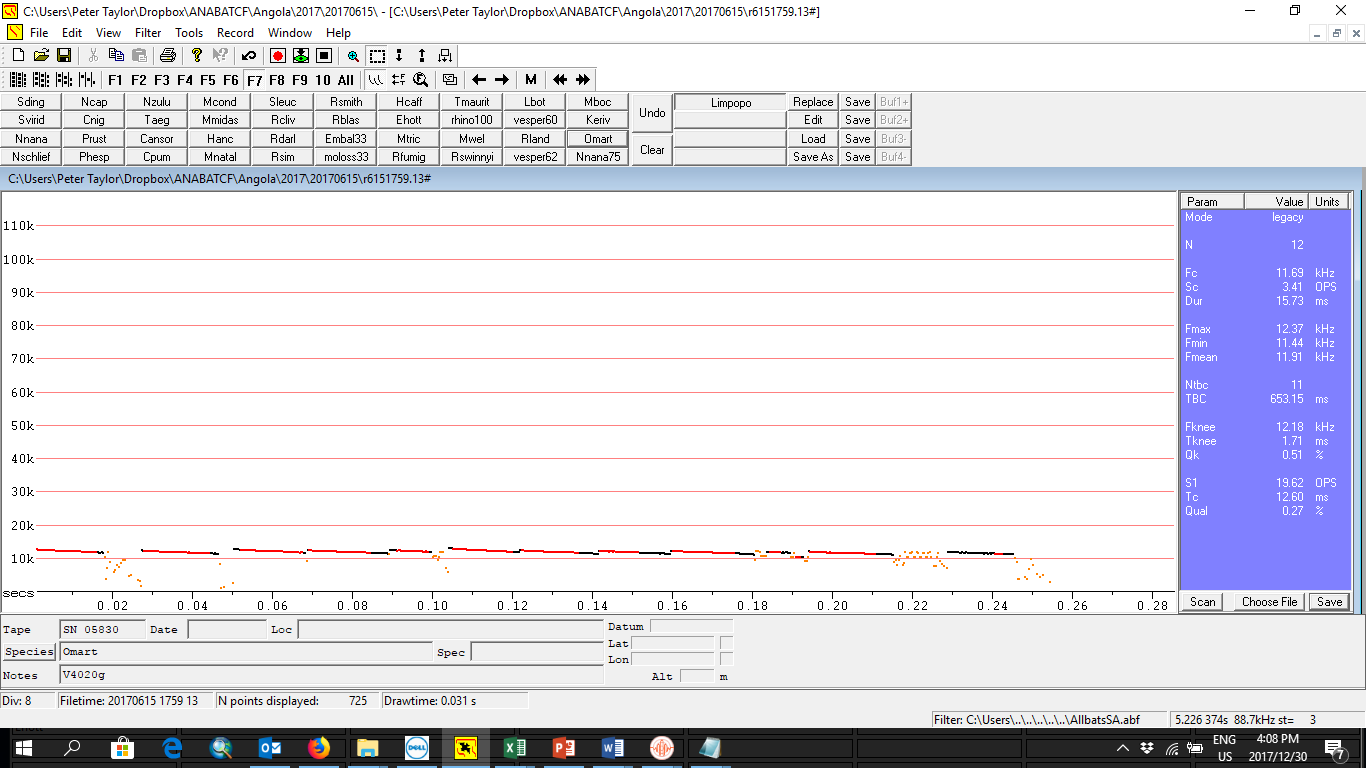


*Otomops martiensseni*


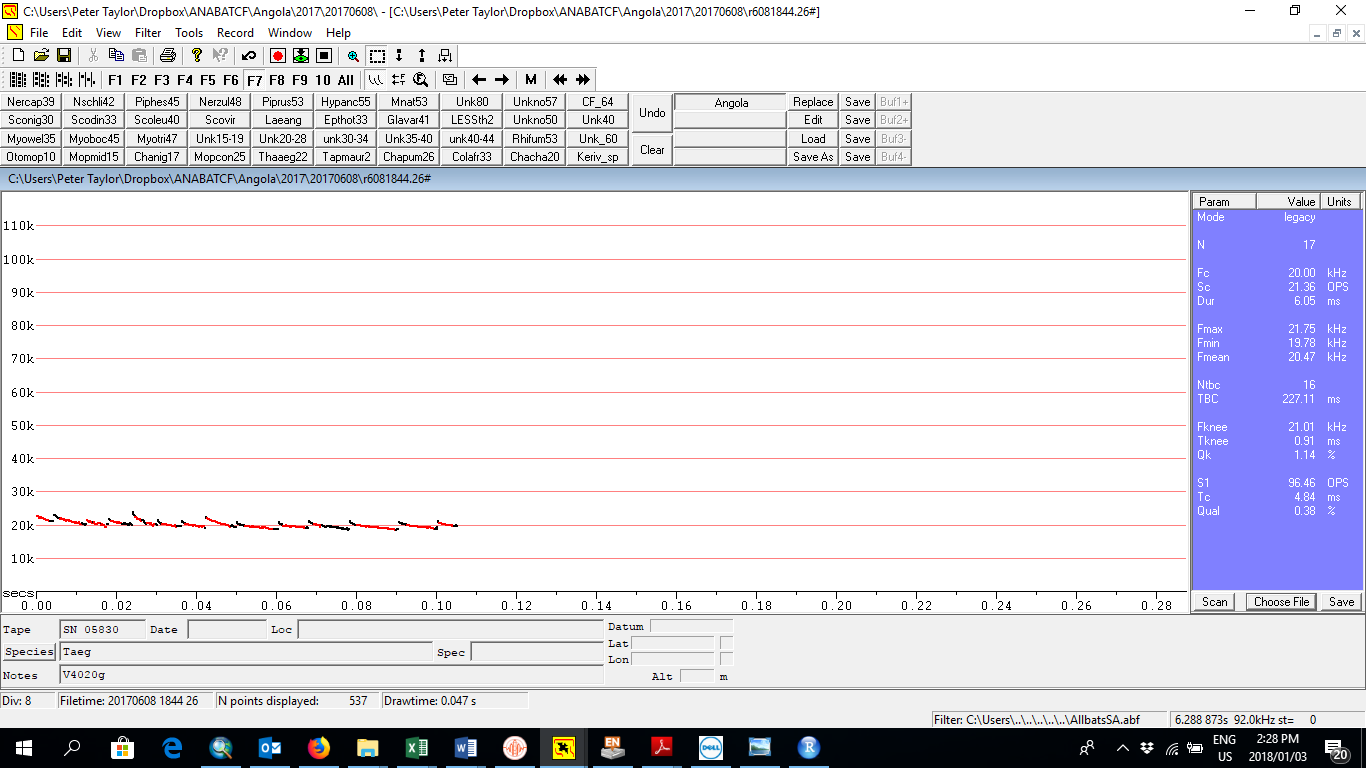


Tadarida aegyptiaca
